# A transmission dynamics model of COVID-19: Case of Cameroon

**DOI:** 10.1016/j.idm.2022.05.002

**Published:** 2022-05-24

**Authors:** Calvin Tadmon, Severin Foko

**Affiliations:** a Department of Mathematics and Computer Science, Faculty of Science, University of Dschang, P.O. Box: 67, Dschang, Cameroon; b The Abdus Salam International Centre for Theoretical Physics, Strada Costiera 11, 34151, Trieste, Italy

**Keywords:** COVID-19, ODE model, Global stability, Lyapunov function, Optimal control, 37B25, 34D23, 49J15

## Abstract

In this work, we propose and investigate an ordinary differential equations model describing the spread of COVID-19 in Cameroon. The model takes into account the asymptomatic, unreported symptomatic, quarantine, hospitalized individuals and the amount of virus in the environment, for evaluating their impact on the transmission of the disease. After establishing the basic properties of the model, we compute the control reproduction number $R_{c}$ and show that the disease dies out whenever $R_{c}\leq 1$ and is endemic whenever $R_{c}>1$. Furthermore, an optimal control problem is derived and investigated theoretically by mainly relying on Pontryagin's maximum principle. We illustrate the theoretical analysis by presenting some graphical results.

## 1 Introduction

Many countries around the world are facing a new pandemic disease that destroys their populations daily. This is Coronavirus Disease 2019 (COVID-19) caused by Severe Acute Respiratory Syndrome Coronavirus 2 (SARS-CoV-2). Once the virus is in contact with a healthy person, the infection is contracted and the virus, once within the host, moves to the surface of the lungs, creating an inflammation of the lungs called pneumonia. This causes the blockade of the respiratory system and alters the immune system. This situation can degenerate and lead to the death of the patient. This phenomenon occurs over a small period of time estimated approximately to seven days (Gandhi et al., 2020). The COVID-19 symptoms are highly variable and are associated with severe illnesses such as fever, severe cold, shortness of breath or dyspnoea, chills, cough, lymphopenia, expectoration, fatigue, headache, acute pneumonia, sputum production, diarrhoea, hemoptysis most often followed by renal failure (Carlos et al., 2020; CDC 2020; Huang et al., 2020; Ren et al., 2020; WHO, 2020a, 2020b). The virus spreads mainly through the environment whenever people are close to each other, or through contaminated surfaces. This occurs when an infected person in this environment breathes, coughs, sneezes or speaks and then virus-containing particles exhaled comes into contact with another person either through the mouth, nose or eyes (CDC 2020). The longer the people interact, the more likely they are to transmit COVID-19. This disease has resulted in prolonged population containment, paralyzing economies in several countries. The total number of deaths worldwide due to this pandemic has exceeded 1.65 million and the cumulative number of confirmed cases topped 74.7 million (Wikipedia, 2020a, 2020b). In Africa for example, the number of confirmed cases amounted to 2,404,414 representing approximately 3.3% global infection and the overall deaths attributable to COVID-19 was around 56.74 thousand (Galal, 2020a, 2020b). Note that as of May 13, 2020, every country in Africa has recorded a COVID-19 case. South Africa was the most drastically affected country, with more than 866.1 thousand confirmed COVID-19 cases and 23,451 deaths (Galal, 2020a, 2020b). COVID-19 was confirmed to have reached Cameroon on 6 March 2020, through an infected person from France; this French citizen has been quarantined in the Yaounde Central Hospital (Kouagheu, 2020). The Cameroonian Government has implemented a nationwide series of measures in order to curtail the spread of COVID-19. The steps and dates of deaths and confirmed COVID-19 cases as well as recovered cases can be seen in (Wikipedia, 2020a, 2020b). Especially, a national state of disaster was declared on April 17, 2020. Schools, training institutions and many other activities were closed on the same date. As of December 2020, more than 441 deaths were reported and the number of COVID-19 confirmed cases was approximately 24,560. It is worth noting that many infected people due to COVID-19 in Cameroon were unreported, since the scheduled door-to-door screening campaign was not totally operated. These statistics make Cameroon the epicenter of COVID-19 in Central Africa. The causes of the rapid spread of COVID-19 in Cameroon are given in (Ojong, 2020). This mainly includes: negligence of quarantine, refusal of isolation and lack of financial means to hospitalize all symptomatic people.

The COVID-19 pandemic that continues to be a threat, resulting in increasing suffering of population, deserves a rigorous study to eradicate it within the community. Several mathematical models have been proposed and studied in order to understand the transmission dynamics of this pandemic. In (Ivorra et al., 2020), the authors developed a *θ*-SEIHRD mathematical model which takes into account the known special characteristics of COVID-19 pandemic such as the existence of unreported symptomatic infectious individuals and the different sanitary and infectiousness conditions of hospitalized individuals. This *θ*-SEIHRD model was also used to estimate a significant number of beds needed in hospitals. Mohsin and co-workers (Mohsin et al., 2020) formulated a mathematical model that included asymptomatic, quarantine and isolation compartments, and showed that the high levels of quarantine and isolation need to be maintained for controlling the disease. They also proposed an optimal control problem applied to the dynamics described by the obtained model. Based on reported data from December 31, 2019 to January 28, 2020, Wu and co-authors (Wu et al., 2020) used a SEIR model to predict the national and global spread of COVID-19 in China. In (Yang et al., 2020), the authors proposed a modified SEIR model that investigated the epidemic development of COVID-19 in China; the authors foretold the timing and magnitude of the epidemic peak as well as the ultimate epidemic size. This model has been recommended as a practical example of mathematical modeling techniques to investigate the spread of the pandemic (Krishna, 2020). In (Tang et al., 2020), the mathematical model developed includes individual epidemiological status, intervention measures and clinical progression of COVID-19. The authors found that mediation strategies such as intensive contact tracing followed by quarantine and isolation can effectively curtail the transmission risk and the control reproduction number. In (Zhang et al., 2020), Zhang and his collaborators thought that the increase in new cases of COVID-19 is due to crowding factor. They developed a mathematical model by using a nonlinear incidence rate and taking into account the aforementioned factors. They applied a nonstandard finite difference (NSFD) scheme and the fourth order Runge-Kutta (RK4) scheme to obtain the graphical results.

The main purpose of the present work is to propose and investigate an ordinary differential equations (ODE) model describing the spread of COVID-19 in Cameroon, and use it to evaluate the impact of control measures, such as quarantine and hospitalization strategies, on the spread of the pandemic in this country which occupies a strategic position in Central Africa.

This paper is organized as follows. We formulate the ODE model in Section 2. Section 3 is devoted to the mathematical analysis of the proposed model. Specifically, we prove the existence, uniqueness, positivity and boundedness of the solution. In Section 4, we compute the control reproduction number and study the existence and stability properties of equilibria. Moreover, we analyze the control reproduction number around the quarantine of exposed individuals and the isolation of hospitalized individuals. In Section 5, we propose and investigate an optimal control problem associated to the model studied in Section 3. In section 6, we provide numerical simulations to illustrate the theoretical results obtained. We conclude the work in section 7.

## 2 Model formulation

The model considered in this study consists of the total number of individuals in a human population at time *t*, denoted by *N*(*t*), and sub-divided into eight distinct epidemiological subclasses of individuals, namely susceptible *S*(*t*), exposed *E*(*t*), asymptomatic infectious *A*(*t*), symptomatic infectious *I*(*t*), unreported symptomatic infectious *U*(*t*), quarantined *Q*(*t*), hospitalized *H*(*t*), recovered *R*(*t*)), and the concentration of virus in the environment at time *t*, denoted by *V*(*t*).

The dynamics description of each compartment is as follows.

Susceptible individuals, *S*, are recruited at a rate *s*, and decreased by natural death at a rate *μ*. Furthermore, as in (Safi and Gumel, 2010), we assume that the exposed class, *E*, and quarantined class, *Q*, do not transmit infection (i.e., exposed and quarantined individuals have a negligible number of contacts with members of the overall population; they play no role in the transmission process). So, it is assumed that only infected people presenting clinical symptoms can transmit the disease to others. Thus, the susceptible population *S* may acquire infection, following effective contact with infectious individuals in the I, A, U, H or V classes at a rate *λ*_*s*_, where

(2.1) $$\lambda_{s}=\beta \left(I+A+U+\eta_{1}H\right)+a_{0}V.$$

In equation (2.1), the parameter *β* is the average number of effective contacts between susceptible and infected individuals (symptomatic, asymptomatic, unreported symptomatic and hospitalized individuals), while 0 = *η*_1_ < 1 is the modification parameter which accounts for the assumed reduction in disease transmission by hospitalized individuals in comparison to non-hospitalized infectious individuals in the *I*, *A* and *U* classes. *η*_1_ measures the effectiveness of hospitalization; more precisely hospitalization is excellent if *η*_1_ = 0, leaky if 0 < *η*_1_ < 1 and completely ineffective if *η*_1_ = 1. Furthermore, we assume that the rate of transmissibility of the virus to the susceptible individuals is proportional to the free virus particles in the environment, and choose the force of infection as *a*_0_*V*. Thus, the rate of change of the susceptible population is expressed by the following equation:

$$\frac{dS}{dt}=s-\beta S\left(I+A+U+\eta_{1}H\right)-a_{0}SV-\mu S.$$

The population of exposed individuals, *E*, is generated by the infection of susceptible individuals at the rate *λ*_*s*_. This class is decreased due to reported clinical symptoms at the rate *η*, unreported clinical symptoms at the rate *b*, asymptomatic infectious at a rate *k*, quarantine at the rate *ϵ* and natural death at the rate *μ*, so that

$$\frac{dE}{dt}=\beta S\left(I+A+U+\eta_{1}H\right)+a_{0}SV-\left(\mu +k+\epsilon +b+\eta \right)E.$$

The population of asymptomatic infectious individuals, *A*, is generated at the rate *k*. It is decreased due to natural recovery at the rate *γ*, unreported clinical symptoms at the rate *θ*, natural death at the rate *μ* and disease-induced death at the rate *δ*_4_. This gives

$$\frac{dA}{dt}=kE-\left(\mu +\delta_{4}+\gamma +\theta \right)A.$$

The population of symptomatic infectious individuals, *I*, is generated at the rate *η*. This population is decreased due to natural recovery at the rate *ρ*, hospitalization at the rate *d*_0_, natural death at the rate *μ* and disease-induced death at the rate *δ*_1_. This is expressed as

$$\frac{dI}{dt}=\eta E-\left(\mu +\delta_{1}+\rho +d_{0}\right)I.$$

The population of unreported symptomatic infectious individuals, *U*, is generated by the exposed individuals at the rate *b* and the asymptomatic infectious individuals at the rate *θ*. This class is decreased due to natural recovery at the rate *ν*, natural death at the rate *μ* and disease-induced death at the rate *δ*_3_. So we have

$$\frac{dU}{dt}=bE+\theta A-\left(\mu +\delta_{3}+\nu \right)U.$$

Exposed individuals are quarantined at the rate *ϵ*. The population of quarantined individuals is decreased due to natural recovery at the rate *α*, hospitalization at the rate *d*_1_ and natural death at the rate *μ*. Thus, one has

$$\frac{dQ}{dt}=\epsilon E-\left(\mu +\alpha +d_{1}\right)Q.$$

The population of hospitalized individuals, *H*, is generated by the hospitalization of quarantined individuals at the rate *d*_1_, and symptomatic infectious individuals at the rate *d*_0_. This population is decreased due to recovery at the rate *r*, natural death at the rate *μ* and disease-induced death at the rate *δ*_2_. We can assume that *δ*_2_ < *δ*_1_, *δ*_2_ < *δ*_3_ and *δ*_2_ < *δ*_4_. This means that hospitalized individuals have reduced disease-induced mortality rate in comparison to non-hospitalized infectious individuals because of care given in hospitals. Thus, the rate of change of the population of hospitalized individuals is expressed by the following equation:

$$\frac{dH}{dt}=d_{0}I+d_{1}Q-\left(\mu +\delta_{2}+r\right)H.$$

The population of recovered individuals is generated by the recovery of asymptomatic infectious individuals at the rate *γ*, symptomatic infectious individuals at the rate *ρ*, unreported symptomatic infectious individuals at the rate *ν*, hospitalized infectious individuals at the rate *r* and quarantined individuals at the rate *α*. This population is decreased due to natural death at the rate *μ*. Therefore, we have the following equation:

$$\frac{dR}{dt}=\rho I+\nu U+\gamma A+\alpha Q+rH-\mu R.$$

Finally, the concentration of virus in the environment, *V*, is generated by the asymptomatic infectious individuals at the rate *ω*_1_, symptomatic infectious individuals at the rate *σ*, unreported symptomatic infectious individuals at the rate *ω*_0_ and hospitalized infectious individuals at the rates *a*_1_. It is decreased by inactivation at the rate *δ*_5_. Thus,

$$\frac{dV}{dt}=\sigma I+a_{1}H+\omega_{1}A+\omega_{0}U-\delta_{5}V.$$

**Fig. 1:**
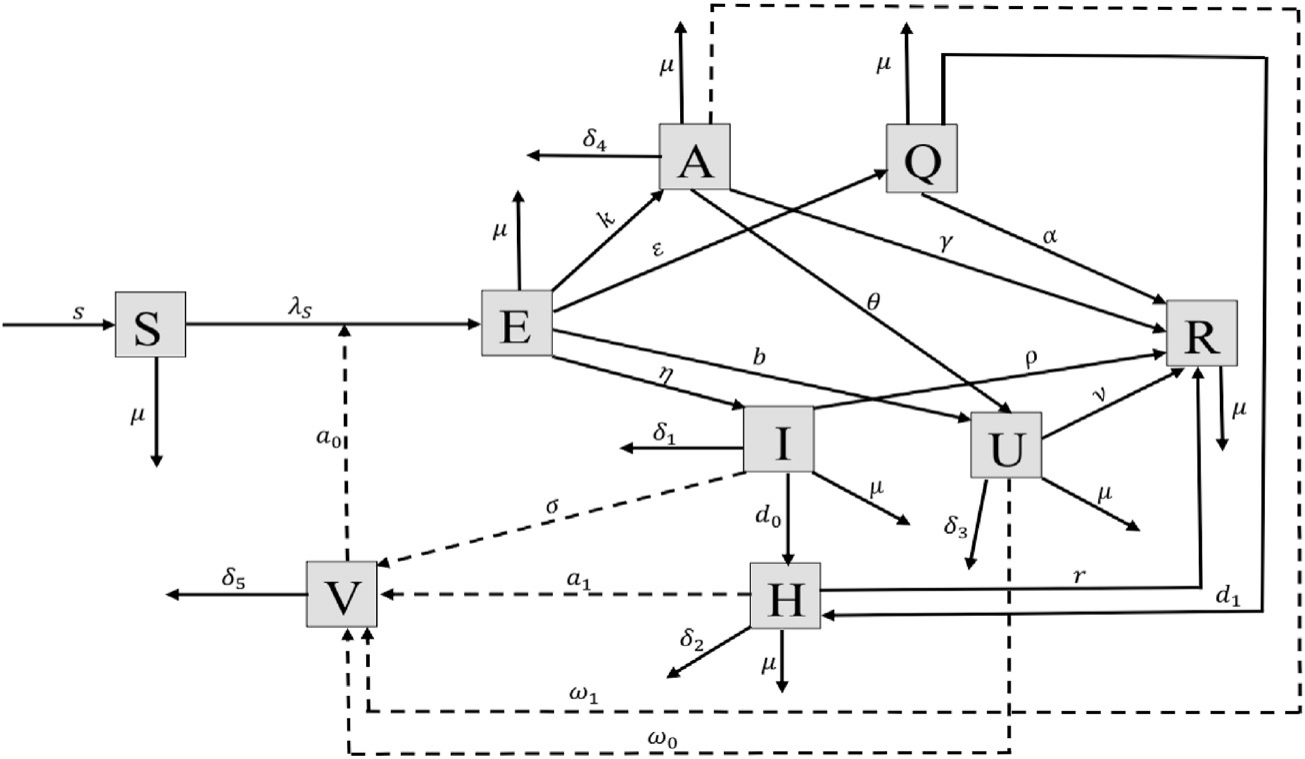
Flow diagram of the COVID-19 transmission model in Cameroon.

The flow diagram of the transmission dynamics of the COVID-19 is given in Fig. 1 below.

From the flow diagram in Fig. 1, we derive and propose the following nonlinear ODE system to describe the transmission dynamics of COVID-19 in Cameroon:

(2.2) $$\left\{\begin{array}{l} \frac{dS}{dt}=s-\beta S\left(I+A+U+\eta_{1}H\right)-a_{0}SV-\mu S,\;\\ \frac{dE}{dt}=\beta S\left(I+A+U+\eta_{1}H\right)+a_{0}SV-\left(\mu +k+\epsilon +b+\eta \right)E,\;\\ \frac{dA}{dt}=kE-\left(\mu +\delta_{4}+\gamma +\theta \right)A,\;\\ \frac{dI}{dt}=\eta E-\left(\mu +\delta_{1}+\rho +d_{0}\right)I,\;\\ \frac{dU}{dt}=bE+\theta A-\left(\mu +\delta_{3}+\nu \right)U,\;\\ \frac{dQ}{dt}=\epsilon E-\left(\mu +\alpha +d_{1}\right)Q,\;\\ \frac{dH}{dt}=d_{0}I+d_{1}Q-\left(\mu +\delta_{2}+r\right)H,\;\\ \frac{dV}{dt}=\sigma I+a_{1}H+\omega_{1}A+\omega_{0}U-\delta_{5}V,\;\\ \frac{dR}{dt}=\rho I+\nu U+\gamma A+\alpha Q+rH-\mu R,\; \end{array}\right)$$

with initial conditions:

(2.3) S(0)>0,E(0)>0,A(0)>0,I(0)>0,U(0)>0,Q(0)>0,H(0)>0,V(0)>0,R(0)>0.

The biological description of the parameters as well as their values and units are summed up in Table 1 below.

**Table 1:** Biological description, values and units of the parameters of model (2.2).

Parameter	Biological description of the parameters of model (2.2)	Value/range	Reference
*s*	Constant recruitment rate into the community	3539 *individual*.*day*^−1^	(Population Data)
*β*	Effective contact rate between susceptible and infected individuals	[3.62 × 10^−7^, 2 × 10^−6^] *day*^−1^	Estimated
*η*_1_	Modification parameter for reduction of infectiousness for	(0, 1] *day*^−1^	variable
	hospitalized individuals		
*η*	Progression rate from exposed to symptomatic infectious class	0.12405 *day*^−1^	Tang et al. (2020)
*ϵ*	Quarantine rate of exposed individuals	0.1 *day*^−1^	Assumed
*d*_1_	Hospitalization rate of quarantined individuals	0.04227 *day*^−1^	Assumed
*d*_0_	Hospitalization rate of symptomatic infectious individuals	0.20619 *day*^−1^	Assumed
*ρ*	Recovery rate of symptomatic infectious individuals	0.33029 *day*^−1^	Tang et al. (2020)
*r*	Recovery rate of hospitalized individuals	0.11624 *day*^−1^	Tang et al. (2020)
*δ*_1_	Disease-induced death rate of symptomatic infectious individuals	0.04227 *day*^−1^	Assumed
*δ*_2_	Disease-induced death rate of hospitalized individuals	0.027855 *day*^−1^	Assumed
*δ*_3_	Disease-induced death rate of unreported symptomatic	0.027855 *day*^−1^	Assumed
	infectious individuals		
*δ*_4_	Disease-induced death rate of asymptomatic infectious individuals	51 × 10^−4^*day*^−1^	Estimated
*δ*_5_	Decay rate of the virus	1/7 *day*^−1^	Estimated
*μ*	Natural death rate	[1/59, 1/57] *day*^−1^	WHO (2020a, 2020b)
*a*_0_	Transmission rate of the free virus	[10^−12^, 10^−7^] (*day*.*individual*)^−1^	Estimated
*b*	Progression rate from exposed to unreported symptomatic	(1–0.3)/7 *day*^−1^	Estimated
	infectious class		
*ν*	Recovery rate of unreported symptomatic infectious individuals	1/7 *day*^−1^	Liu et al. (2020)
*α*	Recovery rate of quarantined individuals	0.25 *day*^−1^	Estimated
*k*	Progression rate from exposed to asymptomatic infectious class	(1–1.8887 × 10^−7^)/7 *day*^−1^	Tang et al. (2020)
*ω*_0_	Shedding rate of the virus in the environment from unreported	4.65 × 10^−3^*virus*.(*day*.*individual*)^−1^	Estimated
	symptomatic infectious individuals		
*ω*_1_	Shedding rate of the virus in the environment from asymptomatic	10^−6^*virus*.(*day*.*individual*)^−1^	Estimated
	infectious individuals		
*θ*	Progression rate from asymptomatic infectious to unreported	(1–0.7)/7 *day*^−1^	Estimated
	symptomatic infectious class		
*γ*	Recovery rate of asymptomatic infectious individuals	0.13978 *day*^−1^	Tang et al. (2020)
*σ*	Shedding rate of the virus in the environment from	6.39 × 10^−3^*virus*.(*day*.*individual*)^−1^	Estimated
	symptomatic infectious individuals		

## 3 Basic properties of the full model

In this section, we explore the basic dynamical features of system (2.2). Since the COVID-19 model (2.2) monitors human populations, it will be epidemiologically meaningful if all its state variables are positive.

Theorem 3.1 *The solution* (S(t), E(t), A(t), I(t), U(t), Q(t), H(t), V(t), R(t)) *of system* (2.2) *starting from positive initial conditions* (2.3) *exists for all* t > 0 *and is unique*. *Furthermore*,

a) *S*(*t*) > 0*,* *E*(*t*) > 0*,* *A*(*t*) > 0*,* *I*(*t*) > 0*,* *U*(*t*) > 0*,* *Q*(*t*) > 0*,* *H*(*t*) > 0*,* *V*(*t*) > 0*, and* *R*(*t*) > 0*, for all time* *t* > 0*.*
b) *The biologically-feasible region* Ω*, defined by*

(3.1) $$\begin{array}{lll} \Omega =\left\{\left(S\left(t\right),E\left(t\right),A\left(t\right),I\left(t\right),U\left(t\right),Q\left(t\right),H\left(t\right),V\left(t\right),R\left(t\right)\right)\in R_{+}^{9}\;:\;\right) & & \\ \left(\;\;0<N\left(t\right)\leq \frac{s}{\mu },\;0\leq V\left(t\right)\leq \frac{s\left(\sigma +a_{1}+\omega_{1}+\omega_{0}\right)}{\mu \delta_{5}}\right\}, & & \end{array}$$

is positively invariant for model (2.2).

proof. *The proof uses classical arguments from the theory of ODEs* (Hale and Verduyn Lunel, 1993; Nkwayep et al., 2020). □ *From* Theorem 3.1, *it follows that in* Ω *the system* (2.2) *is well-posed mathematically and epidemiologically*. *Accordingly*, *it is sufficient to study the dynamics of the flow generated by system* (2.2) *in* Ω.

### 3.1 Existence and stability of equilibria

In this section, system (2.2) is analyzed to gain insight into its dynamical features.

### 3.2 Basic reproduction number and stability of the disease-free equilibrium (DFE)

The DFE of model (2.2) is obtained by setting the right hand sides of the equations to zero; it is given by:

(4.1) $$E_{0}=\left(S^{*},E^{*},A^{*},I^{*},U^{*},Q^{*},H^{*},V^{*},R^{*}\right)=\left(\frac{s}{\mu },0,0,0,0,0,0\right).$$

Now, to explore the local stability of $E_{0}$, we will use the next generation operator method developed in (Diekmann et al., 1990; van den Driessche and Watmough, 2002). More precisely, by using the matrix notation of Lemma 1 in (van den Driessche and Watmough, 2002), it follows that the matrix, *F*, of the new infection terms, and the non-singular *M*-matrix, *V*_1_, of the remaining transfer terms associated with model (2.2), are given, respectively, by

$$F=\left(\begin{array}{ccccccc} 0 & \frac{\beta s}{\mu } & \frac{\beta s}{\mu } & \frac{\beta s}{\mu } & 0 & \frac{\beta \eta_{1}s}{\mu } & \frac{sa_{0}}{\mu }\\ 0 & 0 & 0 & 0 & 0 & 0 & 0\\ 0 & 0 & 0 & 0 & 0 & 0 & 0\\ 0 & 0 & 0 & 0 & 0 & 0 & 0\\ 0 & 0 & 0 & 0 & 0 & 0 & 0\\ 0 & 0 & 0 & 0 & 0 & 0 & 0\\ 0 & 0 & 0 & 0 & 0 & 0 & 0 \end{array}\right)\;\text{and}\;V_{1}=\left(\begin{array}{ccccccc} k_{1} & 0 & 0 & 0 & 0 & 0 & 0\\ -k & k_{2} & 0 & 0 & 0 & 0 & 0\\ -\eta & 0 & k_{3} & 0 & 0 & 0 & 0\\ -b & -\theta & 0 & k_{4} & 0 & 0 & 0\\ -\epsilon & 0 & 0 & 0 & k_{5} & 0 & 0\\ 0 & 0 & -d_{0} & 0 & -d_{1} & k_{6} & 0\\ 0 & -\omega_{1} & -\sigma & -\omega_{0} & 0 & -a_{1} & \delta_{5} \end{array}\right).$$

It follows that the control reproduction number (Anderson & May 1982; Hethcote, 2000), denoted by $R_{c}=\rho \left(FV_{1}^{-1}\right)$, where $\rho \left(FV_{1}^{-1}\right)$ is the spectral radius of the next generation matrix $FV_{1}^{-1}$, is given by

$$\begin{array}{lll} & & R_{c}=\frac{\beta ks}{\mu k_{1}k_{2}}+\frac{\beta \eta s}{\mu k_{1}k_{3}}+\frac{\beta bs}{\mu k_{1}k_{4}}+\frac{\beta \theta ks}{\mu k_{1}k_{2}k_{4}}+\frac{\beta \eta_{1}\eta d_{0}s}{\mu k_{1}k_{3}k_{6}}+\frac{\beta \eta_{1}\epsilon d_{1}s}{\mu k_{1}k_{5}k_{6}}+\frac{k\theta a_{0}\omega_{0}s}{\mu k_{1}k_{2}k_{4}\delta_{5}}\\ & & \;\;+\frac{ka_{0}\omega_{1}s}{\mu k_{1}k_{2}\delta_{5}}+\frac{\eta d_{0}a_{0}a_{1}s}{\mu k_{1}k_{3}k_{6}\delta_{5}}+\frac{\eta \sigma a_{0}s}{\mu k_{1}k_{3}\delta_{5}}+\frac{ba_{0}\omega_{0}s}{\mu k_{1}k_{4}\delta_{5}}+\frac{\epsilon d_{1}a_{0}a_{1}s}{\mu k_{1}k_{5}k_{6}\delta_{5}}, \end{array}$$

where

(4.2) $$\begin{array}{c} k_{1}=\mu +k+\epsilon +b+\eta ,\;k_{2}=\mu +\delta_{4}+\gamma +\theta ,\;k_{3}=\mu +\delta_{1}+\rho +d_{0},\;\\ k_{4}=\mu +\delta_{3}+\nu ,\;k_{5}=\mu +\alpha +d_{1}\;\text{and}\;k_{6}=\mu +\delta_{2}+r. \end{array}$$

The epidemiological meaning of the quantity $R_{c}$ (reproduction number of the full model with control measures) is that, it measures the average number of new COVID-19 positive cases generated by a single typical COVID-19-infected individual (living or dead) introduced into a completely-susceptible human population. This infers that, COVID-19 can be effectively controlled in the community if the threshold quantity $R_{c}$ is less than unity (i.e. $R_{c}<1$). Thus, COVID-19 cannot develop from a small influx of infected individuals if $R_{c}<1$, but COVID-19 will develop if $R_{c}>1$. Now, the epidemiological interpretation of each term of $R_{c}$ is as follows. First, the mean duration of an infective individual in class *E* is 1/*k*_1_. A fraction *k*/*k*_1_ of infective individuals moves from class *E* into class *A* with effective contact rate *β* and mean duration 1/*k*_2_, offering a contribution of *βks*/*μk*_1_*k*_2_ to $R_{c}$. Next, a fraction *η*/*k*_1_ of infective individuals moves from class *E* into class *I*, with effective contact rate *β* and mean duration 1/*k*_3_, offering a contribution of *βηs*/*μk*_1_*k*_3_ to $R_{c}$. A fraction *b*/*k*_1_ moves from class *E* into class *U* with effective contact rate *β* and mean duration 1/*k*_4_, giving a contribution of *βbs*/*μk*_1_*k*_4_, and after a severity of infection, a fraction *θk*/*k*_1_*k*_2_ moves from class *A* into class *U*, giving a contribution of *βθks*/*μk*_1_*k*_2_*k*_4_ to $R_{c}$. A fraction *ϵ*/*k*_1_ moves from *E* to *Q* and the mean duration of *Q* is 1/*k*_5_. A fraction *ηd*_0_/*k*_1_*k*_3_ moves from *E* to *I* then to *H* with effective contact rate *βη*_1_ and mean duration 1/*k*_6_, offering a contribution of *βη*_1_*ηd*_0_*s*/*μk*_1_*k*_3_*k*_6_ to $R_{c}$. A fraction *ϵd*_1_/*k*_1_*k*_5_ moves from *E* to *Q* then to *H* with effective contact rate *βη*_1_ and mean duration 1/*k*_6_, offering a contribution of *βη*_1_*ϵd*_1_*s*/*μk*_1_*k*_5_*k*_6_ to $R_{c}$. A fraction *kθω*_0_/*k*_1_*k*_2_*k*_4_ moves from *E* to *A* then to *U* and to *V* with effective contact rate *a*_0_ and mean duration 1/*δ*_5_, giving a contribution of *kθa*_0_*ω*_0_*s*/*μk*_1_*k*_2_*k*_4_*δ*_5_ to $R_{c}$. A fraction *kω*_1_/*k*_1_*k*_2_ moves from *E* to *A* then to *V* with effective contact rate *a*_0_ and mean duration 1/*δ*_5_, giving a contribution of *ka*_0_*ω*_1_*s*/*μk*_1_*k*_2_*δ*_5_ to $R_{c}$. A fraction *ηd*_0_*a*_1_/*k*_1_*k*_3_*k*_6_ moves from *E* to *I* then to *H* and to *V* with effective contact rate *a*_0_ and mean duration 1/*δ*_5_, giving a contribution of *ηd*_0_*a*_0_*a*_1_*s*/*μk*_1_*k*_3_*k*_6_*δ*_5_ to $R_{c}$. A fraction *ησ*/*k*_1_*k*_3_ moves from *E* to *I* then to *V* with effective contact rate *a*_0_ and mean duration 1/*δ*_5_, giving a contribution of *ησa*_0_*s*/*μk*_1_*k*_3_*δ*_5_ to $R_{c}$. A fraction *bω*_0_/*k*_1_*k*_4_ moves from *E* to *U* then to *V* with effective contact rate *a*_0_ and mean duration 1/*δ*_5_, giving a contribution of *ba*_0_*ω*_0_*s*/*μk*_1_*k*_4_*δ*_5_ to $R_{c}$. Finally, a fraction *ϵd*_1_*a*_1_/*k*_1_*k*_5_*k*_6_ moves from *E* to *Q* then to *H* and to *V* with effective contact rate *a*_0_ and mean duration 1/*δ*_5_, giving a contribution of *ϵd*_1_*a*_0_*a*_1_*s*/*μk*_1_*k*_5_*k*_5_*δ*_5_ to $R_{c}$.

Note that the basic reproduction number $R_{0}$ is defined in the absence of control measures such as quarantine, isolation and environmental spraying techniques to disinfect exposed surfaces. Thus $R_{0}$ is $R_{c}$ with *ϵ* = *d*_0_ = *a*_1_ = *σ* = *ω*_0_ = *ω*_1_ = 0. It then follows that

$$R_{0}=\frac{\beta ks}{\mu k_{01}k_{02}}+\frac{\beta \eta s}{\mu k_{01}k_{03}}+\frac{\beta bs}{\mu k_{01}k_{04}}+\frac{\beta \theta ks}{\mu k_{01}k_{02}k_{04}},$$

where

$$\begin{array}{c} k_{01}=\mu +k+b+\eta ,\;k_{02}=\mu +\delta_{4}+\gamma +\theta ,\;k_{03}=\mu +\delta_{1}+\rho ,\;\text{and}\;k_{04}=\mu +\delta_{3}+\nu . \end{array}$$

The following result is obtained by using similar arguments as in the proof of Theorem 2 in (van den Driessche and Watmough, 2002).

Lemma 4.1 *The DFE,*$E_{0}$*, of system* (2.2)*, given by* (4.1)*, is locally asymptotically stable in* Ω *whenever* $R_{c}<1$*, and unstable if* $R_{c}>1$*.*

*Proof*. *Linearizing* (2.2) *at the DFE* $E_{0}$, *we obtain the linearized system*

$$\frac{dW}{dt}=J\left(E_{0}\right)W,$$

*where* *W* = (*S*, *E*, *A*, *I*, *U*, *Q*, *H*, *V*, *R*) *and*

$$J\left(E_{0}\right)=\left(\begin{array}{ccccccccc} -\mu & 0 & -\frac{\beta s}{\mu } & -\frac{\beta s}{\mu } & -\frac{\beta s}{\mu } & 0 & -\frac{\beta \eta_{1}s}{\mu } & -\frac{sa_{0}}{\mu } & 0\\ 0 & -k_{1} & \frac{\beta s}{\mu } & \frac{\beta s}{\mu } & \frac{\beta s}{\mu } & 0 & \frac{\beta \eta_{1}s}{\mu } & \frac{sa_{0}}{\mu } & 0\\ 0 & k & -k_{2} & 0 & 0 & 0 & 0 & 0 & 0\\ 0 & \eta & 0 & -k_{3} & 0 & 0 & 0 & 0 & 0\\ 0 & b & \theta & 0 & -k_{4} & 0 & 0 & 0 & 0\\ 0 & \epsilon & 0 & 0 & 0 & -k_{5} & 0 & 0 & 0\\ 0 & 0 & 0 & d_{0} & 0 & d_{1} & -k_{6} & 0 & 0\\ 0 & 0 & \omega_{1} & \sigma & \omega_{0} & 0 & a_{1} & -\delta_{5} & 0\\ 0 & 0 & \gamma & \rho & \nu & \alpha & r & 0 & -\mu \end{array}\right).$$

*Now*, *to end the proof*, *it is necessary to prove that all eigenvalues of the Jacobian matrix*, $J\left(E_{0}\right)$, *have negative real parts*. *So*, *writing the Jacobian matrix*, $J\left(E_{0}\right)$, *under the distributed matrix form*, *we obtain*

$$\left(\begin{array}{ccccccccc} -\mu & c_{1} & c_{2} & 0 & 0 & 0 & 0 & 0 & 0\\ 0 & c_{3} & c_{4} & 0 & 0 & 0 & 0 & 0 & 0\\ 0 & k & -k_{2} & 0 & 0 & 0 & 0 & 0 & 0\\ 0 & \eta & 0 & -k_{3} & 0 & 0 & 0 & 0 & 0\\ 0 & b & \theta & 0 & -k_{4} & 0 & 0 & 0 & 0\\ 0 & \epsilon & 0 & 0 & 0 & -k_{5} & 0 & 0 & 0\\ 0 & 0 & 0 & d_{0} & 0 & d_{1} & -k_{6} & 0 & 0\\ 0 & 0 & \omega_{1} & \sigma & \omega_{0} & 0 & a_{1} & -\delta_{5} & 0\\ 0 & 0 & \gamma & \rho & \nu & \alpha & r & 0 & -\mu \end{array}\right),$$

*where*

$c_{1}=-\frac{sa_{0}a_{1}d_{1}\epsilon }{\mu k_{5}k_{6}\delta_{5}}-\frac{\beta \eta_{1}d_{1}s\epsilon }{\mu k_{5}k_{6}}-\frac{sa_{0}\omega_{0}b}{\mu k_{4}\delta_{5}}-\frac{\beta bs}{\mu k_{4}}-\frac{sa_{0}\sigma \eta }{\mu k_{3}\delta_{5}}-\frac{\beta \eta s}{\mu k_{3}}-\frac{sa_{0}a_{1}d_{0}\eta }{\mu k_{3}k_{6}\delta_{5}}-\frac{\beta \eta_{1}d_{0}s\eta }{\mu k_{3}k_{6}}$, $c_{2}=-\frac{\beta s}{\mu }-\frac{sa_{0}\omega_{1}}{\mu \delta_{5}}-\frac{sa_{0}\omega_{0}\theta }{\mu k_{4}\delta_{5}}-\frac{\beta \theta s}{\mu k_{4}}$,
$c_{3}=-\frac{1}{k_{2}}+\frac{sa_{0}a_{1}d_{1}\epsilon }{\mu k_{1}k_{2}k_{3}k_{5}k_{6}\delta_{5}}+\frac{\beta \eta_{1}d_{1}s\epsilon }{\mu k_{1}k_{2}k_{5}k_{6}}+\frac{sa_{0}\omega_{0}b}{\mu k_{1}k_{2}k_{4}\delta_{5}}+\frac{\beta bs}{\mu k_{1}k_{2}k_{4}}+\frac{sa_{0}\sigma \eta }{\mu k_{1}k_{2}k_{3}\delta_{5}}$
$\;\;+\frac{\beta \eta s}{\mu k_{1}k_{2}k_{3}}+\frac{sa_{0}a_{1}d_{0}\eta }{\mu k_{1}k_{2}k_{3}k_{6}\delta_{5}}+\frac{\beta \eta_{1}d_{0}s\eta }{\mu k_{1}k_{2}k_{3}k_{6}}$,
$c_{4}=\frac{\beta s}{\mu k_{1}k_{2}}+\frac{sa_{0}\omega_{1}}{\mu k_{1}k_{2}\delta_{5}}+\frac{sa_{0}\omega_{0}\theta }{\mu k_{1}k_{2}k_{4}\delta_{5}}+\frac{\beta \theta s}{\mu k_{1}k_{2}k_{4}}$.

*LetMbe the following three dimensional matrix defined by*

$$M=\left(\begin{array}{ccc} -\mu & c_{1} & c_{2}\\ 0 & c_{3} & c_{4}\\ 0 & k & -k_{2} \end{array}\right).$$

*Note that*, *all eigenvalues of the Jacobian matrix*, $J\left(E_{0}\right)$, *have negative real parts whenever* det(*M*) < 0.

*The computation of* det(*M*), *gives*

$$\begin{array}{lll} & & \det\left(M\right)=\mu \left(kc_{4}+k_{2}c_{3}\right),\\ & & \;\;=\mu \left(\frac{\beta ks}{\mu k_{1}k_{2}}+\frac{\beta \eta s}{\mu k_{1}k_{3}}+\frac{\beta bs}{\mu k_{1}k_{4}}+\frac{\beta \theta ks}{\mu k_{1}k_{2}k_{4}}+\frac{\beta \eta_{1}\eta d_{0}s}{\mu k_{1}k_{3}k_{6}}+\frac{\beta \eta_{1}\epsilon d_{1}s}{\mu k_{1}k_{5}k_{6}}+\frac{k\theta a_{0}\omega_{0}s}{\mu k_{1}k_{2}k_{4}\delta_{5}}\right)\\ & & \;\;\;\;\left(+\frac{ka_{0}\omega_{1}s}{\mu k_{1}k_{2}\delta_{5}}+\frac{\eta d_{0}a_{0}a_{1}s}{\mu k_{1}k_{3}k_{6}\delta_{5}}+\frac{\eta \sigma a_{0}s}{\mu k_{1}k_{3}\delta_{5}}+\frac{ba_{0}\omega_{0}s}{\mu k_{1}k_{4}\delta_{5}}+\frac{\epsilon d_{1}a_{0}a_{1}s}{\mu k_{1}k_{5}k_{6}\delta_{5}}-1\right),\\ & & \;\;=\mu \left(R_{c}-1\right). \end{array}$$

*So if*$R_{c}<1$, *it follows that* det(*M*) < 0. *In this case*, *all eigenvalues of the Jacobian matrix* $J\left(E_{0}\right)$ *have negative real parts*. *Thus*, *if* $R_{c}<1$, *the DFE*, $E_{0}$, *of system* (2.2), *given by* (4.1), *is locally asymptotically stable*. *If* $R_{c}>1$, *then* det(*M*) > 0. *This infers that*, *there exists an eigenvalue of the Jacobian matrix* $J\left(E_{0}\right)$ *with positive real part*. *So*, *if* $R_{c}>1$, *then* $E_{0}$ *is unstable*. *This completes the proof*. □

Remark 4.2 *Lemma* 4.1 *communicates that COVID-19* is eliminated from the population (when $R_{c}<1$) if the initial sizes of the sub-populations of the obtained system are in the basin of attraction of the DFE $E_{0}$*.* In what follows, to ensure that COVID-19 is eliminated from the population regardless of the initial sizes of the sub-populations, we need to prove the global stability of $E_{0}$*.*

### 3.3 **Global stability of the DFE**$E_{0}$

In this section, we investigate the global stability of the DFE, $E_{0}$, by constructing a suitable Lyapunov functional and using LaSalle's invariance principle. For this purpose, consider the following function defined for positive real numbers by

(4.3) g(x)=x−1−lnx.

It can be shown that g(*x*) ≥ 0 for all *x* > 0, and that min_*x*>0_g(*x*) = g(1) = 0.

We have the following result.

Theorem 4.3 *The DFE*, $E_{0}$, *of system* (2.2), *given by* (4.1), *is globally asymptotically stable in* Ω *whenever* $R_{c}\leq 1$.

*Proof*. *Let* (*S*(*t*), *E*(*t*), *A*(*t*), *I*(*t*), *U*(*t*), *Q*(*t*), *H*(*t*), *V*(*t*), *R*(*t*)) *be any positive solution of system* (2.2) *in* Ω. *Recall that* *S*∗ = *s*/*μ*. *Define the following Lyapunov function*

(4.4) $$\begin{array}{lll} & & L\left(t\right)=\frac{\delta_{5}S^{*}R_{c}}{\beta \eta_{1}}\left(\frac{S}{S^{*}}-1-\ln\frac{S}{S^{*}}\right)+\frac{\delta_{5}R_{c}}{\beta \eta_{1}}E+\frac{k_{4}\beta \delta_{5}S^{*}+k_{4}a_{0}\omega_{1}S^{*}+a_{0}\theta \omega_{0}S^{*}+\beta \theta \delta_{5}S^{*}}{\beta \eta_{1}k_{2}k_{4}}A\\ & & \;\;+\frac{k_{6}\beta \delta_{5}S^{*}+a_{0}a_{1}d_{0}S^{*}+a_{0}\sigma k_{6}S^{*}+\beta \eta_{1}d_{0}\delta_{5}S^{*}}{\beta \eta_{1}k_{3}k_{6}}I+\frac{\beta \delta_{5}S^{*}+a_{0}\omega_{0}S^{*}}{\beta \eta_{1}k_{4}}U\\ & & \;\;+\frac{\beta \eta_{1}d_{1}\delta_{5}S^{*}+a_{0}a_{1}d_{1}S^{*}}{\beta \eta_{1}k_{5}k_{6}}Q+\frac{\beta \eta_{1}\delta_{5}S^{*}+a_{0}a_{1}S^{*}}{\beta \eta_{1}k_{6}}H+\frac{a_{0}S^{*}}{\beta \eta_{1}}V. \end{array}$$

*Then*, *it is clear that*, *the functionLis nonnegative definite in* Ω *with respect to* $E_{0}$. *Calculating the time derivative of the function* *L* *along the solution of system* (2.2), *after lengthy computations*, *we get*

$$\frac{dL}{dt}=\frac{s\delta_{5}R_{c}}{\beta \eta_{1}}\left(2-\frac{S}{S^{*}}-\frac{S^{*}}{S}\right)+\frac{S^{*}\delta_{5}}{\eta_{1}}\left(I+A+U+\eta_{1}H+\frac{a_{0}}{\beta }V\right)\left(R_{c}-1\right).$$

*Thus*, *it follows that condition*$R_{c}\leq 1$*ensures*$\frac{dL\left(t\right)}{dt}\leq 0$, *for allS*, *E*, *I*, *A*, *U*, *H*, *V* ≥ 0, *with equality* $\left(\frac{dL\left(t\right)}{dt}=0\right)$ *if and only if* *S* = *S*∗, *E* = 0, *I* = 0, *A* = 0, *U* = 0, *H* = 0, *V* = 0. *Thus*, *L* *is a Lyapunov function on* Ω. *So*, *by LaSalle’s invariance Principle* [12, *Theorem* 5.3.1], *it follows that*

(4.5) $$\lim_{t\to \infty }\left(S,E,A,I,U,Q,H,V\right)=\left(\frac{s}{\mu },0,0,0,0,0,0,0\right).$$

*Let*K=(A,I,U,Q,H). *Then from*(4.5), *one has*${\mathrm{lim sup}\;}_{t\to \infty }K=0$. *This implies that for a sufficiently smallε* > 0 *there exist constants* *M*_*i*_ > 0, *i* = 1, …, 5 *such that* ${\mathrm{lim sup}\;}_{t\to \infty }K\leq \varepsilon$, *for all* *t* > *M*_*i*_, *i* = 1, …, 5. *Thus*, *from the eighth equation of system* (2.2), *it follows that*, *for* *t* > max_*i*∈{1,__…,5}_*M*_*i*_,

(4.6) $$R^{\infty }={\mathrm{lim sup}}_{t\to \infty }R\leq \frac{\rho \varepsilon +\nu \varepsilon +\gamma \varepsilon +\alpha \varepsilon +r\varepsilon }{\mu },$$

so that, *by letting* *ε* → 0 *in* (4.6), *we get*

(4.7) $$R^{\infty }={\mathrm{lim sup}}_{t\to \infty }R\leq 0.$$

*Also from*(4.5), *one has*${\mathrm{lim inf}\;}_{t\to \infty }K=0$. *Thus*, *by using a similar argument as above*, *it can be shown that*

(4.8) $$R_{\infty }={\mathrm{lim inf}}_{t\to \infty }R\geq 0.$$

*It then follows from*(4.7), (4.8)*that*

$$R_{\infty }\geq 0\geq R^{\infty }.$$

*This infers that*

(4.9) $$\lim_{t\to \infty }R\left(t\right)=0.$$

*Thus we have from*(4.5), (4.9)*that*,

$$\lim_{t\to \infty }\left(S,E,A,I,U,Q,H,V,R\right)=\left(\frac{s}{\mu },0,0,0,0,0,0,0,0\right)$$

.*Moreover*, Ω *is an invariant and attracting set of* $R_{+}^{9}$. *It follows that the largest compact invariant subset in* $\left\{\left(S,E,A,I,U,Q,H,V,R\right)\in \Omega :\;\frac{dL}{dt}=0\right\}$ *is the singleton* $\left\{E_{0}\right\}$. *So*, *by LaSalle’s invariance Principle* [12, *Theorem* 5.3.1], *it follows that every solution of system* (2.2), *with initial conditions in* $R_{+}^{9}$, *approaches the DFE*, $E_{0}$, *as* *t* → *∞* *whenever* $R_{c}\leq 1$. *This completes the proof*. □

Remark 4.4 *We note that the following Lyapunov function could also be used to prove Theorem* 4.3

(4.10) $$\begin{array}{lll} & & \tilde{M}\left(t\right)=\frac{\delta_{5}S^{*}}{\beta \eta_{1}}\left(\frac{S}{S^{*}}-1-\ln\frac{S}{S^{*}}\right)+\frac{\delta_{5}}{\beta \eta_{1}}E+\frac{k_{4}\beta \delta_{5}S^{*}+k_{4}a_{0}\omega_{1}S^{*}+a_{0}\theta \omega_{0}S^{*}+\beta \theta \delta_{5}S^{*}}{\beta \eta_{1}k_{2}k_{4}}A\\ & & \;\;+\frac{k_{6}\beta \delta_{5}S^{*}+a_{0}a_{1}d_{0}S^{*}+a_{0}\sigma k_{6}S^{*}+\beta \eta_{1}d_{0}\delta_{5}S^{*}}{\beta \eta_{1}k_{3}k_{6}}I+\frac{\beta \delta_{5}S^{*}+a_{0}\omega_{0}S^{*}}{\beta \eta_{1}k_{4}}U\\ & & \;\;+\frac{\beta \eta_{1}d_{1}\delta_{5}S^{*}+a_{0}a_{1}d_{1}S^{*}}{\beta \eta_{1}k_{5}k_{6}}Q+\frac{\beta \eta_{1}\delta_{5}S^{*}+a_{0}a_{1}S^{*}}{\beta \eta_{1}k_{6}}H+\frac{a_{0}S^{*}}{\beta \eta_{1}}V. \end{array}$$

*In this case*, *its derivative gives*

(4.11) $$\frac{d\tilde{M}\left(t\right)}{dt}=\frac{s\delta_{5}}{\beta \eta_{1}}\left(2-\frac{S}{S^{*}}-\frac{S^{*}}{S}\right)+\frac{k_{1}\delta_{5}}{\beta \eta_{1}}E\left(R_{c}-1\right).$$

Thus, *combining* (4.11) *and* (4.9) *also leads to the global asymptotical stability of the DFE,* $E_{0}$*.*

Theorem 4.3*implies that COVID-*19 *is eliminated from the population if the control reproduction number*, $R_{c}$, *of the model* (2.2) *is less than or equal to one*. *Thus*, Theorem 4.3 *means epidemiologically that the use of quarantine*, *hospitalization and the control of the amount of virus in the environment can lead to elimination of the COVID-*19 *if the mentioned controls can keep the threshold quantity*, $R_{c}$, *to a value less than or equal to unity*. *This implies that the condition* $R_{c}\leq 1$ *is necessary and sufficient for the elimination of COVID-*19. *Moreover*, *it follows from* Theorem 4.3 *that the longer infected individuals abide in the exposed class*, *the higher the likelihood of COVID-*19 *eradication from the population*.

### 4.3 Existence of the endemic equilibrium point (EEP)

Let $E^{*}=\;\left(S^{**},E^{**},A^{**},I^{**},U^{**},Q^{**},H^{**},V^{**},R^{**}\right)$ be any arbitrary equilibrium of system (2.2). In this section, we provide conditions for the existence of equilibria for which COVID-19 is endemic in the community, that is, at least one of the infected variables is non-zero. For this, consider the following associated force of infection for COVID-19 at endemic steady state

(4.12) $$\lambda_{s}^{**}=\beta \left(I^{**}+A^{**}+U^{**}+\eta_{1}H^{**}\right)+a_{0}V^{**}.$$

The endemic equilibrium point (EEP) of system (2.2) is obtained by setting the right hand side of the equations to zero; it is given in terms of $\lambda_{s}^{**}S^{**}$ as follows:

(4.13) $$\begin{array}{c} E^{**}=\frac{\lambda_{s}^{**}S^{**}}{k_{1}},\;A^{**}=\frac{k\lambda_{s}^{**}S^{**}}{k_{1}k_{2}},\;I^{**}=\frac{\eta \lambda_{s}^{**}S^{**}}{k_{1}k_{3}},\;U^{**}=N_{1}\lambda_{s}^{**}S^{**},\\ Q^{**}=\frac{\epsilon \lambda_{s}^{**}S^{**}}{k_{1}k_{5}},\;H^{**}=N_{2}\lambda_{s}^{**}S^{**},\;V^{**}=N_{3}\lambda_{s}^{**}S^{**},\;R^{**}=N_{4}\lambda_{s}^{**}S^{**}, \end{array}$$

where.

$S^{**}=\frac{s}{\lambda_{s}^{**}+\mu },\;N_{1}=\frac{b}{k_{1}k_{4}}+\frac{k\theta }{k_{1}k_{2}k_{4}}$, $N_{2}=\frac{d_{0}\eta }{k_{1}k_{3}k_{6}}+\frac{d_{1}\epsilon }{k_{1}k_{5}k_{6}}$, $N_{3}=\frac{\sigma \eta }{k_{1}k_{3}\delta_{5}}+\frac{a_{1}d_{0}\eta }{k_{1}k_{3}k_{6}\delta_{5}}+\frac{a_{1}d_{1}\epsilon }{k_{1}k_{5}k_{6}\delta_{5}}+\frac{k\omega_{1}}{k_{1}k_{2}\delta_{5}}+\frac{b\omega_{0}}{k_{1}k_{4}\delta_{5}}+\frac{k\theta \omega_{0}}{k_{1}k_{2}k_{4}\delta_{5}}$, $N_{4}=\frac{\rho \eta }{k_{1}k_{3}\mu }+\frac{b\nu }{k_{1}k_{4}\mu }+\frac{k\nu \theta }{k_{2}k_{2}k_{4}\mu }+\frac{k\gamma }{k_{1}k_{2}\mu }+\frac{\epsilon \alpha }{k_{1}k_{5}\mu }+\frac{rd_{0}\eta }{k_{1}k_{3}k_{6}\mu }+\frac{rd_{1}\epsilon }{k_{1}k_{5}k_{6}\mu }$.

Inserting the expressions of (4.13), except *R*∗∗, into (4.12), gives

(4.14) $$\begin{array}{c} \lambda_{s}^{**}=\lambda_{s}^{**}S^{**}\left[\frac{\beta \eta }{k_{1}k_{3}}+\frac{\beta k}{k_{1}k_{2}}+\beta N_{1}+\beta \eta_{1}N_{2}+a_{0}N_{3}\right]. \end{array}$$

Using the expression of *S*∗∗, equation (4.14) becomes

(4.15) $$\begin{array}{c} \lambda_{s}^{**}\lambda_{s}^{**}+\mu \lambda_{s}^{**}=s\lambda_{s}^{**}\left[\frac{\beta \eta }{k_{1}k_{3}}+\frac{\beta k}{k_{1}k_{2}}+\beta N_{1}+\beta \eta_{1}N_{2}+a_{0}N_{3}\right]. \end{array}$$

As mentioned above, we have $\lambda_{s}^{**}\neq 0$. Dividing each term in (4.15) by $\lambda_{s}^{**}$, we obtain

(4.16) $$1+\frac{1}{\mu }\lambda_{s}^{**}=\frac{\beta \eta s}{\mu k_{1}k_{3}}+\frac{\beta ks}{\mu k_{1}k_{2}}+\frac{\beta s}{\mu }N_{1}+\frac{\beta \eta_{1}s}{\mu }N_{2}+\frac{a_{0}s}{\mu }N_{3}.$$

It is worth noting that

$$\begin{array}{lll} & & 1+\frac{1}{\mu }\lambda_{s}^{**}=\frac{\beta ks}{\mu k_{1}k_{2}}+\frac{\beta \eta s}{\mu k_{1}k_{3}}+\frac{\beta bs}{\mu k_{1}k_{4}}+\frac{\beta \theta ks}{\mu k_{1}k_{2}k_{4}}+\frac{\beta \eta_{1}\eta d_{0}s}{\mu k_{1}k_{3}k_{6}}+\frac{\beta \eta_{1}\epsilon d_{1}s}{\mu k_{1}k_{5}k_{6}}+\frac{k\theta a_{0}\omega_{0}s}{\mu k_{1}k_{2}k_{4}\delta_{5}}\\ & & \;\;\;+\frac{ka_{0}\omega_{1}s}{\mu k_{1}k_{2}\delta_{5}}+\frac{\eta d_{0}a_{0}a_{1}s}{\mu k_{1}k_{3}k_{6}\delta_{5}}+\frac{\eta \sigma a_{0}s}{\mu k_{1}k_{3}\delta_{5}}+\frac{ba_{0}\omega_{0}s}{\mu k_{1}k_{4}\delta_{5}}+\frac{\epsilon d_{1}a_{0}a_{1}s}{\mu k_{1}k_{5}k_{6}\delta_{5}},\\ & & \;\;\;=R_{c}. \end{array}$$

Thus,

(4.17) $$\lambda_{s}^{**}=\mu \left(R_{c}-1\right)>0,\;\text{if}\;R_{c}>1.$$

Hence, each coordinate of the EEP $E^{*}$ is obtained by introducing the unique value of $\lambda_{s}^{**}$ provided in (4.17) into the different expressions in (4.13). Summarizing the above discussion on the EEP $E^{*}$, we obtain the following result.

Lemma 4.5 *If*$R_{c}>1$, *then system* (2.2) *admits in* $\overset{{}^{\circ}}{\Omega }$ *a unique positive endemic equilibrium*, $E^{*}$.

### 4.4 Local stability of the endemic equilibrium point

This section is devoted to the local stability of the unique endemic equilibrium point guaranteed by Lemma 4.2 whenever $R_{c}>1$. To do this, we follow the method developed in (Hethcote and Thieme, 1985) that takes its essence from the technique proposed by Krasnoselskii (Krasnoselskii, 1964).

We have the following result.

Theorem 4.6 *If*$R_{c}>1$, *then the unique endemic equilibrium point*, $E^{*}$, *of system* (2.2) *is locally asymptotically stable*.

proof. *First of all*, *note that the total population* *N* *is asymptotically constant*, *that is* *N* → *N*∗ *as* *t* → *∞*. *Thus*, *the proof of* Theorem 4.6 *is established by using a reduced system of* (2.2), *which is obtained by considering only the components* *E*, *A*, *I*, *U*, *Q*, *H*, *V*, *R*. *Thus*, *we can set* *N* = *N*∗, *for large* *t*, *so that the unique endemic equilibrium point*, $E^{*}$, *of the system* (2.2) *becomes* $E_{1}^{*}=E^{*}{|}_{N=N^{*}}$. *This eliminates the equation for* *S* *from this part of the analysis through the substitution* *S* = *N*∗ − (*E* + *A* + *I* + *U* + *Q* + *H* + *R*), *in which case*, *system* (2.2) *is reduced to*

(4.18) $$\left\{\begin{array}{l} \frac{dE}{dt}=\left(\beta \left(I+A+U+\eta_{1}H\right)+a_{0}V\right)\left(N^{*}-E-A-I-U-Q-H-R\right)-\left(\mu +k+\epsilon +b+\eta \right)E,\;\\ \frac{dA}{dt}=kE-\left(\mu +\delta_{4}+\gamma +\theta \right)A,\;\\ \frac{dI}{dt}=\eta E-\left(\mu +\delta_{1}+\rho +d_{0}\right)I,\;\\ \frac{dU}{dt}=bE+\theta A-\left(\mu +\delta_{3}+\nu \right)U,\;\\ \frac{dQ}{dt}=\epsilon E-\left(\mu +\alpha +d_{1}\right)Q,\;\\ \frac{dH}{dt}=d_{0}I+d_{1}Q-\left(\mu +\delta_{2}+r\right)H,\;\\ \frac{dV}{dt}=\sigma I+a_{1}H+\omega_{1}A+\omega_{0}U-\delta_{5}V,\;\\ \frac{dR}{dt}=\rho I+\nu U+\gamma A+\alpha Q+rH-\mu R.\; \end{array}\right)$$

*Now*, *linearizing system* (4.18) *at the endemic equilibrium point*, $E_{1}^{*}$, *yields*

(4.19) $$\left\{\begin{array}{l} \frac{dE}{dt}=\left[-e_{1}-\left(\mu +k+\epsilon +b+\eta \right)\right]E+\left(e_{2}-e_{1}\right)A+\left(e_{2}-e_{1}\right)I+\left(e_{2}-e_{1}\right)U-e_{1}Q+\left(\eta_{1}e_{2}-e_{1}\right)H+\frac{a_{0}e_{2}}{\beta }V-e_{1}R,\;\\ \frac{dA}{dt}=kE-\left(\mu +\delta_{4}+\gamma +\theta \right)A,\;\\ \frac{dI}{dt}=\eta E-\left(\mu +\delta_{1}+\rho +d_{0}\right)I,\;\\ \frac{dU}{dt}=bE+\theta A-\left(\mu +\delta_{3}+\nu \right)U,\;\\ \frac{dQ}{dt}=\epsilon E-\left(\mu +\alpha +d_{1}\right)Q,\;\\ \frac{dH}{dt}=d_{0}I+d_{1}Q-\left(\mu +\delta_{2}+r\right)H,\;\\ \frac{dV}{dt}=\sigma I+a_{1}H+\omega_{1}A+\omega_{0}U-\delta_{5}V,\;\\ \frac{dR}{dt}=\rho I+\nu U+\gamma A+\alpha Q+rH-\mu R,\; \end{array}\right)$$

*where*

$$e_{1}=\mu \left(R_{c}-1\right)\;\text{and}\;e_{2}=\frac{\beta s}{\mu R_{c}}.$$

*Thus*, *the Jacobian matrix of this linearized system* (4.19), *evaluated at* $E_{1}^{*}$, *is*

$$J_{1}\left(E_{1}^{*}\right)=\left(\begin{array}{cccccccc} -e_{1}-k_{1} & e_{2}-e_{1} & e_{2}-e_{1} & e_{2}-e_{1} & -e_{1} & \eta_{1}e_{2}-e_{1} & \frac{a_{0}e_{2}}{\beta } & -e_{1}\\ k & -k_{2} & 0 & 0 & 0 & 0 & 0 & 0\\ \eta & 0 & -k_{3} & 0 & 0 & 0 & 0 & 0\\ b & \theta & 0 & -k_{4} & 0 & 0 & 0 & 0\\ \epsilon & 0 & 0 & 0 & -k_{5} & 0 & 0 & 0\\ 0 & 0 & d_{0} & 0 & d_{1} & -k_{6} & 0 & 0\\ 0 & \omega_{1} & \sigma & \omega_{0} & 0 & a_{1} & -\delta_{5} & 0\\ 0 & \gamma & \rho & \nu & \alpha & r & 0 & -\mu \end{array}\right).$$

*Now*, *following the method developed in* (Hethcote and Thieme, 1985), *we assume that the linearized system* (4.19) *has solution of the form*

(4.20) $$Z\left(t\right)=Z_{0}e^{wt},$$

*with* *w* *and the components of* **Z**_0_ = (*Z*_1_, *Z*_2_, *Z*_3_, *Z*_4_, *Z*_5_, *Z*_6_, *Z*_7_, *Z*_8_) *in* C. *Substituting a solution of the form* (4.20) *into the linearized system* (4.19) *of the endemic equilibrium* $E_{1}^{*}$ *yields the following system of linear equations*

(4.21) $$\left\{\begin{array}{l} wZ_{1}=\left[-e_{1}-k_{1}\right]Z_{1}+\left(e_{2}-e_{1}\right)Z_{2}+\left(e_{2}-e_{1}\right)Z_{3}+\left(e_{2}-e_{1}\right)Z_{4}-e_{1}Z_{5}+\left(\eta_{1}e_{2}-e_{1}\right)Z_{6}+\frac{a_{0}e_{2}}{\beta }Z_{7}-e_{1}Z_{8},\;\\ wZ_{2}=kZ_{1}-k_{2}Z_{2},\;\\ wZ_{3}=\eta Z_{1}-k_{3}Z_{3},\;\\ wZ_{4}=bZ_{1}+\theta Z_{2}-k_{4}Z_{4},\;\\ wZ_{5}=\epsilon Z_{1}-k_{5}Z_{5},\;\\ wZ_{6}=d_{0}Z_{3}+d_{1}Z_{5}-k_{6}Z_{6},\;\\ wZ_{7}=\omega_{1}Z_{2}+\sigma Z_{3}+\omega_{0}Z_{4}+a_{1}Z_{6}-\delta_{5}Z_{7},\;\\ wZ_{8}=\gamma Z_{2}+\rho Z_{3}+\nu Z_{4}+\alpha Z_{5}+rZ_{7}-\mu Z_{8},\; \end{array}\right)$$

*where* *k*_*i*_, *i* = 1, …, 6, *are given in* (4.2).

*Now*, *by solving the second*, *third and fifth equations of*(4.21)*forZ*_2_, *Z*_3_*andZ*_5_, *and substituting the results into the other equations*, *we obtain the following system*:

(4.22) $$\left\{\begin{array}{l} ll\left[1+F_{1}\left(w\right)\right]Z_{1}={\left(GZ\right)}_{1},\;\left[1+F_{2}\left(w\right)\right]Z_{2}={\left(GZ\right)}_{2},\;\left[1+F_{3}\left(w\right)\right]Z_{3}={\left(GZ\right)}_{3},\;\left[1+F_{4}\left(w\right)\right]Z_{4}={\left(GZ\right)}_{4},\;\\ \left[1+F_{5}\left(w\right)\right]Z_{5}={\left(GZ\right)}_{5},\;\left[1+F_{6}\left(w\right)\right]Z_{6}={\left(GZ\right)}_{6},\;\left[1+F_{7}\left(w\right)\right]Z_{7}={\left(GZ\right)}_{7},\;\left[1+F_{8}\left(w\right)\right]Z_{8}={\left(GZ\right)}_{8},\; \end{array}\right)$$

*where*$F_{1}\left(w\right)=\frac{w}{k_{1}}+\frac{e_{1}}{k_{1}}\left[1+\frac{k}{w+k_{2}}+\frac{\eta }{w+k_{3}}+\frac{\epsilon }{w+k_{5}}\right]+\frac{e_{1}}{k_{1}\left(w+k_{4}\right)}\left[b+\frac{k\theta }{w+k_{2}}\right]$$+\frac{e_{1}}{k_{1}\left(w+k_{6}\right)}\left[\frac{d_{0}\eta }{w+k_{3}}+\frac{\epsilon d_{1}}{w+k_{5}}\right]+\frac{e_{1}}{k_{1}\left(w+\mu \right)}\left\{\frac{k\gamma }{w+k_{2}}+\frac{\rho \eta }{w+k_{3}}+\frac{\nu }{w+k_{4}}\left[b+\frac{k\theta }{w+k_{2}}\right]\right\}$$+\frac{e_{1}}{k_{1}\left(w+\mu \right)}\left\{\frac{\epsilon \alpha }{w+k_{5}}+\frac{r}{w+\delta_{5}}\left[\frac{k\omega_{1}}{w+k_{2}}+\frac{\sigma \eta }{w+k_{3}}+\frac{\epsilon a_{1}}{w+k_{5}}+\frac{\omega_{0}}{w+k_{4}}\left(b+\frac{k\theta }{w+k_{2}}\right)\right]\right\}$, $F_{2}\left(w\right)=\frac{w}{k_{2}},\;F_{3}\left(w\right)=\frac{w}{k_{3}},\;F_{4}\left(w\right)=\frac{w}{k_{4}},\;F_{5}\left(w\right)=\frac{w}{k_{5}},\;F_{6}\left(w\right)=\frac{w}{k_{6}},\;F_{7}\left(w\right)=\frac{w}{\delta_{5}},\;F_{8}\left(w\right)=\frac{w}{\mu }$

and

$$G=\left(\begin{array}{cccccccc} 0 & \frac{e_{2}}{k_{1}} & \frac{e_{2}}{k_{1}} & \frac{e_{2}}{k_{1}} & 0 & \frac{\eta_{1}e_{2}}{k_{1}} & \frac{a_{0}e_{2}}{\beta k_{1}} & 0\\ \frac{k}{k_{2}} & 0 & 0 & 0 & 0 & 0 & 0 & 0\\ \frac{\eta }{k_{3}} & 0 & 0 & 0 & 0 & 0 & 0 & 0\\ \frac{b}{k_{4}} & \frac{\theta }{k_{4}} & 0 & 0 & 0 & 0 & 0 & 0\\ \frac{\epsilon }{k_{5}} & 0 & 0 & 0 & 0 & 0 & 0 & 0\\ 0 & 0 & \frac{d_{0}}{k_{6}} & 0 & \frac{d_{1}}{k_{6}} & 0 & 0 & 0\\ 0 & \frac{\omega_{1}}{\delta_{5}} & \frac{\sigma }{\delta_{5}} & \frac{\omega_{0}}{\delta_{5}} & 0 & \frac{a_{1}}{\delta_{5}} & 0 & 0\\ 0 & \frac{\gamma }{\mu } & \frac{\rho }{\mu } & \frac{\nu }{\mu } & \frac{\alpha }{\mu } & \frac{r}{\mu } & 0 & 0 \end{array}\right).$$

*Note that*, *the non-zero entries of the matrixGare positive*, *and the equilibrium*$E_{1}^{*}=\;\left(E^{**},A^{**},I^{**},U^{**},Q^{**},H^{**},V^{**},R^{**}\right)$*satisfies*$E_{1}^{*}=GE_{1}^{*}$. *Here* (*GZ*)_*i*_, *i* = 1, …, 8, *denotes the ith component of the vector matrix* *GZ*. *Since the components of* $E_{1}^{*}$ *are all positive*, *then if* **Z** *represents any solution of system* (4.22), *there is a minimal positive real number* *c*_0_ (*see* (Esteva et al., 2009; Esteva and Vargas, 2000; Safi and Gumel, 2010) *and the references therein*) *such that*

(4.23) $$|Z|\leq c_{0}E_{1}^{*},$$

*where* |**Z**| = (|*Z*_1_|, |*Z*_2_|, |*Z*_3_|, |*Z*_4_|, |*Z*_5_|, |*Z*_6_|, |*Z*_7_|, |*Z*_8_|), *and* |*z*| *denotes the modulus of the complex number* *z*. *In fact*, *the goal is to prove that* Re *w* < 0. *This is done by contradiction*. *To do so*, *we assume that* Re *w* ≥ 0.

*First*, *we assume thatw* = 0.

*This case directly implies that*(4.21)*is a homogeneous linear system in the variablesZ*_*i*_, *i* = 1, …, 5.

*So*, *computing the determinant of this system yields*

(4.24) $$\begin{array}{lll} & & \Delta =B+\left[1-\frac{\beta s}{\mu R_{c}}\left(\frac{k}{k_{1}k_{2}}+\frac{\eta }{k_{1}k_{3}}+\frac{b}{k_{1}k_{4}}+\frac{\theta k}{k_{1}k_{2}k_{4}}+\frac{\eta_{1}\eta d_{0}}{k_{1}k_{3}k_{6}}+\frac{\eta_{1}\epsilon d_{1}}{k_{1}k_{5}k_{6}}+\frac{k\theta a_{0}\omega_{0}}{\beta k_{1}k_{2}k_{4}\delta_{5}}\right)\right)\\ & & \;\;\left(\left(+\frac{ka_{0}\omega_{1}}{\beta k_{1}k_{2}\delta_{5}}+\frac{\eta d_{0}a_{0}a_{1}}{\beta k_{1}k_{3}k_{6}\delta_{5}}+\frac{\eta \sigma a_{0}}{\beta k_{1}k_{3}\delta_{5}}+\frac{ba_{0}\omega_{0}}{\beta k_{1}k_{4}\delta_{5}}+\frac{\epsilon d_{1}a_{0}a_{1}}{\beta k_{1}k_{5}k_{6}\delta_{5}}\right)\right]\mu k_{1}k_{2}k_{3}k_{4}k_{5}k_{6}\delta_{5},\\ & & \;=B+\left(1-\frac{R_{c}}{R_{c}}\right)\mu k_{1}k_{2}k_{3}k_{4}k_{5}k_{6}\delta_{5},\\ & & \;=B, \end{array}$$

*where*

$$\begin{array}{lll} & & B=\epsilon \mu d_{1}k_{2}k_{3}k_{4}\delta_{5}e_{1}+\epsilon rd_{1}k_{2}k_{3}k_{4}\delta_{5}e_{1}+\epsilon \mu k_{2}k_{3}k_{4}k_{6}\delta_{5}e_{1}+\epsilon \alpha k_{2}k_{3}k_{4}k_{6}\delta_{5}e_{1}\\ & & \;+\mu k_{2}k_{3}k_{4}k_{5}k_{6}\delta_{5}e_{1}+\mu \theta kk_{3}k_{5}k_{6}\delta_{5}e_{1}+k\theta \nu k_{3}k_{5}k_{6}\delta_{5}e_{1}+\mu kk_{3}k_{4}k_{5}k_{6}\delta_{5}e_{1}\\ & & \;+\gamma kk_{3}k_{4}k_{5}k_{6}\delta_{5}e_{1}+\mu bk_{2}k_{3}k_{5}k_{6}\delta_{5}e_{1}+b\nu k_{2}k_{3}k_{5}k_{6}\delta_{5}e_{1}+\mu d_{0}\eta k_{2}k_{4}k_{5}\delta_{5}e_{1}\\ & & \;+rd_{0}\eta k_{2}k_{4}k_{5}\delta_{5}e_{1}+\mu \eta k_{2}k_{4}k_{5}k_{6}\delta_{5}e_{1}+\eta \rho k_{2}k_{4}k_{5}k_{6}\delta_{5}e_{1}>0. \end{array}$$

*Thus*, *one hasΔ* = *B* > 0. *Accordingly*, *system* (4.21) *has the vanishing solution* **Z** = 0, *which corresponds to the DFE*, $E_{0}$, *given in* (4.1).

*Now*, *we evaluate the second casew* ≠ 0.

*Since we have assumed that* Re *w* > 0, *then*, *it follows clearly that* |1 + *F*_*i*_(*w*)| > 1, *for all* *i* = 1, …, 8. *Define* *F*(*w*) = min_*i*∈{1, …,8}_|1 + *F*_*i*_(*w*)|. *Thus*, *F*(*w*) > 1, *and then* $\frac{c_{0}}{F\left(w\right)}<c_{0}$. *Note that* *c*_0_ *is a minimal positive real number such that* $|Z|\leq c_{0}E_{1}^{*}$. *Hence*, *it follows from the minimality of* *c*_0_ *that*

(4.25) $$|Z|>\frac{c_{0}}{F\left(w\right)}E_{1}^{*}.$$

*Now*, *by taking the norm on left and right sides of the third equation in*(4.22), *and using the fact thatGis a non-negative matrix*, *we get*

(4.26) $$F\left(w\right)|Z_{3}|\leq |1+F_{3}\left(w\right)\|Z_{3}|=|{\left(GZ\right)}_{3}|\leq G|Z_{3}|\leq c_{0}G{\left(E_{1}^{*}\right)}_{3}=c_{0}I^{**}.$$

*From*(4.26), *we obtain*$|Z_{3}|\leq \frac{c_{0}}{F\left(w\right)}I^{**}$. *This contradicts* (4.25). *Thus*, Re *w* < 0, *that is*, *all eigenvalues of the characteristic equation associated with the linearized system* (4.19) *around* $E_{1}^{*}$, *have negative real parts*. *Thus the unique EEP*, $E_{1}^{*}$, *is locally asymptotically stable whenever* $R_{c}>1$. *This completes the proof of* Theorem 4.6. □

Theorem 4.6*implies that*, *when*$R_{c}>1$, *COVID-*19 *will persist in the community if the initial sizes of the sub-populations*, *of the model*, *are in the basin of attraction of the EEP*$E_{1}^{*}=E^{*}{|}_{N=N^{*}}$.

### 4.5 Global stability of the endemic equilibrium

The following Theorem provides the global stability result for the endemic equilibrium point, $E^{*}$, of system (2.2).

Theorem 4.7 *The unique endemic equilibrium point of system* (2.2) *is globally asymptotically stable in* Ω∖Ω_0_ *whenever* $R_{c}>1$.

*Proof*. *Let* (*S*(*t*), *E*(*t*), *A*(*t*), *I*(*t*), *U*(*t*), *Q*(*t*), *H*(*t*), *V*(*t*), *R*(*t*)) *be any positive solution of system* (2.2) *in* Ω∖Ω_0_. *Define the following Lyapunov function*

$$\begin{array}{lll} & & M_{1}\left(t\right)=\frac{\delta_{5}S^{**}}{\beta \eta_{1}}\left(\frac{S}{S^{**}}-1-\ln\frac{S}{S^{**}}\right)+\frac{\delta_{5}}{\beta \eta_{1}}E^{**}\left(\frac{E}{E^{**}}-1-\ln\frac{E}{E^{**}}\right)\\ & & \;\;+\frac{k_{4}\beta \delta_{5}S^{**}+k_{4}a_{0}\omega_{1}S^{**}+a_{0}\theta \omega_{0}S^{**}+\beta \theta \delta_{5}S^{**}}{\beta \eta_{1}k_{2}k_{4}}A^{**}\left(\frac{A}{A^{**}}-1-\ln\frac{A}{A^{**}}\right)\\ & & \;\;+\frac{k_{6}\beta \delta_{5}S^{**}+a_{0}a_{1}d_{0}S^{**}+a_{0}\sigma k_{6}S^{**}+\beta \eta_{1}d_{0}\delta_{5}S^{**}}{\beta \eta_{1}k_{3}k_{6}}I^{**}\left(\frac{I}{I^{**}}-1-\ln\frac{I}{I^{**}}\right)\\ & & \;\;+\frac{\beta \delta_{5}S^{**}+a_{0}\omega_{0}S^{**}}{\beta \eta_{1}k_{4}}U^{**}\left(\frac{U}{U^{**}}-1-\ln\frac{U}{U^{**}}\right)+\frac{\beta \eta_{1}d_{1}\delta_{5}S^{**}+a_{0}a_{1}d_{1}S^{**}}{\beta \eta_{1}k_{5}k_{6}}Q^{**}\left(\frac{Q}{Q^{**}}-1-\ln\frac{Q}{Q^{**}}\right)\\ & & \;\;+\frac{\beta \eta_{1}\delta_{5}S^{**}+a_{0}a_{1}S^{**}}{\beta \eta_{1}k_{6}}H^{**}\left(\frac{H}{H^{**}}-1-\ln\frac{H}{H^{**}}\right)+\frac{a_{0}S^{**}}{\beta \eta_{1}}V^{**}\left(\frac{V}{V^{**}}-1-\ln\frac{V}{V^{**}}\right). \end{array}$$

*Using the equilibrium conditions*, *after lengthy computations*, *the derivative of the above Lyapunov function computed along the solutions of system* (2.2) *is given below*:

$$\frac{dM_{1}\left(t\right)}{dt}=\frac{s\delta_{5}}{\beta \eta_{1}}\left(2-\frac{S}{S^{**}}-\frac{S^{**}}{S}\right)+\frac{S^{**}\delta_{5}}{\eta_{1}}I^{**}\left(3-\frac{S^{**}}{S}-\frac{SIE^{**}}{ES^{**}I^{**}}-\frac{EI^{**}}{IE^{**}}\right)+\frac{S^{**}\delta_{5}}{\eta_{1}}A^{**}\left(3-\frac{S^{**}}{S}-\frac{SAE^{**}}{ES^{**}A^{**}}-\frac{EA^{**}}{AE^{**}}\right)+\frac{S^{**}a_{0}\omega_{1}}{\beta \eta_{1}}A^{**}\left(4-\frac{S^{**}}{S}-\frac{EA^{**}}{AE^{**}}-\frac{AV^{**}}{VA^{**}}-\frac{SVE^{**}}{ES^{**}V^{**}}\right)+\frac{S^{**}a_{0}\omega_{0}\theta }{\beta \eta_{1}k_{4}}A^{**}\left(5-\frac{S^{**}}{S}-\frac{EA^{**}}{AE^{**}}-\frac{AU^{**}}{UA^{**}}-\frac{UV^{**}}{VU^{**}}-\frac{SVE^{**}}{ES^{**}V^{**}}\right)+\frac{S^{**}\theta \delta_{5}}{\eta_{1}k_{4}}A^{**}\left(4-\frac{S^{**}}{S}-\frac{EA^{**}}{AE^{**}}-\frac{AU^{**}}{UA^{**}}-\frac{SUE^{**}}{ES^{**}U^{**}}\right)+\frac{S^{**}b\delta_{5}}{\eta_{1}k_{4}}E^{**}\left(3-\frac{S^{**}}{S}-\frac{EU^{**}}{UE^{**}}-\frac{SUE^{**}}{ES^{**}V^{**}}\right)+\frac{S^{**}a_{0}\omega_{0}b}{\beta \eta_{1}k_{4}}E^{**}\left(4-\frac{S^{**}}{S}-\frac{EU^{**}}{UE^{**}}-\frac{UV^{**}}{VU^{**}}-\frac{SVE^{**}}{ES^{**}V^{**}}\right)+\frac{S^{**}a_{0}a_{1}d_{0}}{\beta \eta_{1}k_{6}}I^{**}\left(5-\frac{S^{**}}{S}-\frac{EI^{**}}{IE^{**}}-\frac{IH^{**}}{HI^{**}}-\frac{HV^{**}}{VH^{**}}-\frac{SVE^{**}}{ES^{**}V^{**}}\right)+\frac{S^{**}a_{0}\sigma }{\beta \eta_{1}}I^{**}\left(4-\frac{S^{**}}{S}-\frac{EI^{**}}{IE^{**}}-\frac{IV^{**}}{VI^{**}}-\frac{SVE^{**}}{ES^{**}V^{**}}\right)+\frac{S^{**}d_{0}\delta_{5}}{k_{6}}I^{**}\left(4-\frac{S^{**}}{S}-\frac{EI^{**}}{IE^{**}}-\frac{IH^{**}}{HI^{**}}-\frac{SHE^{**}}{ES^{**}H^{**}}\right)+\frac{S^{**}d_{1}\delta_{5}}{k_{6}}Q^{**}\left(4-\frac{S^{**}}{S}-\frac{EQ^{**}}{QE^{**}}-\frac{QH^{**}}{HQ^{**}}-\frac{SHE^{**}}{ES^{**}H^{**}}\right)+\frac{S^{**}a_{0}a_{1}d_{1}}{\beta \eta_{1}k_{6}}Q^{**}\left(5-\frac{S^{**}}{S}-\frac{EQ^{**}}{QE^{**}}-\frac{QH^{**}}{HQ^{**}}-\frac{HV^{**}}{VH^{**}}-\frac{SVE^{**}}{ES^{**}V^{**}}\right).$$

*Thus*, *by using the arithmetic-geometric means inequality and condition*$R_{c}>1$, *it follows that*$\frac{dM_{1}\left(t\right)}{dt}\leq 0$. *Moreover*, $\frac{dM_{1}\left(t\right)}{dt}=0$, *holds if and only ifS* = *S*∗∗, *E* = *E*∗∗, *A* = *A*∗∗, *I* = *I*∗∗, *U* = *U*∗∗, *Q* = *Q*∗∗, *H* = *H*∗∗, *V* = *V*∗∗. *Consequently*, $M_{1}$ *is a Lyapunov function on* Ω∖Ω_0_. *So*, *by LaSalle’s invariance principle* [12, *Theorem* 5.3.1], *it follows that*

(4.27) $$\lim_{t\to \infty }\left(S\left(t\right),E\left(t\right),A\left(t\right),I\left(t\right),U\left(t\right),Q\left(t\right),H\left(t\right),V\left(t\right)\right)=\left(S^{**},E^{**},A^{**},I^{**},U^{**},Q^{**},H^{**},V^{**}\right).$$

*Again*, *combining this with system* (2.2), *gives* lim_*t*→*∞*_*R*(*t*) = *R*∗∗ *as described in the proof of Theorem* 4.1. *Thus*, *every solution of the model*, *with initial condition in* Ω∖Ω_0_, *approaches the unique endemic equilibrium point of system* (2.2) *when* *t* *tends to* *∞* *for* $R_{c}>1$. *This completes the proof*. □

**Fig. 2:**
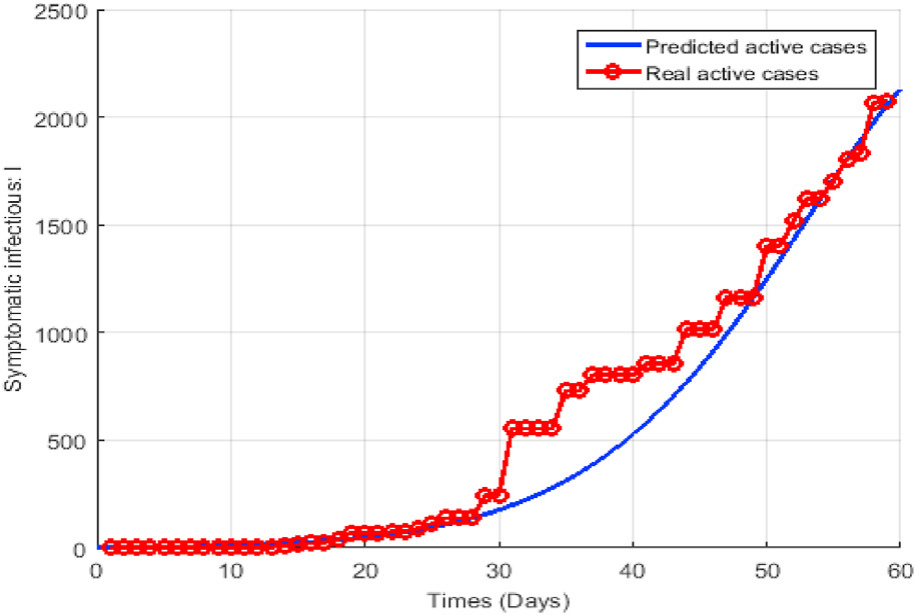
Fitted results from the model (2.2) using the parameter values from Table 1 except the following parameters: β = 1.55 × 10^−6^, η_1_ = 0.49, a_0_ = 10^−7^, μ = 1/59, d_1_ = 0.156986, and $R_{c}=1.2331>1$. Here, the red line indicates the real symptomatic infectious cases and the blue line indicates the predicted symptomatic infectious individuals.

In other words, Theorem 4.7 shows that COVID-19 will persist in the community whenever $R_{c}>1$. Furthermore, it follows from Theorem 4.7 that an imperfect follow-up of patients tested positive could lead to infection of many people in the community. Fig. 2 below shows a good fit for total actual symptomatic infectious individuals and those predicted by the model (2.2).

### 4.6 Sensitivity analysis with respect to quarantine an hospitalization

Here we analyze the threshold quantity $R_{c}$, around the parameters associated to the quarantine of exposed individuals (*ϵ*) and the hospitalization of individuals with COVID-19 symptoms (*d*_0_), in order to measure the effect of quarantine and hospitalization on the transmission dynamics of the disease. For this, we compute the partial derivative of $R_{c}$ with respect to the aforementioned parameters. First, computing the partial derivative of $R_{c}$ with respect to *ϵ*, we obtain

(4.28) $$\frac{\partial R_{c}}{\partial \epsilon }=\frac{\beta \delta_{5}s\left(d_{1}k_{01}k_{3}-\eta d_{0}k_{5}\right)\eta_{1}-s\left(B_{0}-d_{1}a_{0}a_{1}k_{01}k_{3}\right)}{\mu k_{1}^{2}k_{3}k_{5}k_{6}\delta_{5}},$$

where

$$\begin{array}{lll} & & B_{0}=\beta \eta k_{5}k_{6}\delta_{5}+\eta d_{0}a_{0}a_{1}k_{5}+\eta \sigma a_{0}k_{5}k_{6}+\frac{\beta k_{3}k_{5}k_{6}\delta_{5}k}{k_{2}}+\frac{\beta bk_{3}k_{5}k_{6}\delta_{5}}{k_{4}}\\ & & \;\;+\frac{\beta \theta kk_{3}k_{5}k_{6}\delta_{5}}{k_{2}k_{4}}+\frac{k\theta a_{0}\omega_{0}k_{3}k_{5}k_{6}}{k_{2}k_{4}}+\frac{ka_{0}\omega_{1}k_{3}k_{5}k_{6}}{k_{2}}+\frac{ba_{0}\omega_{0}k_{3}k_{5}k_{6}}{k_{4}}. \end{array}$$

It follows from (4.28) that

$$\frac{\partial R_{c}}{\partial \epsilon }<0\;\text{if and only if}\;\;\eta_{1}<\eta_{1\epsilon },$$

and

$$\frac{\partial R_{c}}{\partial \epsilon }>0\;\text{if and only if}\;\;\eta_{1}>\eta_{1\epsilon },$$

with

(4.29) $$0<\eta_{1\epsilon }=\frac{B_{0}-d_{1}a_{0}a_{1}k_{01}k_{3}}{\beta \delta_{5}\left(d_{1}k_{01}k_{3}-\eta d_{0}k_{5}\right)}.$$

This first evaluation implies that the quarantine of exposed individuals can reduce the control reproduction number, and COVID-19 will reduce burden if the relative infectiousness of hospitalized individuals, *η*_1_, does not exceed the threshold quantity *η*_1*ϵ*_. If *η*_1_ > *η*_1*ϵ*_, the use of quarantine of exposed individuals will increase the control reproduction number, and COVID-19 will increase burden. Thus, the use of quarantine is injurious to the population.

The above discussion is summed up in the following result.

Lemma 4.8 *The use of quarantine of the exposed individuals will have positive impact on the population if* η_1_ < η_1ϵ_, *and negative impact on the population whenever* η_1_ > η_1ϵ_.

*Similarly*, *the computation of the partial derivative of*$R_{c}$*with respectd*_0_, *gives*

(4.30) $$\frac{\partial R_{c}}{\partial d_{0}}=\frac{\beta \eta k_{03}\delta_{5}s\eta_{1}-s\eta \left(k_{6}\left(\sigma a_{0}+\beta \delta_{5}\right)-a_{0}a_{1}k_{03}\right)}{\mu k_{1}k_{3}^{2}k_{6}\delta_{5}},$$

*It follows from*(4.30)*that*

$$\frac{\partial R_{c}}{\partial d_{0}}<0\;\text{if and only if}\;\;\eta_{1}<\eta_{1d_{0}},$$

*and*

$$\frac{\partial R_{c}}{\partial d_{0}}>0\;\text{if and only if}\;\;\eta_{1}>\eta_{1d_{0}},$$

*with*

(4.31) $$0<\eta_{1d_{0}}=\frac{k_{6}\left(\sigma a_{0}+\beta \delta_{5}\right)-a_{0}a_{1}k_{03}}{\beta k_{03}\delta_{5}}.$$

*This last evaluation implies that*, *the hospitalization of individuals with COVID-*19 *symptoms will be beneficial to the population if the relative infectiousness of hospitalized individuals does not exceed the threshold quantity*$\eta_{1d_{0}}$, *and is not beneficial if*$\eta_{1}>\eta_{1d_{0}}$. *We have the following result*.

Lemma 4.9 *Hospitalization of individuals with COVID-19 symptoms will have positive impact on the population if*$\eta_{1}<\eta_{1d_{0}}$*, and negative impact on the population if*$\eta_{1}>\eta_{1d_{0}}$*.*

*Combining Lemma* 4.3 *and Lemma* 4.4, *we get the following result*.

Theorem 4.10 *The use of quarantine of exposed individual and hospitalization of individuals with COVID-19 symptoms will have*

a) *positive impact on the population if*$\eta_{1}<\min\left\{\eta_{1\epsilon },\eta_{1d_{0}}\right\}$*;*
b) *no impact on the population if*$\eta_{1}=\min\left\{\eta_{1\epsilon },\eta_{1d_{0}}\right\}$*;*
c) *negative impact on the population if*$\eta_{1}>\max\left\{\eta_{1\epsilon },\eta_{1d_{0}}\right\}$.

**Fig. 3:**
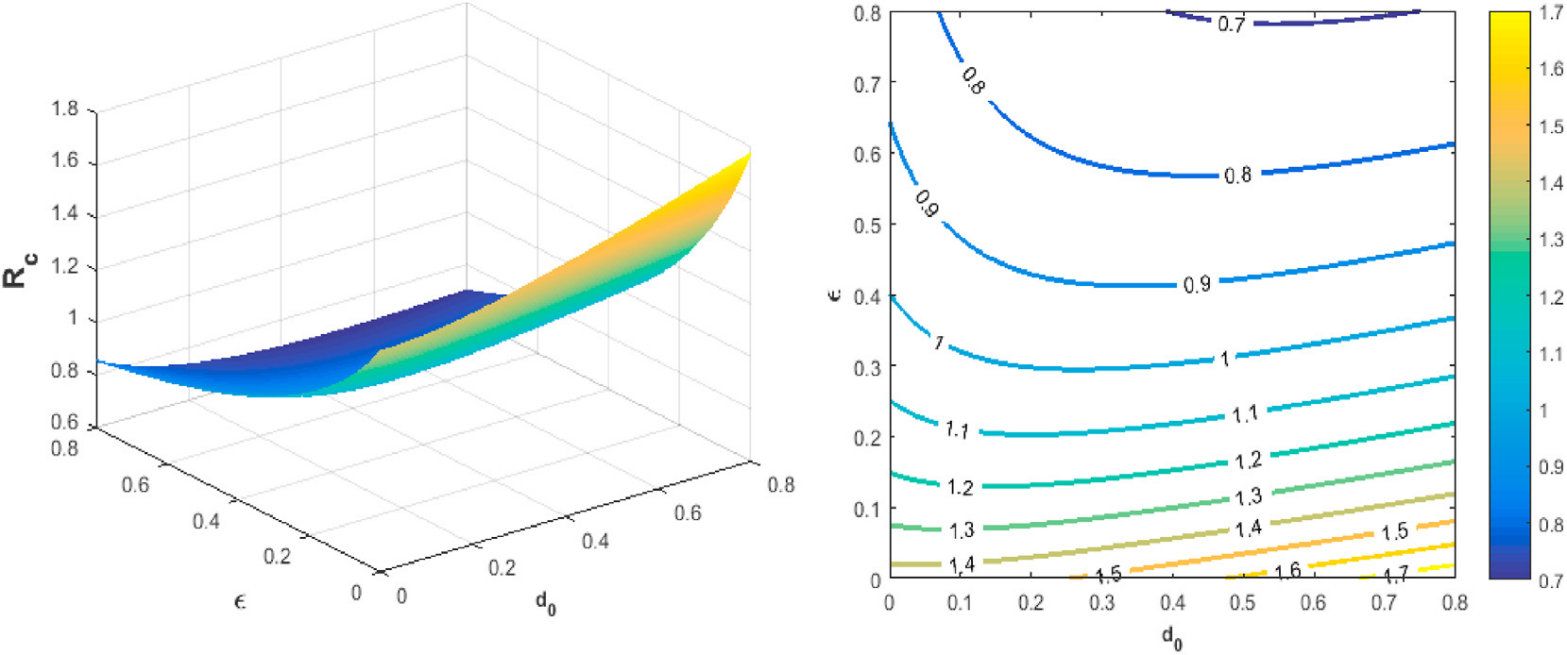
*Graph and contour plots of*$R_{c}$*as a function of quarantine rate of exposed individualsϵand hospitalization rate of symptomatic infectious individualsd*_0_.

*The first item of Theorem* 4.4 *means that the threshold quantity* $R_{c}$ *is a decreasing function of the quarantine and hospitalization parameters* *ϵ* *and* *d*_0_, *respectively*; *while the last item implies that* $R_{c}$ *is an increasing function of these parameters*. *The graph of* Fig. 3 *shows that the control reproduction number* $R_{c}$ *is a decreasing function of the quarantine rate* *ϵ* *and the hospitalization rate* *d*_0_. *This underscores the importance of the quarantine rate* *ϵ* *and the hospitalization rate* *d*_0_ *in controlling the COVID-*19 *disease in Cameroon*.

## 5 Optimal control problem

**Fig. 4:**
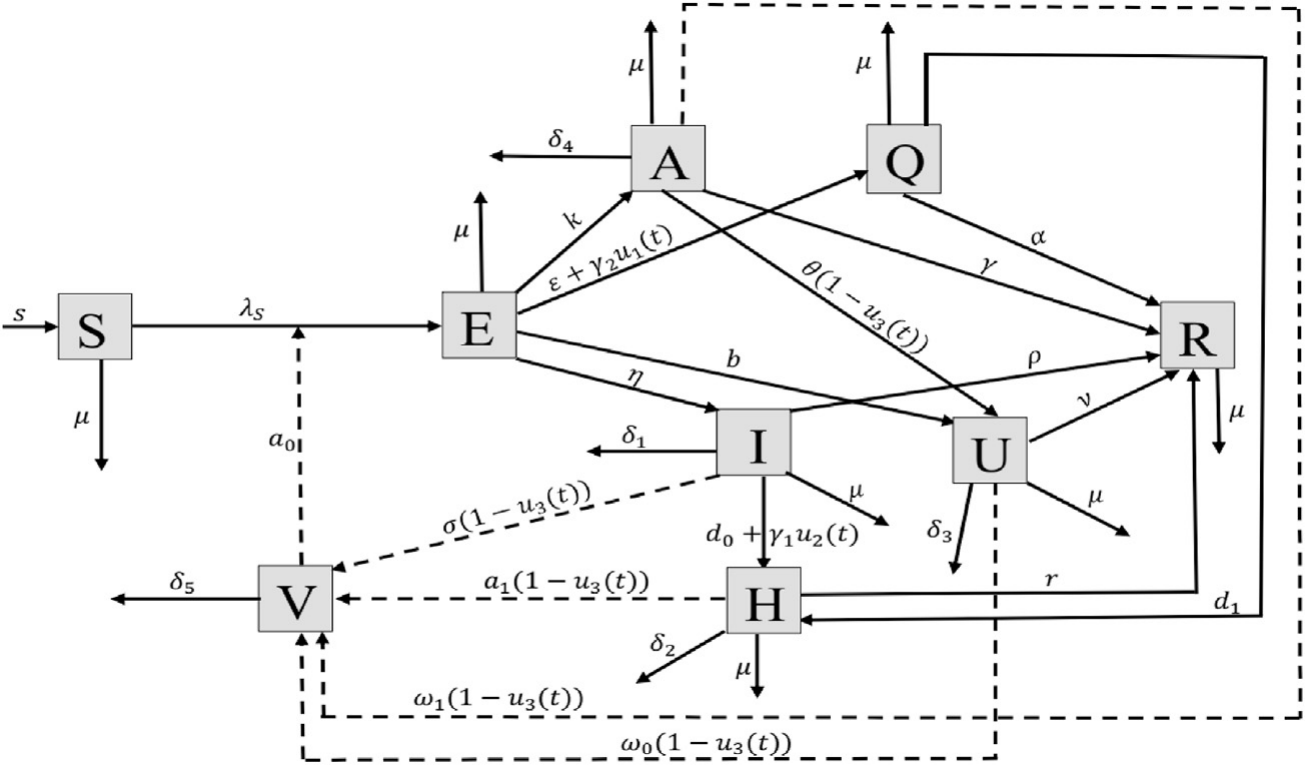
Flow diagram of the model with controls.

COVID-19 has not yet been controlled and is still ongoing. Thus, to expect that the disease can stop, we need to comply with barrier measures (such as the regular washing of hands, the use of hydro-alcoholic gel, wearing face masks, social distancing rules). In this Section, we propose and investigate an optimal control problem applied to COVID-19 dynamics described by system (2.2)that we extend by adding three control functions *u*_1_, *u*_2_ and *u*_3_. The control *u*_1_ denotes the quarantining rate of individuals who have been in contact with infected individuals and have accepted to be quarantined during a period of time (Yan et al., 2007). The term *γ*_2_*u*_1_ denotes the rate of mandatory quarantine. In this case, the parameter *ϵ* becomes the natural quarantined rate. Next, the control function *u*_2_, which measures the rate of tracing, testing and hospitalization of people with clinical symptoms, moves infectious individuals from their symptomatic class to hospitalized class, under an hospitalization program for special medical treatment at rate *γ*_1_, with the natural hospitalization rate *d*_0_. Thus, *u*_2_ decreases the evolution of symptomatic class to hospitalized class. The control *u*_3_ represents the global effort of educational campaigns. The term 1 − *u*_3_(*t*) is a decreasing factor that indicates the extent to which the production of unreported symptomatic individuals is blocked as a result of multiple educational campaigns. Furthermore, from the factor 1 − *u*_3_(*t*), through the aforementioned barrier measures, people in the community can significantly reduce the concentration of virus in the environment. The flow diagram of the model with controls which elucidates the transmission phases of COVID-19 is presented in Fig. 4.

From the flow diagram in Fig. 4, we propose the following nonlinear system with control:

(5.1) $$\left\{\begin{array}{l} \frac{dS}{dt}=s-\beta S\left(I+A+U+\eta_{1}H\right)-a_{0}SV-\mu S,\;\\ \frac{dE}{dt}=\beta S\left(I+A+U+\eta_{1}H\right)+a_{0}SV-\left(\mu +k+\epsilon +b+\eta +\gamma_{2}u_{1}\left(t\right)\right)E,\;\\ \frac{dA}{dt}=kE-\left(\mu +\delta_{4}+\gamma +\theta \left(1-u_{3}\left(t\right)\right)\right)A,\;\\ \frac{dI}{dt}=\eta E-\left(\mu +\delta_{1}+\rho +d_{0}+\gamma_{1}u_{2}\left(t\right)\right)I,\;\\ \frac{dU}{dt}=bE+\theta \left(1-u_{3}\left(t\right)\right)A-\left(\mu +\delta_{3}+\nu \right)U,\;\\ \frac{dQ}{dt}=\left(\epsilon +\gamma_{2}u_{1}\left(t\right)\right)E-\left(\mu +\alpha +d_{1}\right)Q,\;\\ \frac{dH}{dt}=\left(d_{0}+\gamma_{1}u_{2}\left(t\right)\right)I+d_{1}Q-\left(\mu +\delta_{2}+r\right)H,\;\\ \frac{dV}{dt}=\sigma \left(1-u_{3}\left(t\right)\right)I+a_{1}\left(1-u_{3}\left(t\right)\right)H+\omega_{1}\left(1-u_{3}\left(t\right)\right)A+\omega_{0}\left(1-u_{3}\left(t\right)\right)U-\delta_{5}V,\;\\ \frac{dR}{dt}=\rho I+\nu U+\gamma A+\alpha Q+rH-\mu R.\; \end{array}\right)$$

All the parameters and classes of system (5.1) are the same as in system (2.2). The optimal control problem associated to model (5.1) requires the minimization of *E*(*t*), *A*(*t*), *I*(*t*), *U*(*t*), *Q*(*t*), *H*(*t*) and *V*(*t*) as well as the cost of implementation of the interventions needed. Let *T* be a fixed terminal time. The objective functional which we seek to minimize is defined as in (Yan et al., 2007) as follows:

(5.2) $$J\left(u_{1}\left(t\right),u_{2}\left(t\right),u_{3}\left(t\right)\right)=\int_{0}^{T}\left[B_{1}E\left(t\right)+B_{2}A\left(t\right)+B_{3}I\left(t\right)+B_{4}U\left(t\right)+B_{5}Q\left(t\right)+B_{6}H\left(t\right)+B_{7}V\left(t\right)\right)\left(+\frac{1}{2}R_{1}u_{1}^{2}\left(t\right)+\frac{1}{2}R_{2}u_{2}^{2}\left(t\right)+\frac{1}{2}R_{3}u_{3}^{2}\left(t\right)\right]dt.$$

*B*_*i*_, *i* = 1, …, 7 represent the cost coefficients for *E*(*t*), *A*(*t*), *I*(*t*), *U*(*t*), *Q*(*t*), *H*(*t*) and *V*(*t*), respectively. *R*_1_, *R*_2_ and *R*_3_ are cost balancing coefficients associated with the hospitalized individuals in designated, susceptible quarantined individuals, and a strategy applied to the whole population.

The admissible controls set is defined as

$$F=\left\{\left(u_{1},u_{2},u_{3}\right):u_{i}\;\text{is measurable},0\leq u_{i}\left(t\right)\leq b_{i},0<b_{i}\leq 1,t\in \left[0,T\right],i=1,2,3\right\},$$

where *b*_*i*_, *i* = 1, 2, 3, are fixed positive constant which depend on the amount of resources available for the implementation of the control strategies. We need to determine the optimal control $\left(u_{1}^{*},u_{2}^{*},u_{3}^{*}\right)$ such that

$$J\left(u_{1}^{*},u_{2}^{*},u_{3}^{*}\right)=\min\left\{J\left(u_{1},u_{2},u_{3}\right):\left(u_{1},u_{2},u_{3}\right)\in F\right\}.$$

This is given in the following Theorem.

Theorem 5.1 *Consider the control problem with objective functional* (5.2) *and system* (5.1)*. Then, there exists an optimal control* $u^{*}=\left(u_{1}^{*},u_{2}^{*},u_{3}^{*}\right)\in F$ *such that*

$$J\left(u_{1}^{*},u_{2}^{*},u_{3}^{*}\right)=\min_{\left(u_{1},u_{2},u_{3}\right)\in F}J\left(u_{1},u_{2},u_{3}\right),$$

provided the following conditions are satisfied:

(a) *The class of all initial conditions with controlsu* = (*u*_1_, *u*_2_, *u*_3_) *in the set of admissible controls*, *with system* (5.1) *being satisfied*, *is not empty*.
(b) *The set of admissible controls*F*is convex and closed*.
(c) *The right-hand side of system* (5.1) *is continuous*, *bounded from above by a sum of the bounded control and the state*, *and can be written as a linear function of controls* (*u*_1_, *u*_2_, *u*_3_) *with coefficients depending on time and state*.
(d) *The integrand of the objective functional* (5.2) *is convex on* F *and bounded from below by* $-e_{0}+e_{1}\left(|u_{1}{|}^{2}+|u_{2}{|}^{2}+|u_{3}{|}^{2}\right)$, *where* *e*_0_ ≥ 0 *and* *e*_1_ > 0.

proof. *The proof is done by applying similar arguments as in the proof of Theorem* 4.1 *in* (Fleming and Rishel, 1975). □ *We now investigate the necessary conditions for the optimal control by using the Pontryagin’s maximum principle* (Pontryagin et al., 1962). *Define the Lagrangian for this control problem as in* (Joshi, 2002; Kirk, 2004):

$$\begin{array}{lll} & & L\left(S,E,A,I,U,Q,H,V,R,u_{1},u_{2},u_{3},\lambda_{1},\lambda_{2},\lambda_{3},\lambda_{4},\right)\\ & & \left(\;\lambda_{5},\lambda_{6},\lambda_{7},\lambda_{8},\lambda_{9},w_{11},w_{12},w_{21},w_{22},w_{31},w_{32}\right)\\ & & =\left[B_{1}E\left(t\right)+B_{2}A\left(t\right)+B_{3}I\left(t\right)+B_{4}U\left(t\right)+B_{5}Q\left(t\right)\right)\\ & & \;\left(+B_{6}H\left(t\right)+B_{7}V\left(t\right)+\frac{1}{2}R_{1}u_{1}^{2}+\frac{1}{2}R_{2}u_{2}^{2}+\frac{1}{2}R_{3}u_{3}^{2}\right]\\ & & \;+\lambda_{1}\left[s-\beta S\left(I\left(t\right)+A\left(t\right)+U\left(t\right)+\eta_{1}H\left(t\right)\right)-a_{0}S\left(t\right)V\left(t\right)-\mu S\left(t\right)\right]\\ & & \;+\lambda_{2}\left[\beta S\left(I\left(t\right)+A\left(t\right)+U\left(t\right)+\eta_{1}H\left(t\right)\right)+a_{0}S\left(t\right)V\left(t\right)\right)\\ & & \;\left(-\left(\mu +k+\epsilon +b+\eta +\gamma_{2}u_{1}\left(t\right)\right)E\left(t\right)\right]\\ & & \;+\lambda_{3}\left[kE\left(t\right)-\left(\mu +\delta_{4}+\gamma +\theta \left(1-u_{3}\left(t\right)\right)\right)A\left(t\right)\right]\\ & & \;+\lambda_{4}\left[\eta E\left(t\right)-\left(\mu +\delta_{1}+\rho +d_{0}+\gamma_{1}u_{2}\left(t\right)\right)I\left(t\right)\right]\\ & & \;+\lambda_{5}\left[bE\left(t\right)+\theta \left(1-u_{3}\left(t\right)\right)A\left(t\right)-\left(\mu +\delta_{3}+\nu \right)U\left(t\right)\right]\\ & & \;+\lambda_{6}\left[\left(\epsilon +\gamma_{2}u_{1}\left(t\right)\right)E\left(t\right)-\left(\mu +\alpha +d_{1}\right)Q\left(t\right)\right]\\ & & \;+\lambda_{7}\left[\left(d_{0}+\gamma_{1}u_{2}\left(t\right)\right)I\left(t\right)+d_{1}Q\left(t\right)-\left(\mu +\delta_{2}+r\right)H\left(t\right)\right]\\ & & \;+\lambda_{8}\left[\sigma \left(1-u_{3}\left(t\right)\right)I\left(t\right)+a_{1}\left(1-u_{3}\left(t\right)\right)H\left(t\right)+\omega_{1}\left(1-u_{3}\left(t\right)\right)A\left(t\right)\right)\\ & & \;\left(+\omega_{0}\left(1-u_{3}\left(t\right)\right)U\left(t\right)-\delta_{5}V\left(t\right)\right]\\ & & \;+\lambda_{9}\left[\rho I\left(t\right)+\nu U\left(t\right)+\gamma A\left(t\right)+\alpha Q\left(t\right)+rH\left(t\right)-\mu R\left(t\right)\right]\\ & & \;-w_{11}\left(t\right)\left(b_{1}-u_{1}\left(t\right)\right)-w_{12}\left(t\right)u_{1}\left(t\right)-w_{21}\left(t\right)\left(b_{2}-u_{2}\left(t\right)\right)\\ & & \;-w_{22}\left(t\right)u_{2}\left(t\right)-w_{31}\left(t\right)\left(b_{3}-u_{3}\left(t\right)\right)-w_{32}\left(t\right)u_{3}\left(t\right), \end{array}$$

*where* *w*_11_(*t*), *w*_12_(*t*), *w*_21_(*t*), *w*_22_(*t*), *w*_31_(*t*), *w*_32_(*t*) ≥ 0 *are penalty multipliers satisfying the following equations at* *u*∗:

$$\begin{array}{c} w_{11}\left(t\right)\left(b_{1}-u_{1}\left(t\right)\right)=0,\;w_{12}\left(t\right)u_{1}\left(t\right)=0,\\ w_{21}\left(t\right)\left(b_{2}-u_{2}\left(t\right)\right)=0,\;w_{22}\left(t\right)u_{2}\left(t\right)=0,\\ \;w_{31}\left(t\right)\left(b_{3}-u_{3}\left(t\right)\right)=0,\;w_{32}\left(t\right)u_{3}\left(t\right)=0. \end{array}$$

*Differentiating the above Lagrangian with respect to state variablesS*, *E*, *A*, *I*, *U*, *Q*, *H*, *V*, *andR*, *respectively*, *and applying Pontryagin’s maximum principle*, *the adjoint system reads*

(5.3) $$\begin{array}{lll} & & \lambda_{1}^{\prime }=-\frac{\partial L}{\partial S},\;\lambda_{2}^{\prime }=-\frac{\partial L}{\partial E},\;\lambda_{3}^{\prime }=-\frac{\partial L}{\partial A},\;\lambda_{4}^{\prime }=-\frac{\partial L}{\partial I},\;\lambda_{5}^{\prime }=-\frac{\partial L}{\partial U},\\ & & \lambda_{6}^{\prime }=-\frac{\partial L}{\partial Q},\;\lambda_{7}^{\prime }=-\frac{\partial L}{\partial H},\;\lambda_{8}^{\prime }=-\frac{\partial L}{\partial V},\;\lambda_{9}^{\prime }=-\frac{\partial L}{\partial R}, \end{array}$$

*and the transversality conditionsλ*_*i*_(*T*) = 0, *i* = 1, …, 9, *hold*. *Setting* $\frac{\partial L}{\partial u_{i}}=0$, *i* = 1, 2, 3, *the optimality conditions are given by*

$$\begin{array}{lll} & & \frac{\partial L}{\partial u_{1}}=R_{1}u_{1}-\lambda_{2}\gamma_{2}E+\lambda_{6}\gamma_{2}E+w_{11}\left(t\right)-w_{12}\left(t\right)=0,\\ & & \frac{\partial L}{\partial u_{2}}=R_{2}u_{2}-\lambda_{4}\gamma_{1}I+\lambda_{7}\gamma_{1}I+w_{21}\left(t\right)-w_{22}\left(t\right)=0,\\ & & \frac{\partial L}{\partial u_{3}}=R_{3}u_{3}+\lambda_{3}\theta A-\lambda_{5}\theta A-\lambda_{8}\sigma I-\lambda_{8}a_{1}H-\lambda_{8}\omega_{1}A\\ & & \;\;-\lambda_{8}\omega_{0}U+w_{31}\left(t\right)-w_{32}\left(t\right)=0. \end{array}$$

*The resolution of the above equations gives the following optimal controls*

$$\begin{array}{lll} & & u_{1}^{*}\left(t\right)=\frac{\left(\lambda_{2}-\lambda_{6}\right)\gamma_{2}E-w_{11}\left(t\right)+w_{12}\left(t\right)}{R_{1}},\\ & & u_{2}^{*}\left(t\right)=\frac{\left(\lambda_{4}-\lambda_{7}\right)\gamma_{1}I-w_{21}\left(t\right)+w_{22}\left(t\right)}{R_{2}},\\ & & u_{3}^{*}\left(t\right)=\frac{\left(\lambda_{3}-\lambda_{5}\right)\theta A+\lambda_{8}\left(\sigma I+a_{1}H+\omega_{1}A+\omega_{0}U\right)-w_{31}\left(t\right)+w_{32}\left(t\right)}{R_{3}}. \end{array}$$

*For the explicit expression of the optimal control*$u_{1}^{*}$*on* [0, *b*_1_], *we consider three cases*.

*First*, *when*$u_{1}^{*}\left(t\right)=0$, *we havew*_11_(*t*) = 0. *It then follows that*

$$0=u_{1}^{*}\left(t\right)=\frac{\left(\lambda_{2}-\lambda_{6}\right)\gamma_{2}E+w_{12}\left(t\right)}{R_{1}}.$$

*Owing tow*_12_ ≥ 0, *it follows that* $\frac{\left(\lambda_{2}-\lambda_{6}\right)\gamma_{2}E}{R_{1}}\leq 0$.

*Next*, *when*$0<u_{1}^{*}<b_{1}$, *it follows thatw*_11_(*t*) = 0 *and* *w*_12_ = 0. *Consequently*, *one has*

$$u_{1}^{*}\left(t\right)=\frac{\left(\lambda_{2}-\lambda_{6}\right)\gamma_{2}E}{R_{1}}.$$

*Finally*, *when*$u_{1}^{*}\left(t\right)=b_{1}$, *one getsw*_12_(*t*) = 0. *Thus*,

$$b_{1}=u_{1}^{*}\left(t\right)=\frac{\left(\lambda_{2}-\lambda_{6}\right)\gamma_{2}E-w_{11}\left(t\right)}{R_{1}}.$$

*This means thatR*_1_*b*_1_ = (*λ*_2_ − *λ*_6_)*γ*_2_*E* − *w*_11_(*t*), *so that* *w*_11_(*t*) = (*λ*_2_ − *λ*_6_)*γ*_2_*E* − *R*_1_ ≥ 0. *Thus* $\frac{\left(\lambda_{2}-\lambda_{6}\right)\gamma_{2}E}{R_{1}}\geq b_{1}$.

*From the above discussion*, *we obtain*

$$\begin{array}{lll} u_{1}^{*}=\left\{\begin{array}{ll} \frac{\left(\lambda_{2}-\lambda_{6}\right)\gamma_{2}E}{R_{1}},\; & \text{if}0<\frac{\left(\lambda_{2}-\lambda_{6}\right)\gamma_{2}E}{R_{1}}<b_{1}\\ 0,\; & \text{if}\frac{\left(\lambda_{2}-\lambda_{6}\right)\gamma_{2}E}{R_{1}}\leq 0\\ b_{1},\; & \text{if}\frac{\left(\lambda_{2}-\lambda_{6}\right)\gamma_{2}E}{R_{1}}\geq b_{1}. \end{array}\right) & & \end{array}$$

*This can also be written under the following compact form*

$$u_{1}^{*}\left(t\right)=\min\left\{\max\left\{0,\frac{\left(\lambda_{2}-\lambda_{6}\right)\gamma_{2}E}{R_{1}}\right\},b_{1}\right\}.$$

*Similarly*, *we get the following expressions for the second and third optimal control*

$$\begin{array}{lll} & & u_{2}^{*}\left(t\right)=\min\left\{\max\left\{0,\frac{\left(\lambda_{4}-\lambda_{7}\right)\gamma_{1}I}{R_{2}}\right\},b_{2}\right\},\\ & & u_{3}^{*}\left(t\right)=\min\left\{\max\left\{0,\frac{\left(\lambda_{3}-\lambda_{5}\right)\theta A+\lambda_{8}\left(\sigma I+a_{1}H+\omega_{1}A+\omega_{0}U\right)}{R_{3}}\right\},b_{3}\right\}. \end{array}$$

*Summarizing the above characterization we obtain the following result*.

Theorem 5.2 *Given the optimal controls*$\left(u_{1}^{*},u_{2}^{*},u_{3}^{*}\right)$*and the existence of solutions of system* (5.1)*, there exist adjoint variables* *λ*_*i*_*,* *i* = 1, …, 9 *satisfying the adjoint equations* (5.3) *together with the transversality conditions* *λ*_*i*_(*T*) = 0*, for* *i* = 1, …, 9*. Furthermore, the optimal controls* $u_{1}^{*}\left(t\right)$*,* $u_{2}^{*}\left(t\right)$ *and* $u_{3}^{*}\left(t\right)$ *are characterized as*

(5.4) $$\begin{array}{lll} & & u_{1}^{*}\left(t\right)=\min\left\{\max\left\{0,\frac{\left(\lambda_{2}-\lambda_{6}\right)\gamma_{2}E}{R_{1}}\right\},b_{1}\right\}, \end{array}$$

(5.5) $$\begin{array}{lll} & & u_{2}^{*}\left(t\right)=\min\left\{\max\left\{0,\frac{\left(\lambda_{4}-\lambda_{7}\right)\gamma_{1}I}{R_{2}}\right\},b_{2}\right\}, \end{array}$$

(5.6) $$\begin{array}{lll} & & u_{3}^{*}\left(t\right)=\min\left\{\max\left\{0,\frac{\left(\lambda_{3}-\lambda_{5}\right)\theta A+\lambda_{8}\left(\sigma I+a_{1}H+\omega_{1}A+\omega_{0}U\right)}{R_{3}}\right\},b_{3}\right\}. \end{array}$$

*We finally deal with the uniqueness for the optimality system including system* (5.1) *and adjoint* equation (5.3). *To do this*, *we need the following result*.

Lemma 5.3 (Garira et al., 2005; Joshi, 2002) *The function* $u_{1}^{*}\left(\varphi \right)=\min\left\{\max\left\{\varphi ,a_{2}^{1}\right\},b_{2}^{1}\right\}$ *is Lipschitz continuous with respect to* *φ, where* $a_{2}^{1}$ *and* $b_{2}^{1}$ *are two arbitrary fixed positive constants, with* $a_{2}^{1}<b_{2}^{1}$*.*

*Now*, *from the fact that the state variables are uniformly bounded*, *it can easily be shown that the adjoint variables have finite upper bounds*. *The uniqueness result for the optimality system states as follows*.

Theorem 5.4 *Bounded solutions of the optimality system are unique for a sufficiently smallT* > 0 *.*

*Proof*. *Let* (*S*, *E*, *A*, *I*, *U*, *Q*, *H*, *V*, *R*, *λ*_1_, *λ*_2_, *λ*_3_, *λ*_4_, *λ*_5_, *λ*_6_, *λ*_7_, *λ*_8_, *λ*_9_) *and* ($\bar{S}$, $\bar{E}$, $\bar{A}$, $\bar{I}$, $\bar{U}$, $\bar{Q}$, $\bar{H}$, $\bar{V}$, $\bar{R}$, ${\bar{\lambda }}_{1}$, ${\bar{\lambda }}_{2}$, ${\bar{\lambda }}_{3}$, ${\bar{\lambda }}_{4}$, ${\bar{\lambda }}_{5}$, ${\bar{\lambda }}_{6}$, ${\bar{\lambda }}_{7}$, ${\bar{\lambda }}_{8}$, ${\bar{\lambda }}_{9}$) *be two solutions of the optimality system*. *Set* *S* = *e*^*ϖt*^*p*_1_, *E* = *e*^*ϖt*^*p*_2_, *A* = *e*^*ϖt*^*p*_3_, *I* = *e*^*ϖt*^*p*_4_, *U* = *e*^*ϖt*^*p*_5_, *Q* = *e*^*ϖt*^*p*_6_, *H* = *e*^*ϖt*^*p*_7_, *V* = *e*^*ϖt*^*p*_8_, *R* = *e*^*ϖt*^*p*_9_, *λ*_1_ = *e*^−*ϖt*^*q*_1_, *λ*_2_ = *e*^−*ϖt*^*q*_2_, *λ*_3_ = *e*^−*ϖt*^*q*_3_, *λ*_4_ = *e*^−*ϖt*^*q*_4_, *λ*_5_ = *e*^−*ϖt*^*q*_5_, *λ*_6_ = *e*^−*ϖt*^*q*_6_, *λ*_7_ = *e*^−*ϖt*^*q*_7_, *λ*_8_ = *e*^−*ϖt*^*q*_8_, *λ*_9_ = *e*^−*ϖt*^*q*_9_. *Analogously*, *let* $\bar{S}=e^{\varpi t}{\bar{p}}_{1}$, $\bar{E}=e^{\varpi t}{\bar{p}}_{2}$, $\bar{A}=e^{\varpi t}{\bar{p}}_{3}$, $\bar{I}=e^{\varpi t}{\bar{p}}_{4}$, $\bar{U}=e^{\varpi t}{\bar{p}}_{5}$, $\bar{Q}=e^{\varpi t}{\bar{p}}_{6}$, $\bar{H}=e^{\varpi t}{\bar{p}}_{7}$, $\bar{V}=e^{\varpi t}{\bar{p}}_{8}$, $\bar{R}=e^{\varpi t}{\bar{p}}_{9}$, ${\bar{\lambda }}_{1}=e^{-\varpi t}{\bar{q}}_{1}$, ${\bar{\lambda }}_{2}=e^{-\varpi t}{\bar{q}}_{2}$, ${\bar{\lambda }}_{3}=e^{-\varpi t}{\bar{q}}_{3}$, ${\bar{\lambda }}_{4}=e^{-\varpi t}{\bar{q}}_{4}$, ${\bar{\lambda }}_{5}=e^{-\varpi t}{\bar{q}}_{5}$, ${\bar{\lambda }}_{6}=e^{-\varpi t}{\bar{q}}_{6}$, ${\bar{\lambda }}_{7}=e^{-\varpi t}{\bar{q}}_{7}$, ${\bar{\lambda }}_{8}=e^{-\varpi t}{\bar{q}}_{8}$, ${\bar{\lambda }}_{9}=e^{-\varpi t}{\bar{q}}_{9}$, *where* *ϖ* *is a positive constant*. *Define*

$$\begin{array}{lll} & & u_{1}^{*}\left(t\right)=\min\left\{\max\left\{0,\frac{\left(\lambda_{2}-\lambda_{6}\right)\gamma_{2}E}{R_{1}}\right\},b_{1}\right\},\\ & & u_{2}^{*}\left(t\right)=\min\left\{\max\left\{0,\frac{\left(\lambda_{4}-\lambda_{7}\right)\gamma_{1}I}{R_{2}}\right\},b_{2}\right\},\\ & & u_{3}^{*}\left(t\right)=\min\left\{\max\left\{0,\frac{\left(\lambda_{3}-\lambda_{5}\right)\theta A+\lambda_{8}\left(\sigma I+a_{1}H+\omega_{1}A+\omega_{0}U\right)}{R_{3}}\right\},b_{3}\right\}, \end{array}$$

*and*

$$\begin{array}{lll} & & {\bar{u}}_{1}^{*}\left(t\right)=\min\left\{\max\left\{0,\frac{\left({\bar{\lambda }}_{2}-{\bar{\lambda }}_{6}\right)\gamma_{2}\bar{E}}{R_{1}}\right\},b_{1}\right\},\\ & & {\bar{u}}_{2}^{*}\left(t\right)=\min\left\{\max\left\{0,\frac{\left({\bar{\lambda }}_{4}-{\bar{\lambda }}_{7}\right)\gamma_{1}\bar{I}}{R_{2}}\right\},b_{2}\right\},\\ & & {\bar{u}}_{3}^{*}\left(t\right)=\min\left\{\max\left\{0,\frac{\left({\bar{\lambda }}_{3}-{\bar{\lambda }}_{5}\right)\theta \bar{A}+\bar{\lambda_{8}}\left(\sigma \bar{I}+a_{1}\bar{H}+\omega_{1}\bar{A}+\omega_{0}\bar{U}\right)}{R_{3}}\right\},b_{3}\right\}. \end{array}$$

*Then*, *from*Lemma 5.3, *it follows that*

$$\begin{array}{lll} & & \left|u_{1}^{*}\left(t\right)-{\bar{u}}_{1}^{*}\left(t\right)\right|\leq \left|\frac{\gamma_{2}}{R_{1}}\left\{p_{2}\left(q_{2}-q_{6}\right)-{\bar{p}}_{2}\left({\bar{q}}_{2}-{\bar{q}}_{6}\right)\right\}\right|,\\ & & =\frac{\gamma_{2}}{R_{1}}|p_{2}\left(q_{2}-q_{6}\right)-{\bar{p}}_{2}\left({\bar{q}}_{2}-{\bar{q}}_{6}\right)|, \end{array}$$

$$\begin{array}{lll} & & \left|u_{2}^{*}\left(t\right)-{\bar{u}}_{2}^{*}\left(t\right)\right|\leq \left|\frac{\gamma_{1}}{R_{2}}\left\{p_{4}\left(q_{4}-q_{7}\right)-{\bar{p}}_{4}\left({\bar{q}}_{4}-{\bar{q}}_{7}\right)\right\}\right|,\\ & & =\frac{\gamma_{1}}{R_{2}}|p_{4}\left(q_{4}-q_{7}\right)-{\bar{p}}_{4}\left({\bar{q}}_{4}-{\bar{q}}_{7}\right)|, \end{array}$$

*and*

$$\begin{array}{lll} & & \left|u_{3}^{*}\left(t\right)-{\bar{u}}_{3}^{*}\left(t\right)\right|\leq |\frac{1}{R_{3}}\left\{\theta p_{3}\left(q_{3}-q_{5}\right)-{\bar{p}}_{3}\left({\bar{q}}_{3}-{\bar{q}}_{5}\right)+\sigma \left(p_{4}q_{8}-{\bar{p}}_{4}{\bar{q}}_{8}\right)\right)\\ & & \left(+a_{1}\left(p_{7}q_{8}-{\bar{p}}_{7}{\bar{q}}_{8}\right)+\omega_{1}\left(p_{3}q_{8}-{\bar{p}}_{3}{\bar{q}}_{8}\right)+\omega_{0}\left(p_{5}q_{8}-{\bar{p}}_{5}{\bar{q}}_{8}\right)\right\}|,\\ & & =\frac{1}{R_{3}}|\theta p_{3}\left(q_{3}-q_{5}\right)-{\bar{p}}_{3}\left({\bar{q}}_{3}-{\bar{q}}_{5}\right)+\sigma \left(p_{4}q_{8}-{\bar{p}}_{4}{\bar{q}}_{8}\right)\\ & & +a_{1}\left(p_{7}q_{8}-{\bar{p}}_{7}{\bar{q}}_{8}\right)+\omega_{1}\left(p_{3}q_{8}-{\bar{p}}_{3}{\bar{q}}_{8}\right)+\omega_{0}\left(p_{5}q_{8}-{\bar{p}}_{5}{\bar{q}}_{8}\right)|. \end{array}$$

*InsertingS* = *e*^*ϖt*^*p*_1_ *in the first equation of* (5.1) *yields*

(5.7) $$p_{1}^{\prime }+\varpi p_{1}=se^{-\varpi t}-\beta p_{1}\left(p_{3}+p_{4}+p_{5}+\eta_{1}p_{7}\right)e^{\varpi t}-a_{0}p_{1}p_{8}e^{\varpi t}-\mu p_{1}.$$

*In a similar way for the other state and adjoint variables*, *we obtain*

(5.8) $$\begin{array}{lll} & & p_{2}^{\prime }+\varpi p_{2}=\beta p_{1}\left(p_{3}+p_{4}+p_{5}+\eta_{1}p_{7}\right)e^{\varpi t}+a_{0}p_{1}p_{8}e^{\varpi t}-\left(\mu +k+\epsilon +b+\eta +\gamma_{2}u_{1}^{*}\right)p_{2}, \end{array}$$

(5.9) $$\begin{array}{lll} & & p_{3}^{\prime }+\varpi p_{3}=kp_{2}-\left(\mu +\delta_{4}+\gamma +\theta \left(1-u_{3}^{*}\right)p_{3}\right), \end{array}$$

(5.10) $$\begin{array}{lll} & & p_{4}^{\prime }+\varpi p_{4}=\eta p_{2}-\left(\mu +\delta_{1}+\rho +d_{0}+\gamma_{1}u_{2}^{*}\right)p_{4}, \end{array}$$

(5.11) $$\begin{array}{lll} & & p_{5}^{\prime }+\varpi p_{5}=bp_{2}+\theta \left(1-u_{3}^{*}\right)p_{3}-\left(\mu +\delta_{3}+\nu \right)p_{5}, \end{array}$$

(5.12) $$\begin{array}{lll} & & p_{6}^{\prime }+\varpi p_{6}=\left(\epsilon +\gamma_{2}u_{1}^{*}\right)p_{2}-\left(\mu +\alpha +d_{1}\right)p_{6}, \end{array}$$

(5.13) $$\begin{array}{lll} & & p_{7}^{\prime }+\varpi p_{7}=\left(d_{0}+\gamma_{1}u_{2}^{*}\right)p_{4}+d_{1}p_{6}-\left(\mu +\delta_{2}+r\right)p_{7}, \end{array}$$

(5.14) $$\begin{array}{lll} & & p_{8}^{\prime }+\varpi p_{8}=\sigma \left(1-u_{3}^{*}\right)p_{4}+a_{1}\left(1-u_{3}^{*}\right)p_{7}+\omega_{1}\left(1-u_{3}^{*}\right)p_{3}+\omega_{0}\left(1-u_{3}^{*}\right)p_{5}-\delta_{5}p_{8}, \end{array}$$

(5.15) $$\begin{array}{lll} & & p_{9}^{\prime }+\varpi p_{9}=\rho p_{4}+\nu p_{5}+\gamma p_{3}+\alpha p_{6}+rp_{7}-\mu p_{9}, \end{array}$$

(5.16) $$\begin{array}{lll} & & -q_{1}^{\prime }+\varpi q_{1}=-\mu q_{1}-\beta q_{1}\left(p_{3}+p_{4}+p_{5}+\eta_{1}p_{7}\right)e^{\varpi t}+a_{0}q_{1}p_{8}e^{\varpi t}\\ & & \;\;\;+\beta q_{2}\left(p_{3}+p_{4}+p_{5}+\eta_{1}p_{7}\right)e^{\varpi t}+a_{0}q_{2}p_{8}e^{\varpi t}, \end{array}$$

(5.17) $$\begin{array}{lll} & & -q_{2}^{\prime }+\varpi q_{2}=B_{1}e^{\varpi t}-\left(\mu +k+\epsilon +b+\eta +\gamma_{2}u_{1}^{*}\right)q_{2}\\ & & \;\;\;+kq_{3}+\eta q_{4}+bq_{5}+\left(\epsilon +\gamma_{2}u_{1}^{*}\right)q_{6}, \end{array}$$

(5.18) $$\begin{array}{lll} & & -q_{3}^{\prime }+\varpi q_{3}=B_{2}e^{\varpi t}-\beta p_{1}q_{1}e^{\varpi t}-\left(\mu +\delta_{4}+\gamma +\theta \left(1-u_{3}^{*}\right)q_{3}\right)\\ & & \;\;\;+\beta p_{1}q_{2}e^{2\varpi t}+\theta \left(1-u_{3}^{*}\right)q_{5}+\omega_{1}\left(1-u_{3}^{*}\right)q_{8}+\gamma q_{9}, \end{array}$$

(5.19) $$\begin{array}{lll} & & -q_{4}^{\prime }+\varpi q_{4}=B_{3}e^{\varpi t}-\beta p_{1}q_{1}e^{\varpi t}-\left(\mu +\delta_{1}+\rho +d_{0}+\gamma_{1}u_{2}^{*}\right)q_{4}\\ & & \;\;\;+\beta p_{1}q_{2}e^{\varpi t}+\left(d_{0}+\gamma_{1}u_{2}^{*}\right)q_{7}+\sigma \left(1-u_{3}^{*}\right)q_{8}+\rho q_{9}, \end{array}$$

(5.20) $$\begin{array}{lll} & & -q_{5}^{\prime }+\varpi q_{5}=B_{4}e^{\varpi t}-\beta p_{1}q_{1}e^{\varpi t}-\left(\mu +\delta_{3}+\nu \right)q_{5}\\ & & \;\;\;+\beta p_{1}q_{2}e^{\varpi t}+\omega_{0}\left(1-u_{3}\right)q_{8}+\nu q_{9}, \end{array}$$

(5.21) $$\begin{array}{lll} & & -q_{6}^{\prime }+\varpi q_{6}=B_{5}e^{\varpi t}-\left(\mu +\alpha +d_{1}\right)q_{6}+d_{1}q_{7}+\alpha q_{9} \end{array}$$

(5.22) $$\begin{array}{lll} & & -q_{7}^{\prime }+\varpi q_{7}=B_{6}e^{\varpi t}-\beta \eta_{1}p_{1}q_{1}e^{\varpi t}-\left(\mu +\delta_{2}+r\right)q_{7}\\ & & \;\;\;+\beta \eta_{1}p_{1}q_{2}e^{\varpi t}+a_{1}\left(1-u_{3}^{*}\right)q_{8}-rq_{9}, \end{array}$$

(5.23) $$\begin{array}{lll} & & -q_{8}^{\prime }+\varpi q_{8}=B_{7}e^{\varpi t}-a_{0}p_{1}q_{1}e^{\varpi t}+a_{0}p_{1}q_{2}e^{\varpi t}-\delta_{5}q_{8}, \end{array}$$

(5.24) $$-q_{9}^{\prime }+\varpi q_{9}=-\mu q_{9}.$$

*Also*, *introducing*$\bar{S}=e^{\varpi t}{\bar{p}}_{1}$*in the first equation of*(5.1)*yields*

(5.25) $${\bar{p}}_{1}^{\prime }+\varpi {\bar{p}}_{1}=se^{-\varpi t}-\beta {\bar{p}}_{1}\left({\bar{p}}_{3}+{\bar{p}}_{4}+{\bar{p}}_{5}+\eta_{1}{\bar{p}}_{7}\right)e^{\varpi t}-a_{0}{\bar{p}}_{1}{\bar{p}}_{8}e^{\varpi t}-\mu {\bar{p}}_{1}.$$

*Then*, *the result is obtained by subtracting the equations forSand*$\bar{S}$, *Eand*$\bar{E}$, *Aand*$\bar{A}$, *Iand*$\bar{I}$, *Uand*$\bar{U}$, *Qand*$\bar{Q}$, *Hand*$\bar{H}$, *Vand*$\bar{V}$, *Rand*$\bar{R}$, *λ*_1_*and*${\bar{\lambda }}_{1}$, *λ*_2_*and*${\bar{\lambda }}_{2}$, *λ*_3_*and*${\bar{\lambda }}_{3}$, *λ*_4_*and*${\bar{\lambda }}_{4}$, *λ*_5_*and*${\bar{\lambda }}_{5}$, *λ*_6_*and*${\bar{\lambda }}_{6}$, *λ*_7_*and*${\bar{\lambda }}_{7}$, *λ*_8_*and*${\bar{\lambda }}_{8}$, *λ*_9_*and*${\bar{\lambda }}_{9}$, *multiplying each resulting equation by an appropriate difference of functions*, *and integrating from* 0 *to* *T*. *For example*, *subtracting* equation (5.7) *from* (5.25) *leads to*

(5.26) $$\begin{array}{lll} & & {\left(p_{1}-{\bar{p}}_{1}\right)}^{\prime }+\varpi \left(p_{1}-{\bar{p}}_{1}\right)=-\beta e^{\varpi t}\left[p_{1}\left(p_{3}+p_{4}+p_{5}+\eta_{1}p_{7}\right)-{\bar{p}}_{1}\left({\bar{p}}_{3}+{\bar{p}}_{4}+{\bar{p}}_{5}+\eta_{1}{\bar{p}}_{7}\right)\right]\\ & & \;\;\;\;\;\;-a_{0}e^{\varpi t}\left[p_{1}p_{8}-{\bar{p}}_{1}{\bar{p}}_{8}\right]-\mu \left(p_{1}-{\bar{p}}_{1}\right). \end{array}$$

*Multiplying the left and right hand sides of*(5.26)*by*$\left(p_{1}-{\bar{p}}_{1}\right)$*and integrating from* 0 *to* *T* *gives*

$$\begin{array}{lll} & & \frac{1}{2}{\left(p_{1}-{\bar{p}}_{1}\right)}^{2}+\varpi \int_{0}^{T}{\left(p_{1}-{\bar{p}}_{1}\right)}^{2}dt\\ & & \leq \mu \int_{0}^{T}{\left(p_{1}-{\bar{p}}_{1}\right)}^{2}dt+C_{1}e^{\varpi T}\int_{0}^{T}\left[{\left(p_{1}-{\bar{p}}_{1}\right)}^{2}+{\left(p_{3}-{\bar{p}}_{3}\right)}^{2}\right)\\ & & \left(\;+{\left(p_{4}-{\bar{p}}_{4}\right)}^{2}+{\left(p_{5}-{\bar{p}}_{5}\right)}^{2}+{\left(p_{7}-{\bar{p}}_{7}\right)}^{2}+{\left(p_{8}-{\bar{p}}_{8}\right)}^{2}\right]dt, \end{array}$$

*where the constant* *C*_1_ *depends on the coefficients and the bounds on state variables* *p*_1_, *p*_3_, *p*_4_, *p*_5_ *and* *p*_7_. *Noting that* *e*^*ϖT*^ ≤ *e*^3*ϖT*^, *we get*

(5.27) $$\begin{array}{lll} & & \frac{1}{2}{\left(p_{1}-{\bar{p}}_{1}\right)}^{2}+\varpi \int_{0}^{T}{\left(p_{1}-{\bar{p}}_{1}\right)}^{2}dt\\ & & \leq C_{1}^{\prime }\int_{0}^{T}\left[{\left(p_{1}-{\bar{p}}_{1}\right)}^{2}dt+C_{2}^{\prime }e^{3\varpi T}\int_{0}^{T}\left[{\left(p_{1}-{\bar{p}}_{1}\right)}^{2}+{\left(p_{3}-{\bar{p}}_{3}\right)}^{2}\right)\right)\\ & & \left(\;+{\left(p_{4}-{\bar{p}}_{4}\right)}^{2}+{\left(p_{5}-{\bar{p}}_{5}\right)}^{2}+{\left(p_{7}-{\bar{p}}_{7}\right)}^{2}+{\left(p_{8}-{\bar{p}}_{8}\right)}^{2}\right]dt. \end{array}$$

$C_{1}^{\prime }$*and*$C_{2}^{\prime }$*depend on the coefficients and the upper bounds of state variablesp*_1_, *p*_3_, *p*_4_, *p*_5_, *p*_7_, *p*_7_.

*Now*, *introducing*${\bar{\lambda }}_{2}=e^{-\varpi t}{\bar{q}}_{2}$*in the second equation of*(5.3), *we obtain*

(5.28) $$\begin{array}{lll} & & -{\bar{q}}_{2}^{\prime }+\varpi {\bar{q}}_{2}=B_{1}e^{\varpi t}-\left(\mu +k+\epsilon +b+\eta +\gamma_{2}{\bar{u}}_{1}^{*}\right)q_{2}\\ & & \;\;\;+k{\bar{q}}_{3}+\eta {\bar{q}}_{4}+b{\bar{q}}_{5}+\left(\epsilon +\gamma_{2}{\bar{u}}_{1}^{*}\right){\bar{q}}_{6}. \end{array}$$

*Subtracting* equation (5.17) *from* (5.27), *yields*

(5.29) $$\begin{array}{lll} & & -{\left(q_{1}-{\bar{q}}_{2}\right)}^{\prime }+\varpi \left(q_{2}-{\bar{q}}_{2}\right)=-\left[\left(k_{1}+\gamma_{2}u_{1}^{*}\right)q_{2}-\left(k_{1}+\gamma_{2}{\bar{u}}_{1}^{*}\right){\bar{q}}_{2}\right]\\ & & \;+k\left(q_{3}-{\bar{q}}_{3}\right)+\eta \left(q_{4}-{\bar{q}}_{4}\right)+b\left(q_{5}-{\bar{q}}_{5}\right)+\left[\left(\epsilon +\gamma_{2}u_{1}^{*}\right)q_{6}-\left(\epsilon +\gamma_{2}{\bar{u}}_{1}^{*}\right){\bar{q}}_{6}\right], \end{array}$$

*where* *k*_1_ = *μ* + *k* + *ϵ* + *b* + *η*. *Multiplying the left and right hand sides of* (5.28) *by* $\left(q_{2}-{\bar{q}}_{2}\right)$ *and integrating from* 0 *to* *T* *gives*

$$\begin{array}{lll} & & \frac{1}{2}{\left(q_{2}\left(0\right)-{\bar{q}}_{2}\left(0\right)\right)}^{2}+\varpi \int_{0}^{T}{\left(q_{2}-{\bar{q}}_{2}\right)}^{2}dt\\ & & =-k_{1}\int_{0}^{T}{\left(q_{2}-{\bar{q}}_{2}\right)}^{2}dt-\gamma_{2}\int_{0}^{T}\left(u_{1}^{*}q_{2}-{\bar{u}}_{1}^{*}{\bar{q}}_{2}\right)\left(q_{2}-{\bar{q}}_{2}\right)dt\\ & & \;\;+k\int_{0}^{T}\left(q_{2}-{\bar{q}}_{2}\right)\left(q_{3}-{\bar{q}}_{3}\right)dt+\eta \int_{0}^{T}\left(q_{2}-{\bar{q}}_{2}\right)\left(q_{4}-{\bar{q}}_{4}\right)dt\\ & & \;\;+b\int_{0}^{T}\left(q_{2}-{\bar{q}}_{2}\right)\left(q_{5}-{\bar{q}}_{5}\right)dt+\epsilon \int_{0}^{T}\left(q_{2}-{\bar{q}}_{2}\right)\left(q_{6}-{\bar{q}}_{6}\right)dt+\gamma_{2}\int_{0}^{T}\left(u_{1}^{*}q_{6}-{\bar{u}}_{1}^{*}{\bar{q}}_{6}\right)\left(q_{2}-{\bar{q}}_{2}\right)dt. \end{array}$$

*The last term of the above equation reads*

(5.30) $$\begin{array}{lll} & & \gamma_{2}\int_{0}^{T}\left(u_{1}^{*}q_{6}-{\bar{u}}_{1}^{*}{\bar{q}}_{6}\right)\left(q_{2}-{\bar{q}}_{2}\right)dt=\gamma_{2}\int_{0}^{T}u_{1}^{*}\left(q_{6}-{\bar{q}}_{6}\right)\left(q_{2}-{\bar{q}}_{2}\right)+{\bar{q}}_{2}\left(u_{1}^{*}-{\bar{u}}_{1}^{*}\right)\left(q_{2}-{\bar{q}}_{2}\right)dt,\\ & & \leq C_{01}\int_{0}^{T}\left[{\left(q_{2}-{\bar{q}}_{2}\right)}^{2}+{\left(q_{6}-{\bar{q}}_{6}\right)}^{2}+{\left(u_{1}^{*}-{\bar{u}}_{1}^{*}\right)}^{2}\right]dt. \end{array}$$

*Now*

(5.30) $$\begin{array}{lll} & & \int_{0}^{T}{\left(u_{1}^{*}-{\bar{u}}_{1}^{*}\right)}^{2}dt\leq {\left(\frac{\gamma_{2}}{R_{1}}\right)}^{2}\int_{0}^{T}{\left[p_{2}\left(q_{2}-q_{6}\right)-{\bar{p}}_{2}\left({\bar{q}}_{2}-{\bar{q}}_{6}\right)\right]}^{2}dt,\\ & & ={\left(\frac{\gamma_{2}}{R_{1}}\right)}^{2}\int_{0}^{T}\left[p_{2}^{2}{\left(q_{2}-q_{6}\right)}^{2}-2p_{2}\left(q_{2}-q_{6}\right){\bar{p}}_{2}\left({\bar{q}}_{2}-{\bar{q}}_{6}\right)+{\bar{p}}_{2}^{2}{\left({\bar{q}}_{2}-{\bar{q}}_{6}\right)}^{2}\right]dt,\\ & & \leq C_{03}{\left(\frac{\gamma_{2}}{R_{1}}\right)}^{2}\int_{0}^{T}\left[{\left(q_{2}-{\bar{q}}_{2}\right)}^{2}+{\left(q_{6}-{\bar{q}}_{6}\right)}^{2}\right]dt. \end{array}$$

*From this inequality*, (5.29) *becomes*

$$\gamma_{2}\int_{0}^{T}\left(\mu_{1}^{*}q_{6}-{\bar{\mu }}_{1}^{*}{\bar{q}}_{6}\right)\left(q_{2}-{\bar{q}}_{2}\right)dt\leq C_{4}\int_{0}^{T}\left[{\left(q_{2}-{\bar{q}}_{2}\right)}^{2}+{\left(q_{6}-{\bar{q}}_{6}\right)}^{2}\right]dt.$$

*The constantsC*_01_, *C*_03_, *C*_04_, *C*_3_*andC*_4_*which appear in the preceding inequalities depend on the coefficients and the bounds on state and adjoint variables*. *Consequently*, *we get*

(5.31) $$\begin{array}{lll} & & \frac{1}{2}{\left(q_{2}\left(0\right)-{\bar{q}}_{2}\left(0\right)\right)}^{2}+\varpi \int_{0}^{T}{\left(q_{2}-{\bar{q}}_{2}\right)}^{2}dt\\ & & \leq k\int_{0}^{T}\left[{\left(q_{2}-{\bar{q}}_{2}\right)}^{2}+{\left(q_{3}-{\bar{q}}_{3}\right)}^{2}\right]dt+\eta \int_{0}^{T}\left[{\left(q_{2}-{\bar{q}}_{2}\right)}^{2}+{\left(q_{4}-{\bar{q}}_{4}\right)}^{2}\right]dt\\ & & +b\int_{0}^{T}\left[{\left(q_{2}-{\bar{q}}_{2}\right)}^{2}+{\left(q_{5}-{\bar{q}}_{5}\right)}^{2}\right]dt+\epsilon \int_{0}^{T}\left[{\left(q_{2}-{\bar{q}}_{2}\right)}^{2}+{\left(q_{6}-{\bar{q}}_{6}\right)}^{2}\right]dt\\ & & +C_{3}^{\prime }\int_{0}^{T}\left[{\left(q_{2}-{\bar{q}}_{2}\right)}^{2}+{\left(q_{3}-{\bar{q}}_{3}\right)}^{2}+{\left(q_{4}-{\bar{q}}_{4}\right)}^{2}+{\left(q_{5}-{\bar{q}}_{5}\right)}^{2}\right)\\ & & \left(+{\left(q_{6}-{\bar{q}}_{6}\right)}^{2}+{\left(q_{7}-{\bar{q}}_{7}\right)}^{2}+{\left(q_{8}-{\bar{q}}_{8}\right)}^{2}+{\left(q_{9}-{\bar{q}}_{9}\right)}^{2}\right]dt, \end{array}$$

*where the constant*$C_{3}^{\prime }$*depends on the coefficients and the upper bounds of state and adjoint variables*.

*Using the same reasoning for the remaining eight state and adjoint variables*, *we obtain their integral equations and their estimates*. *The combination of these eighteen estimates yields*

$$\begin{array}{lll} & & \frac{1}{2}{\left(p_{1}-{\bar{p}}_{1}\right)}^{2}\left(T\right)+\frac{1}{2}{\left(p_{2}-{\bar{p}}_{2}\right)}^{2}\left(T\right)+\frac{1}{2}{\left(p_{3}-{\bar{p}}_{3}\right)}^{2}\left(T\right)+\frac{1}{2}{\left(p_{4}-{\bar{p}}_{4}\right)}^{2}\left(T\right)\\ & & +\frac{1}{2}{\left(p_{5}-{\bar{p}}_{5}\right)}^{2}\left(T\right)+\frac{1}{2}{\left(p_{6}-{\bar{p}}_{6}\right)}^{2}\left(T\right)+\frac{1}{2}{\left(p_{7}-{\bar{p}}_{7}\right)}^{2}\left(T\right)+\frac{1}{2}{\left(p_{8}-{\bar{p}}_{8}\right)}^{2}\left(T\right)\\ & & +\frac{1}{2}{\left(p_{9}-{\bar{p}}_{9}\right)}^{2}\left(T\right)+\frac{1}{2}{\left(q_{1}-{\bar{q}}_{1}\right)}^{2}\left(0\right)+\frac{1}{2}{\left(q_{2}-{\bar{q}}_{2}\right)}^{2}\left(0\right)+\frac{1}{2}{\left(q_{3}-{\bar{q}}_{3}\right)}^{2}\left(0\right)\\ & & +\frac{1}{2}{\left(q_{4}-{\bar{q}}_{4}\right)}^{2}\left(0\right)+\frac{1}{2}{\left(q_{5}-{\bar{q}}_{5}\right)}^{2}\left(0\right)+\frac{1}{2}{\left(q_{6}-{\bar{q}}_{6}\right)}^{2}\left(0\right)\\ & & +\frac{1}{2}{\left(q_{7}-{\bar{q}}_{7}\right)}^{2}\left(0\right)+\frac{1}{2}{\left(q_{8}-{\bar{q}}_{8}\right)}^{2}\left(0\right)+\frac{1}{2}{\left(q_{9}-{\bar{q}}_{9}\right)}^{2}\left(0\right)\\ & & +\varpi \int_{0}^{T}\left[{\left(p_{1}-{\bar{p}}_{1}\right)}^{2}+{\left(p_{2}-{\bar{p}}_{2}\right)}^{2}+{\left(p_{3}-{\bar{p}}_{3}\right)}^{2}+{\left(p_{4}-{\bar{p}}_{4}\right)}^{2}+{\left(p_{5}-{\bar{p}}_{5}\right)}^{2}+{\left(p_{6}-{\bar{p}}_{6}\right)}^{2}\right)\\ & & +{\left(p_{7}-{\bar{p}}_{7}\right)}^{2}+{\left(p_{8}-{\bar{p}}_{8}\right)}^{2}+{\left(p_{9}-{\bar{p}}_{9}\right)}^{2}+{\left(q_{1}-{\bar{q}}_{1}\right)}^{2}+{\left(q_{2}-{\bar{q}}_{2}\right)}^{2}+{\left(q_{3}-{\bar{q}}_{3}\right)}^{2}\\ & & \left(+{\left(q_{4}-{\bar{q}}_{4}\right)}^{2}+{\left(q_{5}-{\bar{q}}_{5}\right)}^{2}+{\left(q_{6}-{\bar{q}}_{6}\right)}^{2}+{\left(q_{7}-{\bar{q}}_{7}\right)}^{2}+{\left(q_{8}-{\bar{q}}_{8}\right)}^{2}+{\left(q_{9}-{\bar{q}}_{9}\right)}^{2}\right]dt\\ & & \leq \left({\tilde{D}}_{1}+{\tilde{D}}_{2}e^{3\varpi T}\right)\int_{0}^{T}\left[{\left(p_{1}-{\bar{p}}_{1}\right)}^{2}+{\left(p_{2}-{\bar{p}}_{2}\right)}^{2}+{\left(p_{3}-{\bar{p}}_{3}\right)}^{2}\right) \end{array}$$

$$\begin{array}{lll} & & +{\left(p_{4}-{\bar{p}}_{4}\right)}^{2}+{\left(p_{5}-{\bar{p}}_{5}\right)}^{2}+{\left(p_{6}-{\bar{p}}_{6}\right)}^{2}+{\left(p_{7}-{\bar{p}}_{7}\right)}^{2}+{\left(p_{8}-{\bar{p}}_{8}\right)}^{2}+{\left(p_{9}-{\bar{p}}_{9}\right)}^{2}\\ & & +{\left(q_{1}-{\bar{q}}_{1}\right)}^{2}+{\left(q_{2}-{\bar{q}}_{2}\right)}^{2}+{\left(q_{3}-{\bar{q}}_{3}\right)}^{2}+{\left(q_{4}-{\bar{q}}_{4}\right)}^{2}+{\left(q_{5}-{\bar{q}}_{5}\right)}^{2}+{\left(q_{6}-{\bar{q}}_{6}\right)}^{2}\\ & & \left(+{\left(q_{7}-{\bar{q}}_{7}\right)}^{2}+{\left(q_{8}-{\bar{q}}_{8}\right)}^{2}+{\left(q_{9}-{\bar{q}}_{9}\right)}^{2}\right]dt. \end{array}$$

*From this*, *we deduce that*

(5.32) $$\begin{array}{lll} & & \left(\varpi -{\tilde{D}}_{1}-{\tilde{D}}_{2}e^{3\varpi T}\right)\int_{0}^{T}\left[{\left(p_{1}-{\bar{p}}_{1}\right)}^{2}+{\left(p_{2}-{\bar{p}}_{2}\right)}^{2}+{\left(p_{3}-{\bar{p}}_{3}\right)}^{2}\right)\\ & & +{\left(p_{4}-{\bar{p}}_{4}\right)}^{2}+{\left(p_{5}-{\bar{p}}_{5}\right)}^{2}+{\left(p_{6}-{\bar{p}}_{6}\right)}^{2}+{\left(p_{7}-{\bar{p}}_{7}\right)}^{2}+{\left(p_{8}-{\bar{p}}_{8}\right)}^{2}+{\left(p_{9}-{\bar{p}}_{9}\right)}^{2}\\ & & +{\left(q_{1}-{\bar{q}}_{1}\right)}^{2}+{\left(q_{2}-{\bar{q}}_{2}\right)}^{2}+{\left(q_{3}-{\bar{q}}_{3}\right)}^{2}+{\left(q_{4}-{\bar{q}}_{4}\right)}^{2}+{\left(q_{5}-{\bar{q}}_{5}\right)}^{2}+{\left(q_{6}-{\bar{q}}_{6}\right)}^{2}\\ & & \left(+{\left(q_{7}-{\bar{q}}_{7}\right)}^{2}+{\left(q_{8}-{\bar{q}}_{8}\right)}^{2}+{\left(q_{9}-{\bar{q}}_{9}\right)}^{2}\right]dt\leq 0, \end{array}$$

*where the constants*${\tilde{D}}_{1}$*and*${\tilde{D}}_{2}$*depend on the coefficients and the upper bounds on state and adjoint variables*.

*By choosing*$\varpi >{\tilde{D}}_{1}+{\tilde{D}}_{2}$*and*$T<\frac{1}{3\varpi }\ln\left(\frac{\varpi -{\tilde{D}}_{1}}{{\tilde{D}}_{2}}\right)$, *it follows that*$p_{i}={\bar{p}}_{i}$*and*$q_{i}={\bar{q}}_{i}$, *fori* = 1, …, 9. *Thus*, *the solution of the optimality system is unique for* *T* *sufficiently small*. □

Theorem 5.3 *implies that the unique optimal controls*$u_{1}^{*}$, $u_{2}^{*}$*and*$u_{3}^{*}$*are characterized in terms of the unique solution of the optimality system*.

## 6 Numerical simulations

In this section, we simulate the COVID-19 model (2.2) as a function of time. Recall that COVID-19 is eliminated from the population if $R_{c}<1$ and persists whenever $R_{c}>1$. The parameter values used here are given in Table 1. Most of the parameters were obtained from (Tang et al., 2020), *ν* is from (Liu et al., 2020), *μ* is from (WHO, 2020a, 2020b) and some are chosen arbitrarily to satisfy the stability property of the disease-free equilibrium as well as the endemic equilibrium of the COVID-19 model (2.2). Taking the parameter values from Table 1, except: *β* = 3.62 × 10^−7^, *η*_1_ = 0.9, *a*_0_ = 10^−12^, *μ* = 1/57, *d*_1_ = 0.156986, we obtain $R_{c}=0.3073<1$. Here we consider the following four sets of initial conditions:

Initial-1: (*S*(0), *E*(0), *A*(0), *I*(0), *U*(0), *Q*(0), *H*(0), *V*(0), *R*(0)) = (12081, 8, 0, 0, 0, 80, 10, 1, 0),
Initial-2: (*S*(0), *E*(0), *A*(0), *I*(0), *U*(0), *Q*(0), *H*(0), *V*(0), *R*(0)) = (20081, 16, 0.5, 0.2, 0.3, 100, 20, 1.4, 10),
Initial-3: (*S*(0), *E*(0), *A*(0), *I*(0), *U*(0), *Q*(0), *H*(0), *V*(0), *R*(0)) = (50000, 26, 1.3, 0.4, 0.7, 200, 28, 2, 20),
Initial-4: (*S*(0), *E*(0), *A*(0), *I*(0), *U*(0), *Q*(0), *H*(0), *V*(0), *R*(0)) = (100000, 33, 2.3, 0.55, 1.1, 320, 40, 4, 40).

**Fig. 5:**
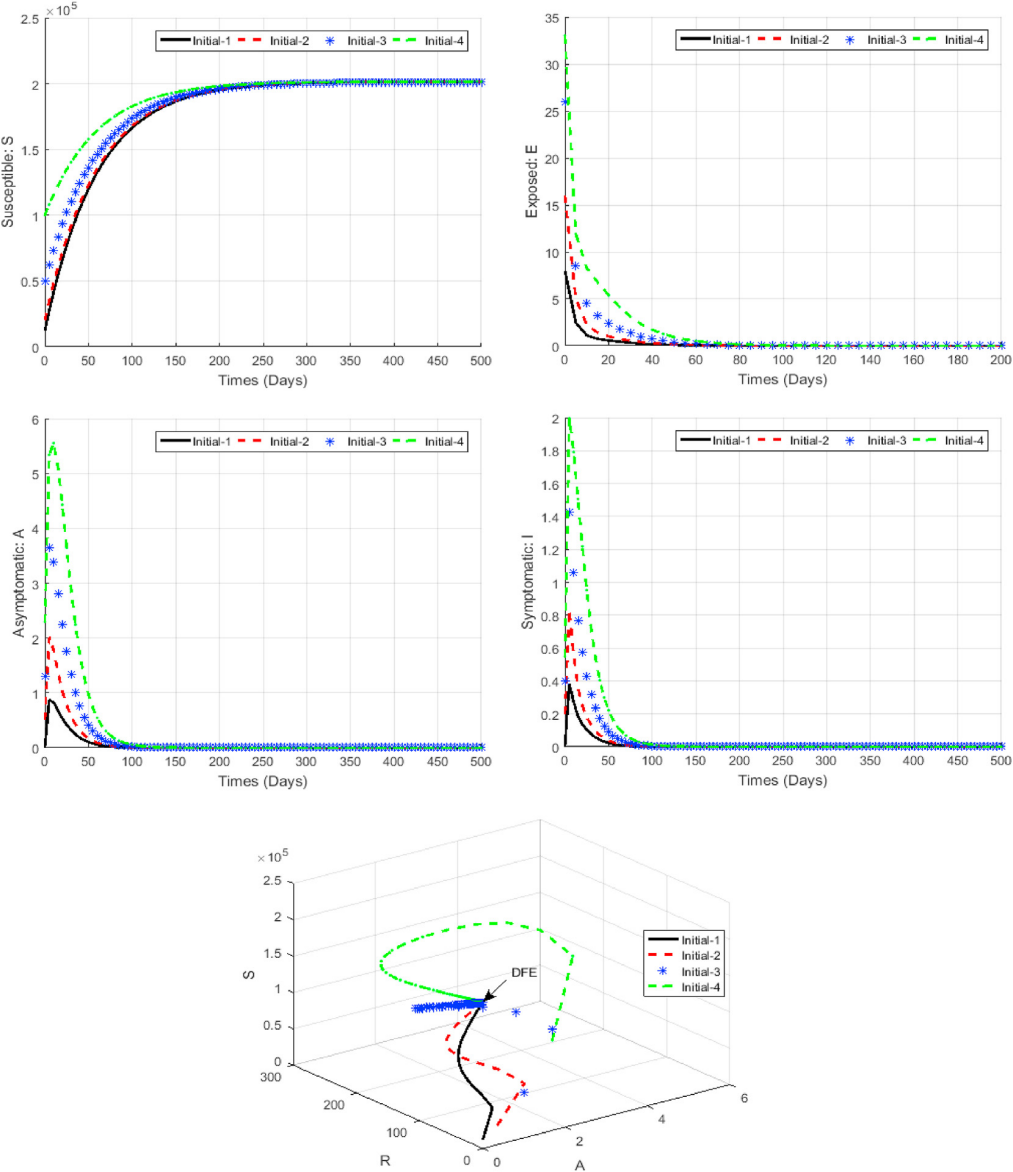
Simulation of the COVID-19 model (2.2) as a function of time using various initial conditions and the parameter values from Table 1 except *β* = 3.62 × 10^−7^, *η*_1_ = 0.9, *a*_0_ = 10^−12^, *μ* = 1/57, *d*_1_ = 0.156986, and $R_{c}=0.3073<1$.

**Fig. 6:**
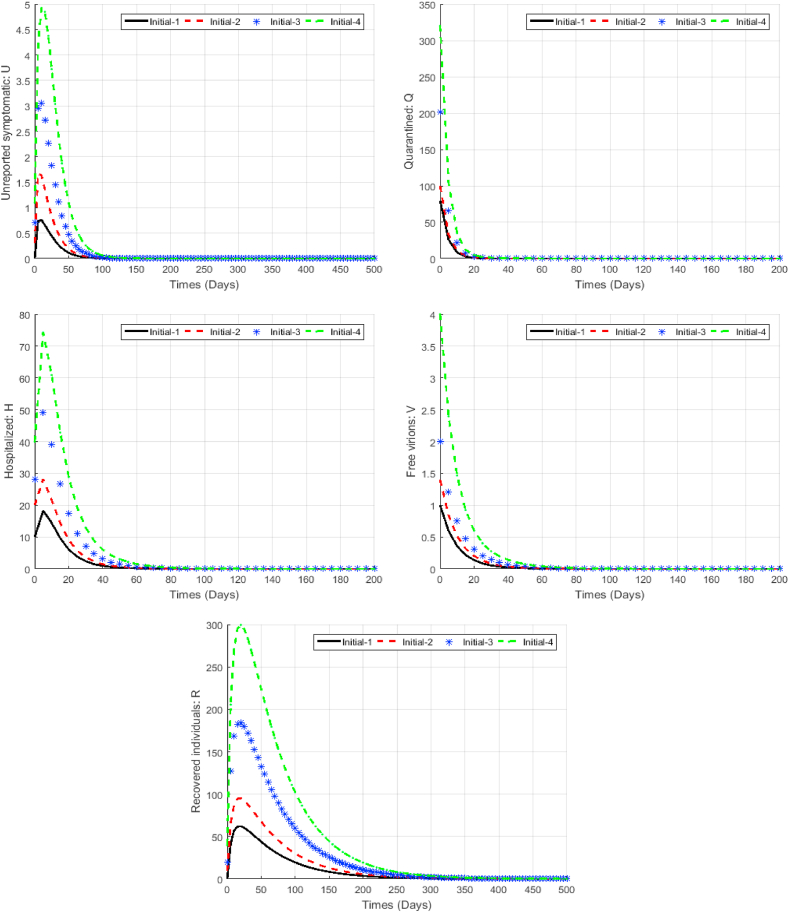
Simulation of the COVID-19 model (2.2) as a function of time using various initial conditions and the parameter values from Table 1 except *β* = 3.62 × 10^−7^, *η*_1_ = 0.9, *a*_0_ = 10^−12^, *μ* = 1/57, *d*_1_ = 0.156986, and $R_{c}=0.3073<1$.

In these cases, the disease-free equilibrium $E_{0}$ is globally asymptotically stable. Fig. 5, Fig. 6 clearly confirm this fact and we also observe that the COVID-19 system initiating with Initial-1, Initial-2, Initial-3 and Initial-4 approaches the disease-free equilibrium $E_{0}=\left(2\times 10^{5},0,0,0,0,0,0,0,0\right)$. Thus, the numerical findings support Theorem 4.1. This illustrates the fact that COVID-19 could be eliminated from the Cameroonian population.

Again considering the parameter values from Table 1 and taking *β* = 2.08 × 10^−6^, *η*_1_ = 0.49, *a*_0_ = 10^−7^, *μ* = 1/59, *d*_1_ = 0.156986, we obtain $R_{c}=1.6543>1$. Here we also consider four sets of initial conditions:

Initial-5: (*S*(0), *E*(0), *A*(0), *I*(0), *U*(0), *Q*(0), *H*(0), *V*(0), *R*(0)) = (120810, 8, 0, 0, 0, 80, 10, 1, 0),
Initial-6: (*S*(0), *E*(0), *A*(0), *I*(0), *U*(0), *Q*(0), *H*(0), *V*(0), *R*(0)) = (170810, 16, 0.5, 0.2, 0.3, 100, 20, 1.4, 10),
Initial-7: (*S*(0), *E*(0), *A*(0), *I*(0), *U*(0), *Q*(0), *H*(0), *V*(0), *R*(0)) = (280050, 26, 1.3, 0.4, 0.7, 200, 28, 2, 20),
Initial-8: (*S*(0), *E*(0), *A*(0), *I*(0), *U*(0), *Q*(0), *H*(0), *V*(0), *R*(0)) = (550000, 33, 2.3, 0.55, 1.1, 320, 40, 4, 40).

**Fig. 7:**
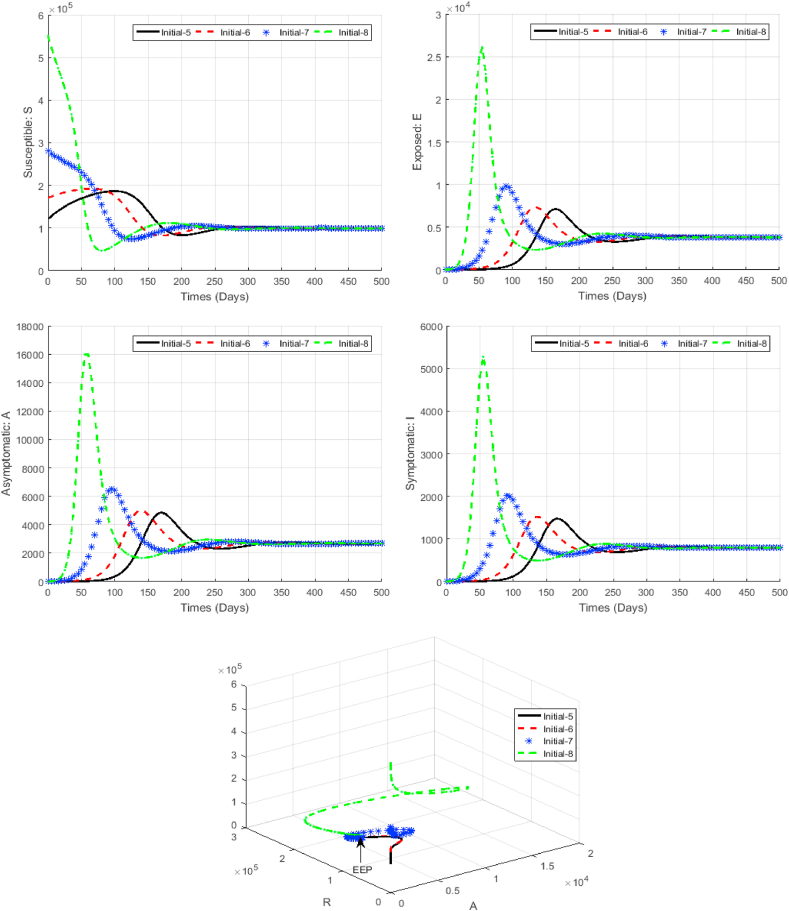
Simulation of the COVID-19 model (2.2) as a function of time using various initial conditions and the parameter values from Table 1 except *β* = 2.08 × 10^−6^, *η*_1_ = 0.49, *a*_0_ = 10^−7^, *μ* = 1/59, *d*_1_ = 0.156986, and $R_{c}=1.6543>1$.

**Fig. 8:**
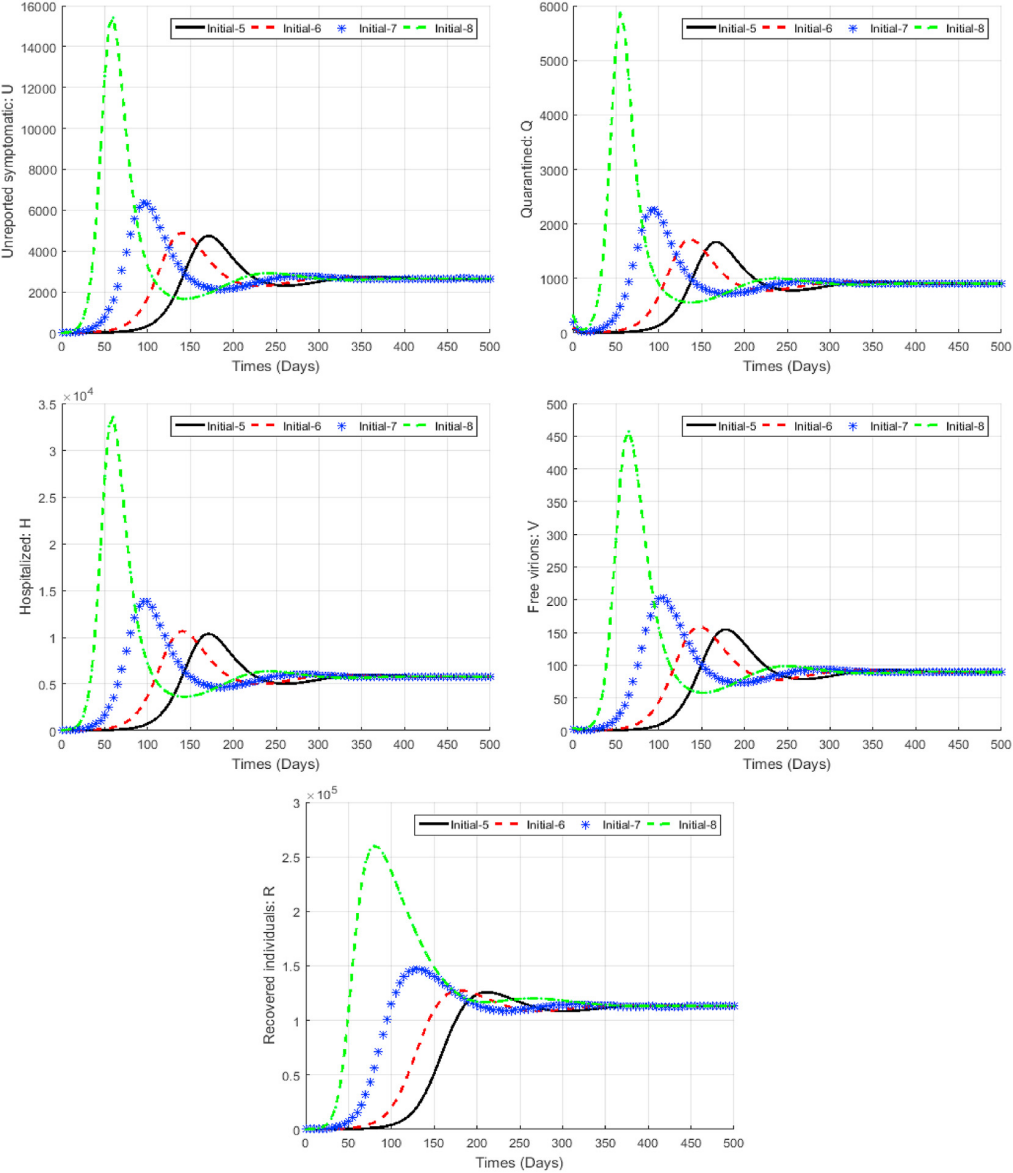
Simulation of the COVID-19 model (2.2) as a function of time using various initial conditions and the parameter values from Table 1 except *β* = 2.08 × 10^−6^, *η*_1_ = 0.49, *a*_0_ = 10^−7^, *μ* = 1/59, *d*_1_ = 0.156986, and $R_{c}=1.6543>1$.

It follows that the unique endemic equilibrium point is globally asymptotically stable as can be observed numerically from Fig. 7, Fig. 8, where the state variables initiating with Initial-5, Initial-6, Initial-7 and Initial-8 approach the endemic equilibrium $E^{*}=\left(10^{5},0.3\times 10^{4},2800,800,2500,900,0.6\times 10^{4},99,1.3\times 10^{5}\right)$, which agrees with Theorem 4.7. This means epidemiologically that COVID-19 could persist in Cameroon.

**Fig. 9:**
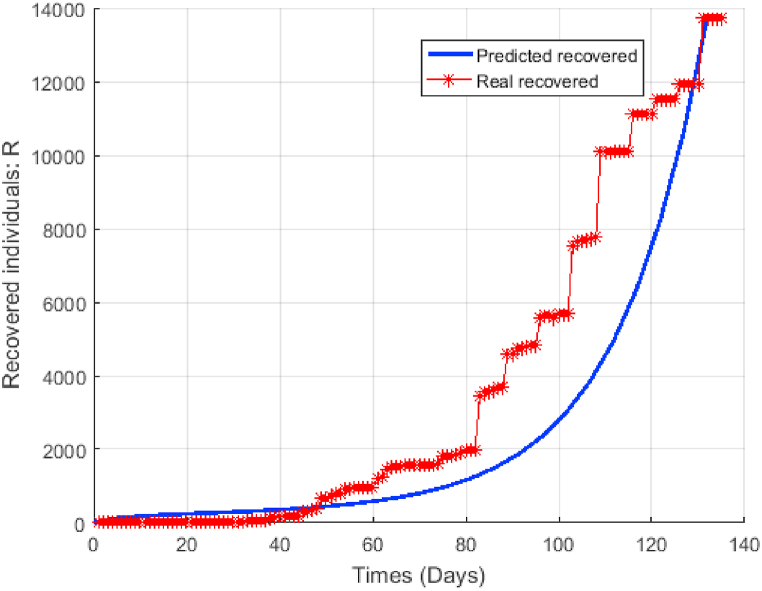
Fitted results from the COVID-19 model (2.2) using the parameter values from Table 1 except *β* = 2.08 × 10^−6^, *η*_1_ = 0.49, *a*_0_ = 10^−7^, *μ* = 1/59, *d*_1_ = 0.156986, and $R_{c}=1.6543>1$. Here, the red starred line indicates the real recovered cases and the blue line indicates the predicted recovered individuals.

Fig. 9 shows a good fit for total actual recovered individuals and those predicted by the model (2.2).

**Fig. 10:**
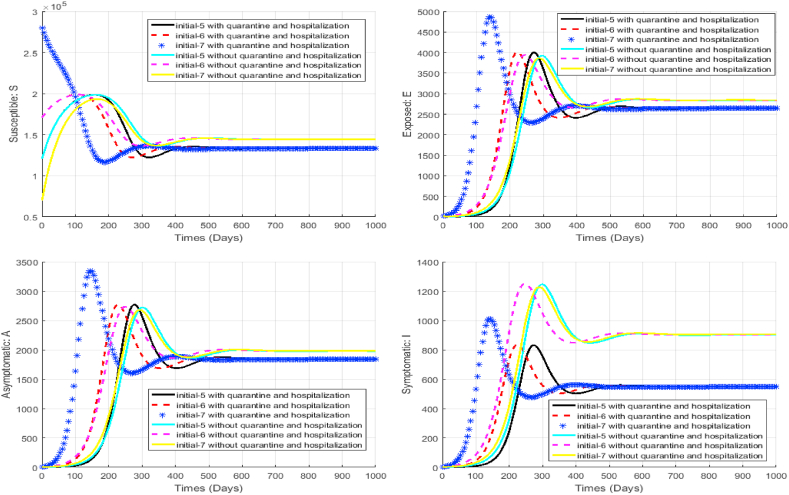
Time plots for COVID-19 model (5.1) with quarantine and hospitalization(solid line) or without quarantine and hospitalization (dashed line) using various initial conditions. The parameter values are as given in Table 1, except *β* = 1.55 × 10^−6^, *η*_1_ = 0.49, *a*_0_ = 10^−7^, *μ* = 1/59, *d*_1_ = 0.156986, and $R_{c}=1.2331>1$.

**Fig. 11:**
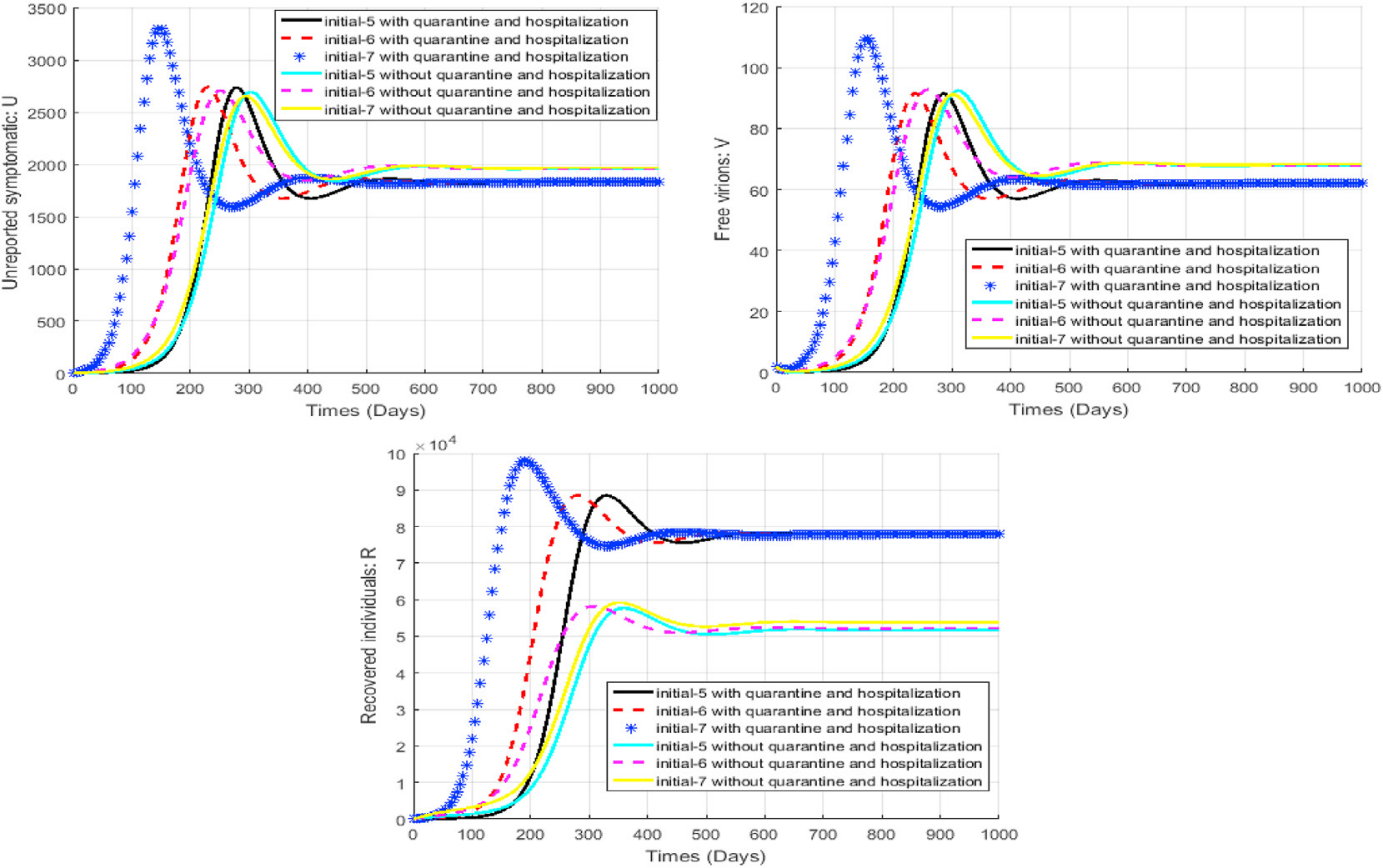
Time plots for COVID-19 model (2.2) with quarantine and hospitalization (solid line) or without quarantine and hospitalization (dashed line) using various initial conditions. The parameter values are as given in Table 1, except *β* = 1.55 × 10^−6^, *η*_1_ = 0.49, *a*_0_ = 10^−7^, *μ* = 1/59, *d*_1_ = 0.156986, and $R_{c}=1.2331>1$.

Fig. 10, Fig. 11 illustrate the magnitude of quarantine and hospitalization. From these Figures, we clearly see that if the quarantine and hospitalization are operated efficiently, the disease will reduce considerably.

**Fig. 12:**
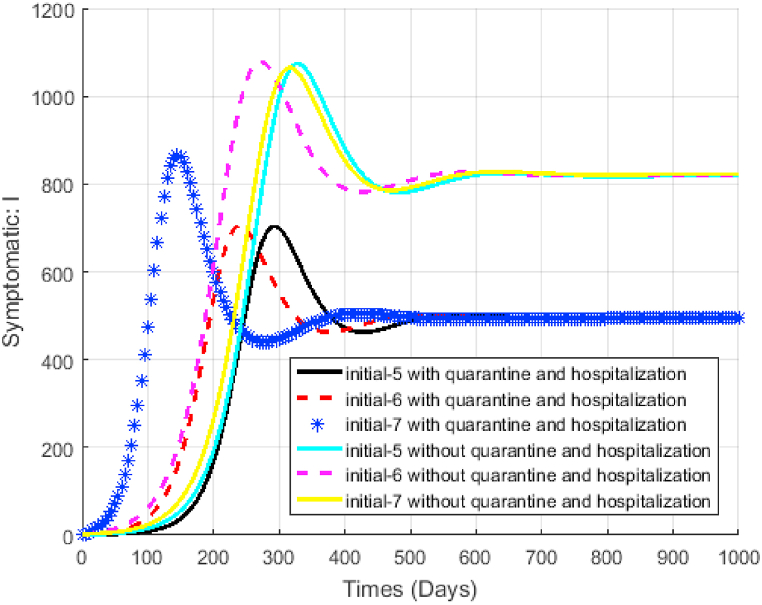
Simulation of the COVID-19 model (2.2) giving the cumulative number of new cases of infection as a function of time and using various initial conditions. The parameter values are as given in Table 1, except *β* = 1.55 × 10^−6^, *a*_0_ = 10^−7^, *μ* = 1/57, *d*_1_ = 0.999, and *η*_1_ = 0.4, so that $R_{c}=1.2241>1$, *η*_1*ϵ*_ = 0.9905, $\eta_{1d_{0}}=0.4145$ and $\eta_{1}<min\left\{\eta_{1\epsilon },\eta_{1d_{0}}\right\}$.

**Fig. 13:**
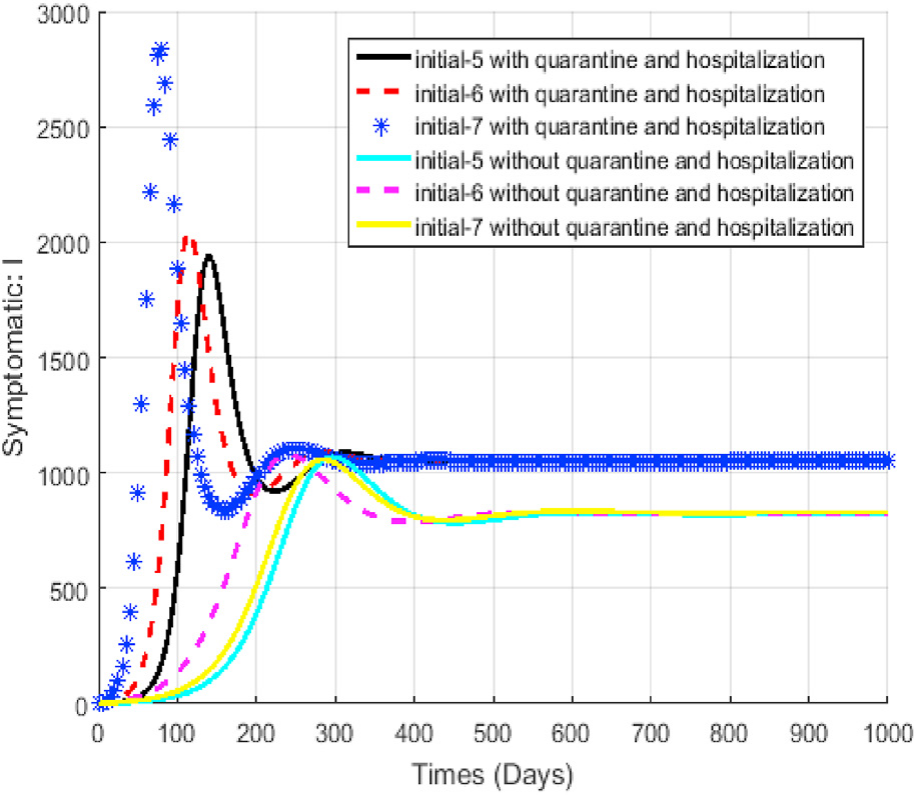
Simulation of the COVID-19 model (2.2) giving the cumulative number of new cases of infection as a function of time and using various initial conditions. The parameter values are as given in Table 1, except *β* = 1.55 × 10^−6^, *a*_0_ = 10^−7^, *μ* = 1/57, *ϵ* = 0.01, *d*_1_ = 0.999, and *η*_1_ = 0.9906, so that $R_{c}=1.5112>1$, *η*_1*ϵ*_ = 0.9905, $\eta_{1d_{0}}=0.4145$ and $\eta_{1}>max\left\{\eta_{1\epsilon },\eta_{1d_{0}}\right\}$.

Fig. 12, Fig. 13 illustrate Theorem 4.10.

We clearly observe from Fig. 12 that the cumulative number of new predicted active cases is higher when quarantine and hospitalization are not performed than when these control measures are implemented. This means that when condition $\eta_{1}<\min\left\{\eta_{1\epsilon },\eta_{1d_{0}}\right\}$ is satisfied, the use of quarantine and hospitalization could have positive impact on the community. But on Fig. 13, we see that the cumulative number of new predicted active cases is higher when quarantine and hospitalization are used than when these control measures are not implemented. This means that when condition $\eta_{1}>\max\left\{\eta_{1\epsilon },\eta_{1d_{0}}\right\}$ is satisfied, the use of quarantine and hospitalization could have negative impact to the community. The contour plots of Fig. 3 show the subordination of control reproduction number $R_{c}$ on the quarantine rate *ϵ* and the hospitalization rate *d*_0_ for Cameroon.

**Fig. 14:**
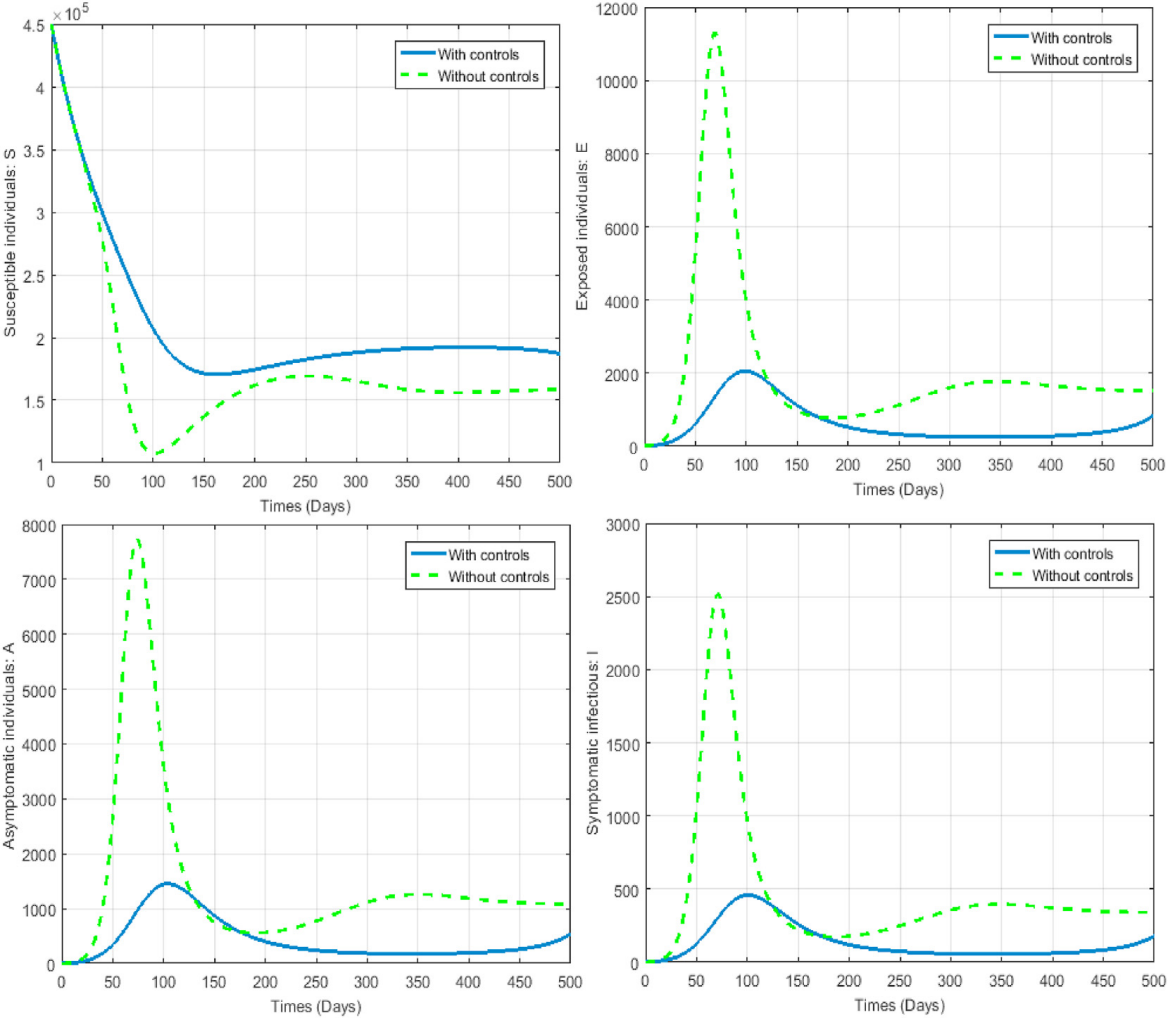
Time plots for COVID-19 model (5.1) with control (solid line) or without control (dashed line). The parameter values are as given in Table 1, except *γ*_1_ = 0.7, *γ*_2_ = 0.5, *β* = 1.55 × 10^−6^, *η*_1_ = 0.49, *a*_0_ = 10^−7^, *μ* = 1/57.

**Fig. 15:**
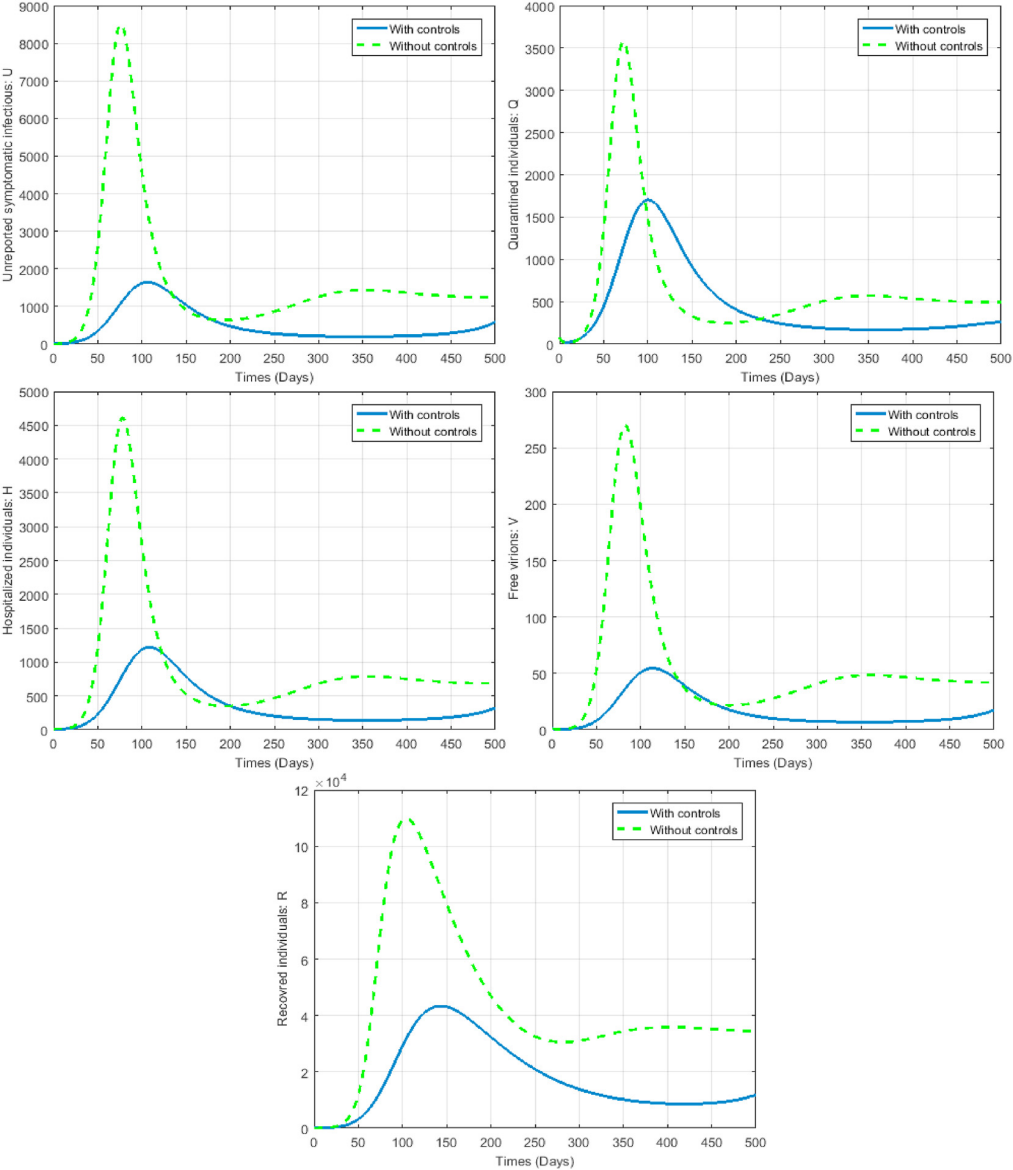
Time plots for COVID-19 model (5.1) with control (solid line) or without control (dashed line). The parameter values are as given in Table 1, except *γ*_1_ = 0.7, *γ*_2_ = 0.5, *β* = 1.55 × 10^−6^, *η*_1_ = 0.49, *a*_0_ = 10^−7^, *μ* = 1/57.

Finally, the optimality system constituted of the established state equation (5.1), adjoint equation (5.3), control characterization (5.4)–(5.6) and corresponding initial and final conditions are carried out by using the forward-backward method. The algorithm starts by solving the state variables equations with a guess for the controls over the simulated time using an iterative method with forward fourth order Runge Kutta scheme. The state variables system with an initial guess is solved forward in time and then the adjoint system (5.3) is solved backward in time by a backward fourth order Runge Kutta scheme. This iterative process breaks off when the current state, adjoint, and control values converge sufficiently. Here, we choose the initial condition (*S*(0), *E*(0), *A*(0), *I*(0), *U*(0), *Q*(0), *H*(0), *V*(0), *R*(0)) = (450000, 8, 0, 0, 0, 80, 10, 1, 0) to illustrate the control strategies. We choose the upper bound *b*_1_ of *u*_1_ equal to 0.8, owing to the reasonable case in Cameroon that it took at least average 3 days to quarantine people who have been exposed to COVID-19. We choose the upper bound *b*_2_ of *u*_2_ similarly to *u*_1_ and the upper bound *b*_3_ of *u*_3_ equal to 0.7. Considering the weight coefficients associated with *E*, *A*, *I*, *U*, *Q*, *H* and *V*, we choose *B*_1_ = 100, *B*_2_ = 500, *B*_3_ = 2000, *B*_4_ = 700, *B*_5_ = 100, *B*_6_ = 1500, *B*_7_ = 800, *R*_1_ = 3.5 × 10^7^, *R*_2_ = 10^7^ and *R*_3_ = 2.5 × 10^8^ to illustrate the optimal strategies. We suppose that the weight coefficient *R*_3_ associated with control *u*_3_ is greater than *R*_1_ and *R*_2_ which have close values associated with the controls *u*_1_ and *u*_2_, respectively. These assumptions are based on the fact that: the cost associated with *u*_1_ includes the cost of monitoring and quarantining schedule, and the cost associated with *u*_2_ includes the cost of hospital special medical treatment resource, while the cost associated with *u*_3_ includes the cost of hydro-alcoholic gel, disinfectant products and face masks. We observe on Fig. 14, Fig. 15 that when the controls are used, the unreported symptomatic infectious individuals cases decrease faster than when the strategies are not applied. Moreover, in the presence of control measures, we have less infectious individuals than in the absence of the control. Also, the compliance with barrier measures such as the regular washing of hands, the use of hydro-alcoholic gel, wearing face masks, social distancing rules and disinfected surfaces can significantly reduce the number of infected and infectious individuals as well as the concentration of virus in the environment. Thus, the disease could infect a large part of the population if these measures are not followed.

**Fig. 16:**
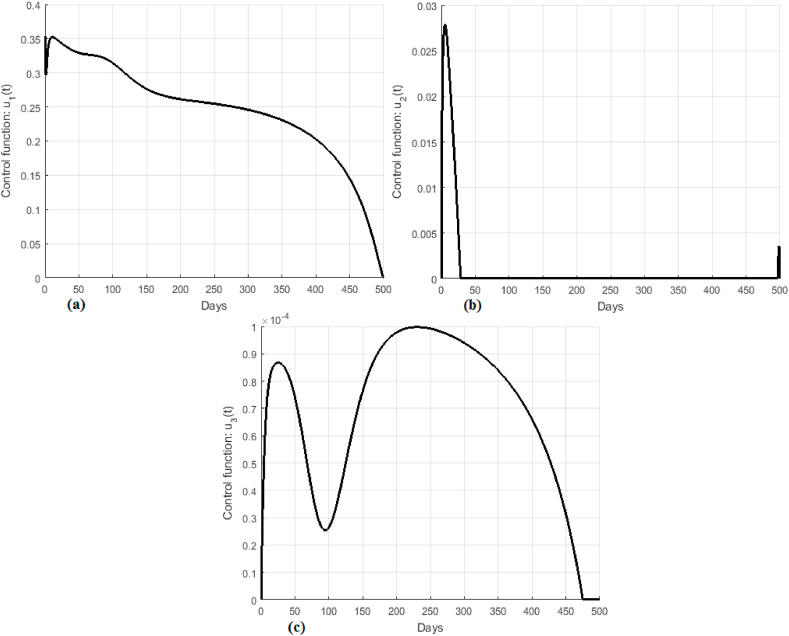
The optimal control profiles (a) *u*_1_(*t*), (b) *u*_2_(*t*) and (c) *u*_3_(*t*) with *γ*_1_ = 0.7, *γ*_2_ = 0.5, *B*_1_ = 100, *B*_2_ = 500, *B*_3_ = 2000, *B*_4_ = 700, *B*_5_ = 100, *B*_6_ = 1500, *B*_7_ = 800, *R*_1_ = 3.5 × 10^7^, *R*_2_ = 10^7^ and *R*_3_ = 2.5 × 10^8^.

Fig. 16 depicts the extremal control behaviour of *u*_1_, *u*_2_ and *u*_3_. In order to minimize the total infected individuals, *E* + *A* + *I* + *U* + *Q* + *H* and the concentration of virus in the environment, *V*, the optimal control *u*_1_ stays at its upper bound for a short time, approximately 20 days and then steadily decreases to the lower bound in the remaining simulated time. Meanwhile, the optimal control *u*_2_ starts at a lower level value zero, steadily increases to its upper bound and stays for short time, about 10 days, then steadily decreases to the lower bound in the simulated time until 500 days and, at the end, increases again to the level value (0.003). In the meantime, the optimal control *u*_3_ also starts at a lower level value zero, steadily increases to an upper level value (8.7 × 10^−5^) and stays for a short time, nearly up to 25 days, then is tapered off to a lower level (2.5 × 10^−5^), and increases to its upper bound where it stays during two months and finally decreases steadily to the lower bound over the remaining simulated time.

Note that at the beginning of simulated time, the optimal control *u*_1_ is staying at its upper bound in order to quarantine many exposed individuals (*E*) to prevent the increasing of the number of the infected classes. But at the beginning of simulated time, the optimal control *u*_2_ seems to start by tracing, testing and then reaches its upper bound where it stays in order to hospitalize many symptomatic infectious individuals (*I*) to prevent the increasing of the number of people with clinical symptoms. Now, we see on Fig. 16 (c) that the optimal control *u*_3_ implements the global effort of educational campaigns that run for over 250 days in order to prevent the increasing of the concentration of virus in the environment.

**Fig. 17:**
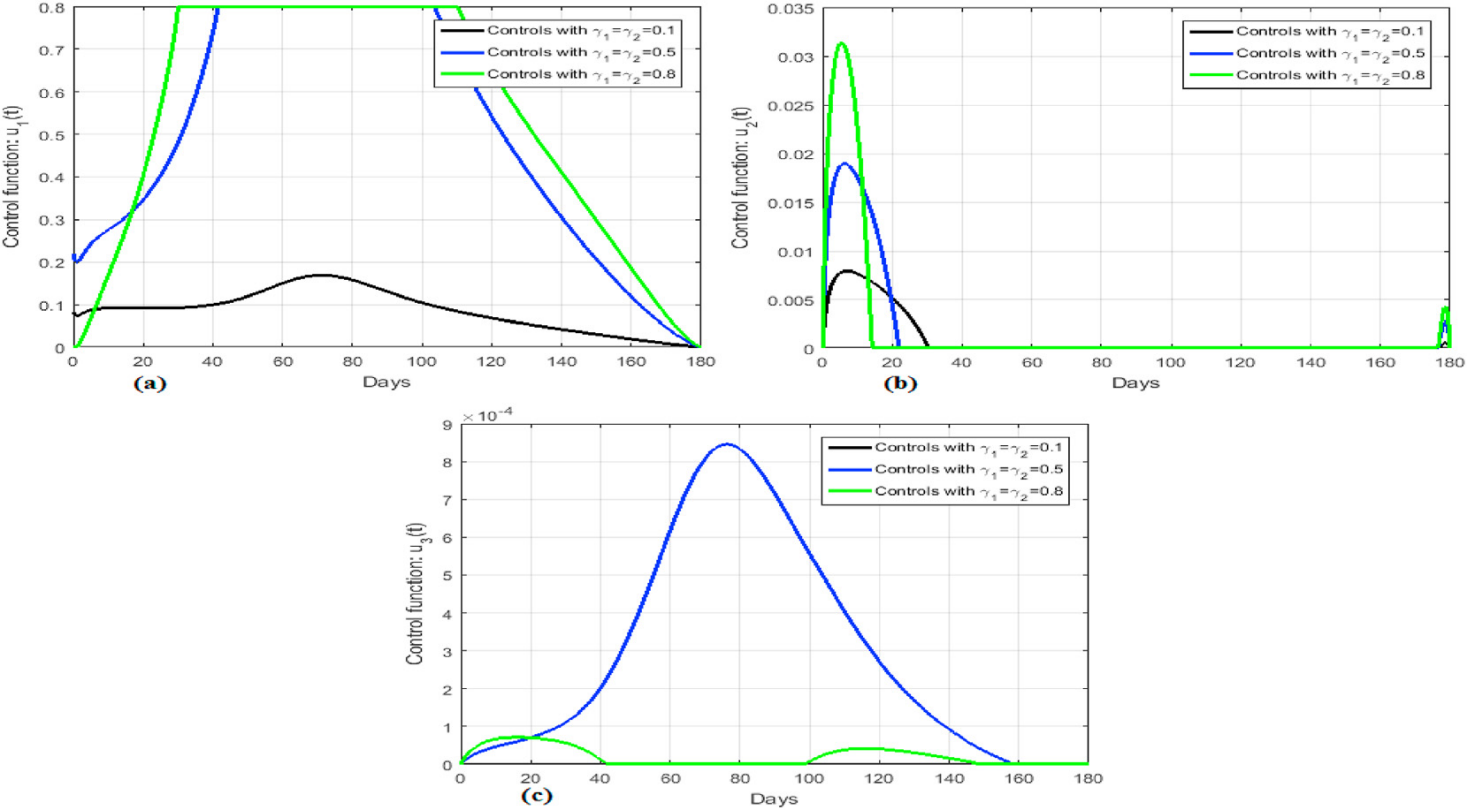
The optimal control profiles (a) *u*_1_(*t*), (b) *u*_2_(*t*) and (c) *u*_3_(*t*) with *B*_1_ = 100, *B*_2_ = 500, *B*_3_ = 2000, *B*_4_ = 700, *B*_5_ = 100, *B*_6_ = 1500, *B*_7_ = 800, *R*_1_ = 3.5 × 10^7^, *R*_2_ = 10^7^ and *R*_3_ = 2.5 × 10^8^.

Fig. 17 shows the time dependent optimal control *u*_1_, *u*_2_ and *u*_3_, for different values of special medical treatment rate *γ*_1_ and mandatory quarantine rate *γ*_2_. From this figure, we can see that the higher the values of *γ*_1_ and *γ*_2_, the more effective the controls *u*_1_ and *u*_2_ are, while the control *u*_3_ is effective only at 50% of these values.

**Fig. 18:**
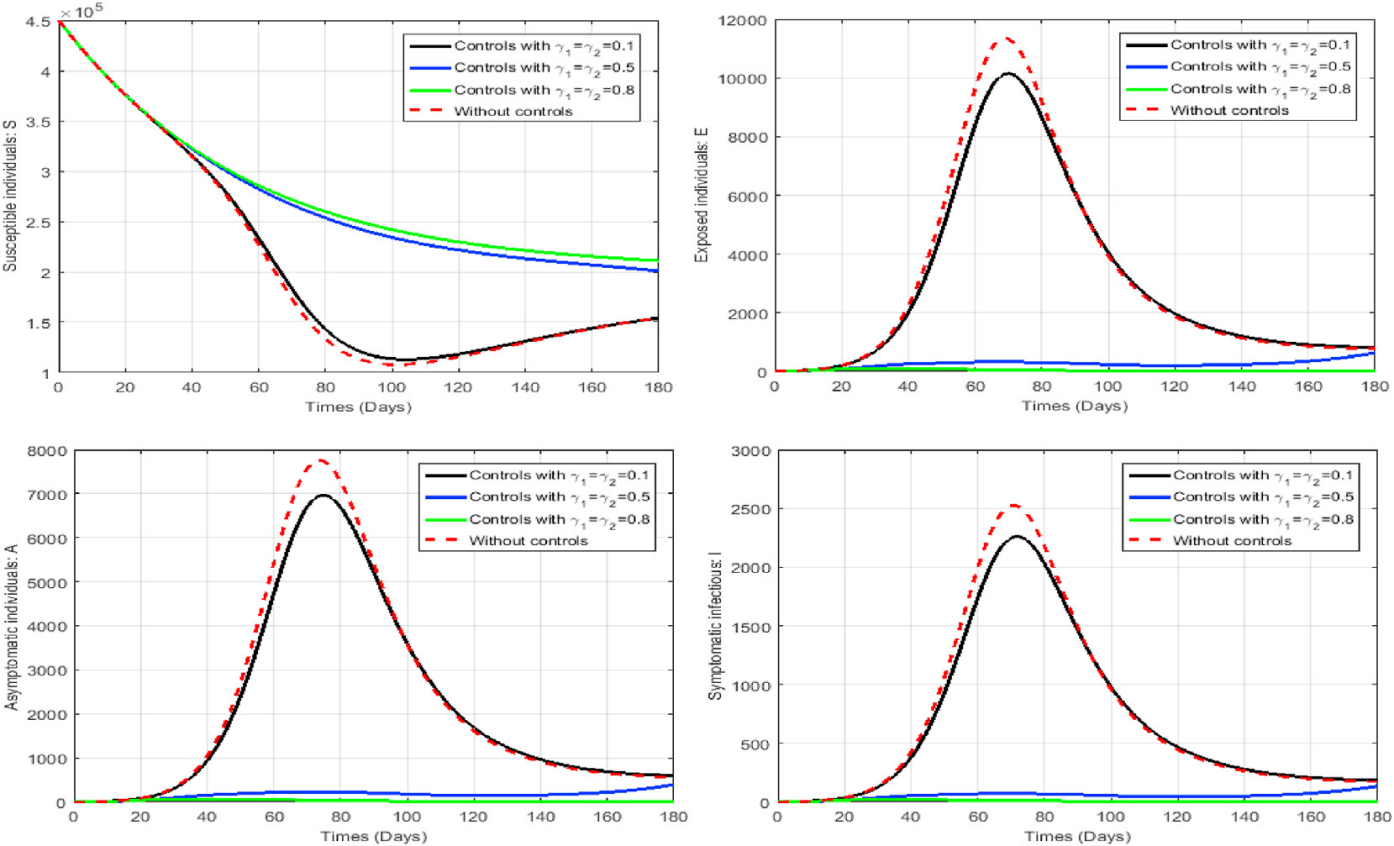
Time plots for COVID-19 model (5.1) with control (solid lines) or without control (dashed lines). The parameter values are as given in Table 1, except *β* = 1.55 × 10^−6^, *η*_1_ = 0.49, *a*_0_ = 10^−7^, *μ* = 1/57.

**Fig. 19:**
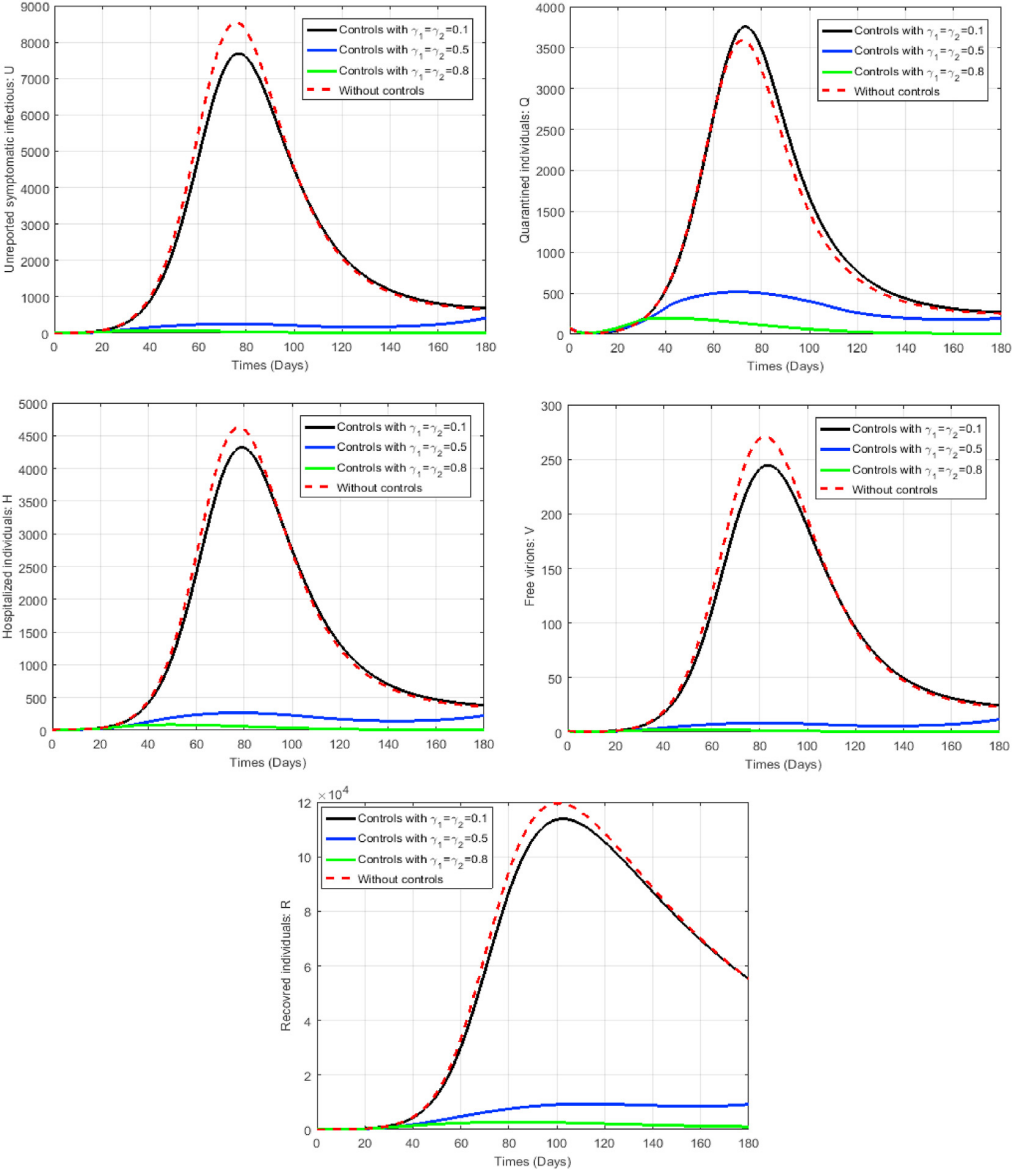
Time plots for COVID-19 model (5.1) with control (solid lines) or without control (dashed lines). The parameter values are as given in Table 1, except *β* = 1.55 × 10^−6^, *η*_1_ = 0.49, *a*_0_ = 10^−7^, *μ* = 1/57.

Fig. 18, Fig. 19 illustrate how optimal control strategies change as the special medical treatment rate *γ*_1_ and mandatory quarantine rate *γ*_2_ vary. These Figures confirm that from 50% of the value of *γ*_1_ and *γ*_2_, one could expect a considerable reduction of the infection in the community.

**Fig. 20:**
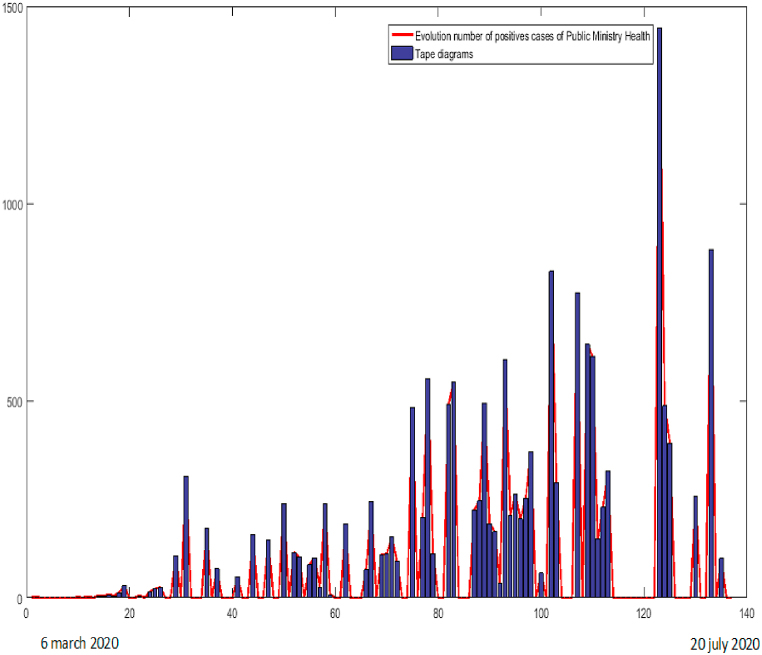
Evolution number of positive cases. Source: Cameroon Ministry of Public Health.

Fig. 20 represents the evolution number of positive cases in Cameroon from March 6 to July 20, 2020.

## 7 Conclusion

In this paper, to understand the transmission dynamics of COVID-19 in Cameroon, we formulated a compartmental ordinary differential equations model. A particular stress has been placed on quarantine and hospitalized classes. More precisely, we studied the impact of quarantine and hospitalization on curtailing the spread of the disease. The model is completely analyzed and the strategies for effective control of the progress of the disease are suggested. Using the method developed by van den Driessche and Wattmough (van den Driessche and Watmough, 2002), we obtained the control reproduction number $R_{c}$ of the model. We constructed a suitable Lyapunov function to prove that system (2.2) has a globally asymptotically stable disease-free equilibrium whenever the control reproduction number is less than unity. When the control reproduction number exceeds unity, the disease-free equilibrium loss its stability and gives rise to a unique endemic equilibrium. By a skillful construction of a suitable Lyapunov function we proved that the endemic equilibrium is globally asymptotically stable. The efficiency of the quarantine of exposed cases and the isolation of hospitalized cases is dependent on the size of the modification parameter for the reduction of infectiousness of hospitalized individuals *η*_1_. It is shown that the use of quarantine and hospitalization could have positive impact on the population if $\eta_{1}<\min\left\{\eta_{1\epsilon },\eta_{1d_{0}}\right\}$, no impact if $\eta_{1}=\min\left\{\eta_{1\epsilon },\eta_{1d_{0}}\right\}$, and harmful impact if $\eta_{1}>\max\left\{\eta_{1\epsilon },\eta_{1d_{0}}\right\}$. Adding to this investigation the optimal control problem, we suggest quarantine and hospitalization as good strategies for controlling the disease. Note that COVID-19 is still ongoing in Cameroon and in many other countries in the world. This investigation attempt to provide Cameroonian authorities with some in-depth understanding of the disease dynamics so as to help them take better decisions for fighting against this highly deadly pandemic.

## Funding

The work received no funding.

## Data availability statement

The codes written to run most of the simulations presented in this work can be available upon simple request to the authors.

## Declaration of interests

The authors declare that they have no known competing financial interests or personal relationships that could have appeared to influence the work reported in this paper.
